# Receptor deorphanization in starfish reveals the evolution of relaxin signaling as a regulator of reproduction

**DOI:** 10.1186/s12915-025-02158-2

**Published:** 2025-02-25

**Authors:** Yuling Feng, Nayeli Escudero Castelán, Mohammed Akhter Hossain, Hongkang Wu, Hidekazu Katayama, Stuart J. Smith, Scott F. Cummins, Masatoshi Mita, Ross A. D. Bathgate, Maurice R. Elphick

**Affiliations:** 1 Centre for Evolutionary & Functional Genomics, School of Biological & Behavioural Sciences, Queen Mary University of London, London, E1 4NS UK; 2 Florey Institute of Neuroscience and Mental Health and Department of Biochemistry and Pharmacology, University of Melbourne, Parkville, VIC 3010 Australia; 3 Utsunomiya Chanpus Liberal Arts Center, Teikyo University, 1-1 Toyosatodai, Utsunomiya, Tochigi, 320-8551 Japan; 4 Centre for Bioinnovation and School of Science, Technology and Engineering, University of the Sunshine Coast, Maroochydore, QLD Australia; 5 Department of Biochemistry, Showa University School of Medicine, Shinagawa-Ku, Tokyo, 142-8555 Japan

**Keywords:** Relaxin, Starfish, Receptor, Reproduction, Evolution

## Abstract

**Background:**

Relaxins are a family of peptides that regulate reproductive physiology in vertebrates. Evidence that this is an evolutionarily ancient role of relaxins has been provided by the discovery of two relaxin-like gonad-stimulating peptides (RGP1 and RGP2) that trigger spawning in starfish. The main aim of this study was to identify the receptor(s) that mediate(s) the effects of RGP1 and RGP2 in starfish.

**Results:**

Here we show that RGP1 and RGP2 belong to a family of peptides that include vertebrate relaxins, *Drosophila* insulin-like peptide 8 (Dilp8), and other relaxin-like peptides in several protostome taxa. An ortholog of the human relaxin receptors RXFP1 and RXFP2 and the *Drosophila* receptor LGR3 was identified in starfish (RXFP/LGR3). In *Drosophila*, but not in humans and other vertebrates, there is a paralog of LGR3 known as LGR4, and here an LGR4-type receptor was also identified in starfish. In vitro pharmacological experiments revealed that both RGP1 and RGP2 act as ligands for RXFP/LGR3 in the starfish *Acanthaster *cf.* solaris* and *Asterias rubens*, but neither peptide acts as a ligand for LGR4 in these species.

**Conclusions:**

Discovery of the RXFP/LGR3-type receptor for RGP1 and RGP2 in starfish provides a new insight into the evolution of relaxin-type signaling as a regulator of reproductive processes. Furthermore, our findings indicate that RXFP/LGR3-type receptors have been lost in several phyla, including urochordates, mollusks, bryozoans, platyhelminthes, and nematodes.

**Supplementary Information:**

The online version contains supplementary material available at 10.1186/s12915-025-02158-2.

## Background

The hormone relaxin was first discovered as a substance present in the blood of pregnant guinea pigs and rabbits that causes relaxation of the interpubic ligament [1] and further analysis revealed it to be a peptide derived from the corpus luteum of ovaries [2]. However, it was not until the 1970s that the molecular structure of relaxin was determined, identifying it as an insulin-like molecule comprising two peptides (A-chain, B-chain) linked by two interchain disulfide bridges and one intrachain disulfide bridge [3, 4]. Subsequently, a family of relaxin-type peptides has been identified in mammals, which include relaxin1–3 (RLN1–3) and insulin-like peptides 3–6 (INSL3–6) in humans. Furthermore, analysis of the expression and functions of these peptides has revealed roles in the regulation of reproduction and a variety of other physiological processes, including gastrointestinal and cardiovascular function, metabolism, and stress [5].

Discovery of the mechanism of action of relaxin-type peptides was enabled by the observation that the phenotype of abnormal testis descent is observed in gene-knockout mice for both INSL3 and LGR8, a leucine-rich repeat-containing G-protein coupled receptor (GPCR). Thus, it was hypothesized that LGR8 is a relaxin receptor and this was confirmed by experimental studies demonstrating that INSL3 acts as a ligand for LGR8 [5, 6]. Furthermore, a second leucine-rich repeat-containing GPCR known as LGR7 was found to be the receptor for RLN2, and orphan GPCRs known as GPCR135 and GPCR142 were found to act as receptors for RLN3 and INSL5, respectively. Accordingly, LGR8, LGR7, GPCR135, and GPCR142 are now referred to as relaxin family peptide (RXFP) receptors 1–4, respectively [5]. The focus of this study is on relaxin-type signaling and it is noteworthy that, within the broader family of insulin-related peptides, relaxin-type peptides are unique in exerting their physiological effects by binding to GPCRs. This contrasts with insulin and insulin-like growth factors (IGFs), which exert their effects by binding to tyrosine kinase-type receptors [7].

Insights into the evolution and comparative physiology of relaxin signaling have been obtained with the discovery and functional characterization of relaxin/insulin-type peptides and their receptors in the fruit fly *Drosophila melanogaster*. Thus, human RXFP1&2 are co-orthologs of the *Drosophila* receptor LGR3, and experimental studies have demonstrated that a relaxin/insulin-like peptide known as *Drosophila* insulin-like peptide 8 (Dilp8 or gonadulin) acts as a ligand for LGR3 [8]. Furthermore, functional studies indicate that Dilp8-LGR3 signaling regulates growth, maturation, and reproductive behavior in *Drosophila* [8–14]. Interestingly, it has recently been reported that a second insulin-like peptide in *Drosophila* known as Dilp7 acts as a ligand for LGR4, a GPCR that is closely related to LGR3, and Dilp7-LGR4 signaling is required for noxious light avoidance in *Drosophila* larvae [15]. Thus, relaxin/insulin-related peptides that act as ligands for GPCRs are evolutionarily ancient regulators of diverse processes, ranging from reproductive maturation and function to escape behavior. However, to obtain a broader comparative perspective, an investigation of the occurrence and function of GPCR-mediated relaxin/insulin-type signaling in other invertebrate taxa is needed.

A key insight into the evolution of relaxin-type signaling as a regulator of reproductive processes was the identification of a gonadotropic neuropeptide in starfish (phylum Echinodermata) that is a relaxin-like peptide [16]. The existence of a gonadotropic substance in starfish was first reported in 1959 with the observation that extracts of radial nerve cords trigger spawning when injected into reproductively mature animals [17]. The gonadotropin was named gonad-stimulating substance (GSS) and subsequent studies revealed it to be a peptide that exerts its effects by stimulating ovarian follicle cells surrounding oocytes to produce 1-methyladenine, which then triggers germinal vesicle breakdown and spawning [18]. Fifty years after its existence was first reported, GSS was finally identified in the starfish *Patiria pectinifera* as a relaxin-type peptide and it was then renamed relaxin-like gonad-stimulating peptide (RGP) [16, 18, 19]. Subsequently, RGP has been identified in other starfish species and has also been shown to have gonadotropic activity [18, 20]. Furthermore, a recent detailed anatomical analysis of RGP in the starfish *Asterias rubens* revealed a widespread pattern of expression, including the radial nerve cords, digestive system, body wall-associated organs, and gonoducts. Informed by these findings, it was hypothesized that the release of RGP from the gonoducts triggers gamete maturation and spawning in starfish, while RGP produced in other parts of the body may regulate other physiological/behavioral processes [21].

Interestingly, a cDNA encoding the precursor of a second relaxin-type peptide has been identified in the starfish *A. rubens* by analysis of transcriptome sequence data and named relaxin-like peptide 2 (ArRLP2) [22]. Subsequently, orthologs of ArRLP2 have been identified in other starfish species, indicating that the occurrence of two relaxin-type peptides is an evolutionarily conserved feature of the class Asteroidea [23]. Furthermore, in vitro pharmacological tests on ovarian tissue from two starfish species, *A. rubens* and *P. pectinifera*, have revealed that RLP2-type peptides mimic the gonadotropic action of RGP and induce oocyte maturation and spawning. Therefore, RGP and RLP2 have been renamed as RGP1 and RGP2, respectively [24].

The discovery that two relaxin-like peptides (RGP1 and RGP2) act as gonadotropins in starfish provided a basis for the aims of this study. Firstly, to investigate the evolution of these neuropeptides by performing a detailed analysis of the phylogenetic distribution and relationships of relaxin-type precursors in echinoderms and other bilaterian phyla. Secondly, to investigate the mechanisms by which RGP1 and RGP2 exert their gonadotropic effect in starfish by identifying candidate receptor(s) for these peptides, which was accomplished by analysis of transcriptome/genome sequence data to determine the phylogenetic relationships and structures of genes encoding relaxin-type receptors in echinoderms and in other bilaterian phyla. Having identified two relaxin receptor-related GPCRs in starfish, our third aim was to experimentally test both RGP1 and RGP2 as ligands for these receptors. The findings of this study provide new insights into the evolution of relaxin-type signaling as a regulator of reproductive physiology.

## Results

### Relationships of starfish RGP1 and RGP2 with relaxin/insulin/IGF-type peptides in other taxa

**Fig. 1:**
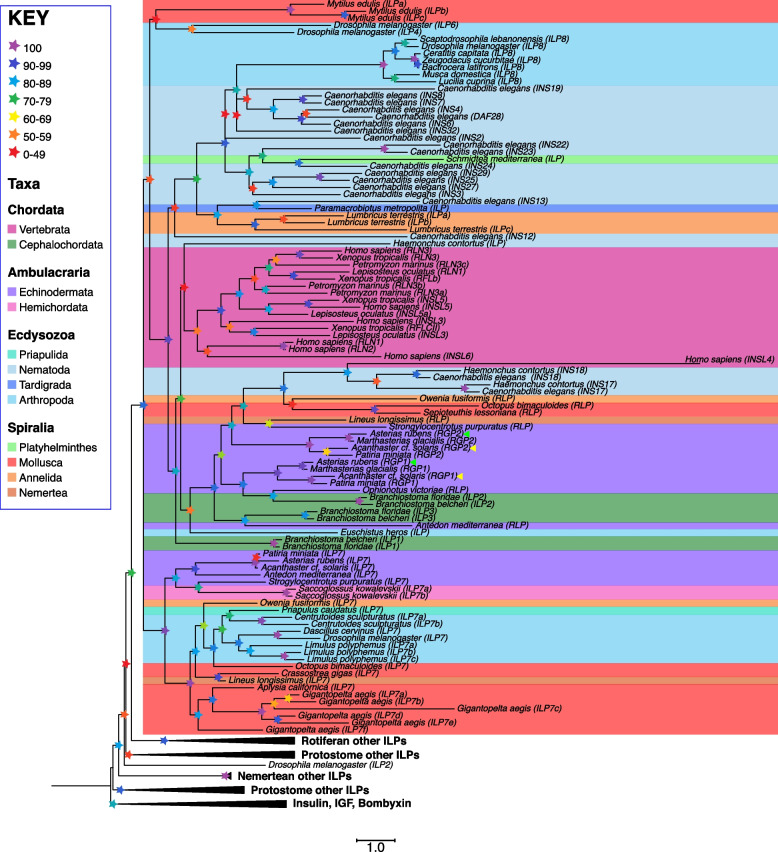
Phylogenetic analysis of relaxin/insulin/IGF-type peptide precursors. The tree was generated in IQ-tree v 2.3.6 using the maximum likelihood method (SH-aLRT) and rooted using a clade comprising insulin-, IGF-, and bombyxin-type precursors. The clade that includes relaxin/Dilp8-type precursors and Dilp7-type precursors is shown without concatenation and the branches in this clade are highlighted according to the phyla that species belong to, following the color code shown in the key. Other clades are concatenated. The *A. rubens* RGP1 and RGP2 precursors are labeled with a green arrowhead and the *A. *cf.* solaris* RGP1 and RGP2 precursors are labeled with a yellow arrowhead. Bootstrap support values are color-coded with stars as indicated in the key. The accession numbers and sequences of the precursor proteins included in this phylogenetic tree are listed in Additional file 10: Dataset S3 and Additional file 11: Dataset S4, respectively

To investigate the evolution of starfish RGP1 and RGP2 and their phylogenetic relationships with relaxin/insulin/IGF-type peptides in other taxa, the sequences of relaxin/insulin/IGF-type precursors were identified in bilaterian taxa. Then both CLANS and phylogenetic analysis were employed to investigate relationships between relaxin/insulin/IGF-type peptide precursors in the Bilateria. CLANS revealed that the starfish RGP1 and RGP2 precursors and the precursors of relaxin-like peptides in other echinoderms cluster with chordate relaxin-type precursors (Additional File 1: Fig. S1). Phylogenetic analysis of bilaterian relaxin/insulin/IGF-type precursors using a maximum likelihood method revealed that echinoderm precursors of relaxin-like peptides (including starfish RGP1 and RGP2) and chordate relaxin-type precursors are positioned in the same clade and this clade also comprises precursors of relaxin-like peptides from a variety of protostome phyla, including *Drosophila* Dilp8. Furthermore, phylogenetic analysis revealed that within the wider family of relaxin/insulin/IGF-type peptide precursors, relaxin/Dilp8-type precursors are most closely related to Dilp7-type precursors (Fig. 1).

**Fig. 2:**
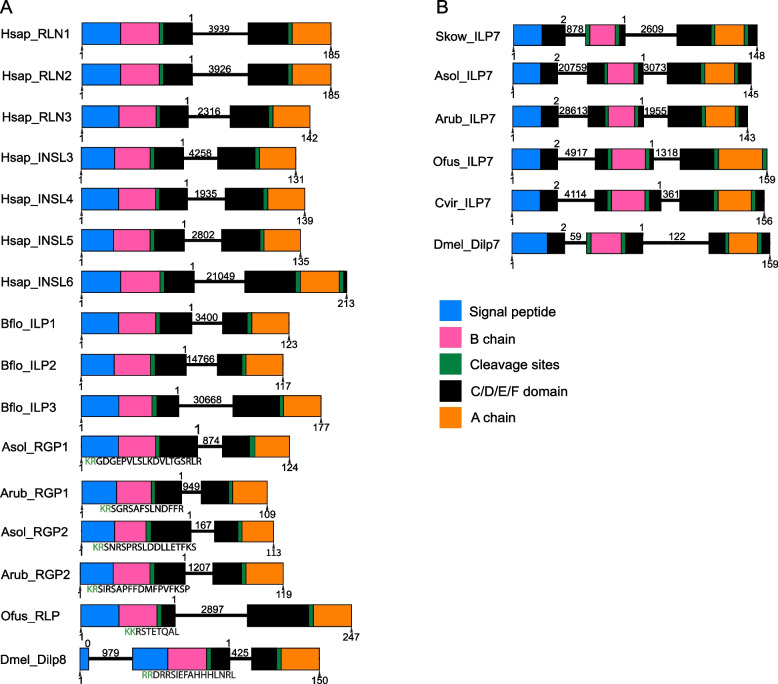
Comparison of the exon/intron structure of genes encoding relaxin/Dilp8-type precursors and Dilp7-type precursors in selected bilaterian species. Schematic representations of the gene structures are shown, with protein-coding exons displayed as rectangles and introns shown as lines (with intron length stated above). The phase of introns is stated at the start of each intron above each diagram. Protein-coding exons are color-coded to show regions that encode the N-terminal signal peptide (blue), the B-chain (pink), monobasic or dibasic cleavage sites (green), the C-chain and other regions of the precursors (black), and the A-chain (orange). The residue numbers of the first and last amino acids are labeled underneath each diagram. **A** Genes encoding relaxin-type precursors in chordates, the starfish *A. *cf.* solaris* and *A. rubens* (RGP1 and RGP2), the annelid *O. fusiformis* and *D. melanogaster* (Dilp8) have a conserved phase 1 intron that interrupts the coding sequence for the C-chain region. **B** Genes encoding Dilp7-type precursors in the hemichordate S. *kowalevskii*, the starfish *A. *cf.* solaris* and *A. rubens*, the annelid *O. fusiformis*, the mollusk *C. virensis*, and the arthropod *D. melanogaster* (Dilp7) also have a conserved phase 1 intron that interrupts the coding sequence for the C-chain region, like that found in relaxin/Dilp8-type precursor genes. However, in addition, the Dilp7-type precursor genes have a second intron (phase 2) located between exons that encode the signal peptide and B-chain and this feature distinguishes Dilp7-type precursor genes from relaxin/Dilp8-type precursor genes. Accordingly, unlike in relaxin/Dilp8-type precursors where the B-chain is located immediately after the signal peptide, in Dilp7-type precursors the signal peptide and B-chain are separated by a polypeptide sequence (shown in black). The accession numbers and sequences of precursor proteins included in this figure are listed in Additional file 14: Dataset S7

Another approach employed here to investigate the relationships of starfish RGP1 and RGP2 precursors with relaxin/insulin-type precursors in other taxa was to investigate the structure of the genes encoding these precursors because the position and phase of introns can be an evolutionarily conserved and distinguishing feature of orthologous genes. This revealed in the starfish (*A.* cf. *solaris*, *A. rubens*) RGP1 and RGP2 precursor genes the presence of a phase 1 intron that interrupts the coding sequence at a position located between an exon that encodes the signal peptide, the B-chain, and the N-terminal region of the C-peptide and an exon that encodes the C-terminal region of the C-peptide and the A-chain. Accordingly, an intron was found in the same position in genes encoding relaxin-type precursors in other taxa (Fig. 2A). By way of comparison, genes encoding Dilp7-type peptide precursors have a phase 1 intron in the same position but these genes also have a second intron (phase 2) that interrupts the coding sequence at a position located between an exon that encodes the signal peptide and an exon that encodes the B-chain (Fig. 2B). Thus, the presence or absence of this intron distinguishes Dilp7-type precursors and relaxin/Dilp8-type precursors, respectively.

Lastly, we also analyzed the sequences of the A- and B-chains of starfish RGP1 and RGP2 in comparison with the sequences of the A- and B-chains of relaxin/Dilp8-type peptides in other taxa. Interestingly, this revealed the presence of an alanine residue in RGP2 B-chains that is not present in the B-chain of RGP1-type peptides in starfish or relaxin-type peptides in other echinoderms. However, an alanine residue is present in the same position in *Drosophila* Dilp8 and a relaxin-type peptide in the annelid *Owenia fusiformis*. Furthermore, a Gly-Ile (GI) motif is a feature of the A-chain in starfish RGP2-type peptides that is also present in the *O. fusiformis* A-chain, but which is not present in starfish RGP1-type peptides or relaxin-type peptides in other taxa (Additional file 2: Fig. S2).

### Identification of a candidate receptor for RGP1 and RGP2 in starfish

**Fig. 3:**
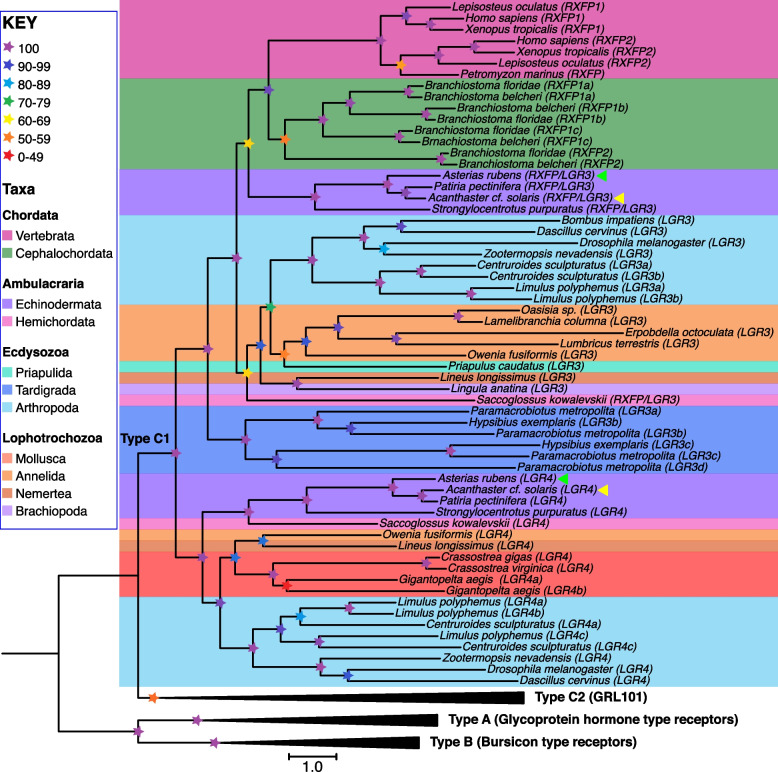
Phylogenetic analysis of leucine-rich repeat type G-protein coupled receptors (LRR-GPCRs). LRR-GPCRs are positioned in three distinct clades: type A includes glycoprotein hormone-type receptors (concatenated in this tree), type B includes bursicon-type receptors (concatenated in this tree), and type C includes relaxin-type receptors (type C1; not concatenated in this tree) and the GRL101-type receptors (type C2; concatenated in this tree). The type C1 receptors in the starfish *A. *cf.* solaris* include two receptors that are labeled with yellow arrows: Firstly, AsolRXFP/LGR3, which is an ortholog of vertebrate RXFP1/RXFP2-type relaxin receptors and *Drosophila* LGR3. Secondly, AsolLGR4, which is an ortholog of *Drosophila* LGR4. Likewise, the type C1 receptors include two receptors in the starfish *A. rubens* that are labeled with green arrows: Firstly, ArubRXFP/LGR3, which is an ortholog of vertebrate RXFP1/RXFP2-type relaxin receptors and *Drosophila* LGR3. Secondly, ArubLGR4, which is an ortholog of *Drosophila* LGR4. Based on this phylogenetic analysis, we identified AsolRXFP/LGR3 and ArubRXFP/LGR3 as candidate receptors for RGP1 and RGP2 in *A. *cf.* solaris* and *A. rubens*. The tree was generated in IQ-tree v 2.3.6 using the maximum likelihood method (SH-aLRT), with the clade comprising type A and type B receptor sequences selected as an outgroup to root the tree. The colored stars represent bootstrap support (1000 replicates, see key) and the colored backgrounds represent different taxonomic groups as also shown in the key. The accession numbers and sequences of the receptors included in this phylogenetic tree are listed in Additional file 12: Dataset S5 and Additional file 13: Dataset S6, respectively

Having established that starfish RGP1 and RGP2 belong to a bilaterian family of relaxin/Dilp8-type peptides, we sought to identify candidate receptors for RGP1 and RGP2 in *A. *cf.* solaris* and *A. rubens*. To accomplish this, we investigated the occurrence in starfish and other echinoderms of homologs of the mammalian relaxin-type receptors RXFP1–4. BLAST analysis of transcriptome/genome sequence data did not reveal the presence of RXFP3/RXFP4-type receptors, but homologs of mammalian leucine-rich repeat type RXFP1/RXFP2-type GPCRs and *Drosophila* LGR3/LGR4-type receptors were identified in starfish and other echinoderms. Thus, two genes encoding RXFP1-2/LGR3/LGR4-type receptors were identified in both *A. *cf.* solaris* (LOC110976116; LOC110984685) and *A. rubens* (LOC117293693; LOC117296788) and homologs of these were identified in other echinoderms. To investigate relationships more specifically between these echinoderm receptors and leucine-rich repeat-type GPCRs more generally in the Bilateria, we performed CLANS analysis of the sequences (Additional file 3: Fig. S3) and generated a phylogenetic tree using the maximum-likelihood method (Fig. 3).

Both CLANS and phylogenetic analysis revealed that leucine-rich repeat type GPCRs sub-divide into three distinct types. Firstly, type A includes glycoprotein hormone receptors. Secondly, type B includes human LGR4–6 and insect bursicon receptors. Thirdly, type C further sub-divides into type C1, which includes relaxin-type receptors, and type C2, which includes bilaterian homologs of the orphan receptor GRL101, which was first identified in the mollusk *Lymnaea stagnalis* (Fig. 3; Additional file 3: Fig. S3). A more detailed analysis of the type C1 clade revealed that it further sub-divides into two distinct clades. Firstly, a clade that includes mammalian RXFP1/RXFP2 receptors and *Drosophila* LGR3. Secondly, a clade that includes *Drosophila* LGR4 (Fig. 3). Importantly, starfish receptors are positioned in both of these clades, with one protein positioned in the same clade as mammalian RXFP1/RXFP2 receptors and *Drosophila* LGR3, and the other protein positioned in the same clade as a *Drosophila* LGR4. Accordingly, receptors from other echinoderms and some protostome taxa are likewise positioned in both clades. Therefore, informed by this phylogenetic analysis we refer to the RXFP/LGR3-type receptor in *A. *cf.* solaris* and *A. rubens* as AsolRXFP/LGR3 and ArubRXFP/LGR3, respectively, and we refer to the LGR4-type receptor in *A. *cf.* solaris* and *A. rubens* as AsolLGR4 and ArubLGR4, respectively. Orthologs of these proteins identified in other echinoderms are named accordingly.

**Fig. 4:**
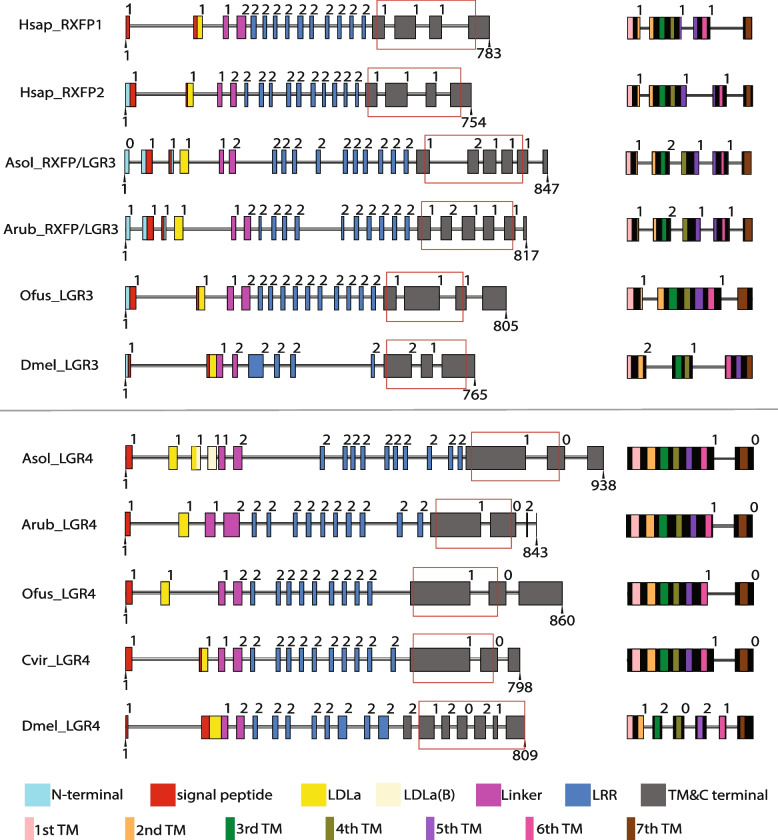
Comparison of the exon/intron structure of genes encoding RXFP/LGR3-type receptors and LGR4-type receptors in selected bilaterian taxa. Exons are shown as colored boxes, introns are shown as horizontal black and white lines, numbers above the colored boxes show the intron phase and numbers below the colored boxes show the positions of the first and last amino acid residues of each protein. The red rectangles show regions of the genes that encode the predicted seven transmembrane domains of each receptor and the more detailed diagrams on the right show the positions of introns with respect to the seven transmembrane domains. A phase 1 intron interrupting the coding sequence for TM domain 2 (orange) and a phase 1 intron interrupting the coding sequence for TM domain 5 (purple) are unique features of some genes encoding RXFP/LGR3-type receptors that distinguish these genes from those encoding LGR4-type receptors. Conversely, a phase 0 intron located after the exon that encodes the TM domain 7 (brown) is a unique feature of some genes encoding LGR4-type receptors that distinguish these genes from those encoding RXFP/LGR3-type receptors. The key shows the color coding for different regions/domains of the receptors. Species names are as follows: Hsap (*H. sapiens*), Asol (*A. *cf.* solaris*), Arub (*A. rubens*), Ofus (*O. fusiformis*), Dmel (*D. melanogaster*), Cvir (*C. virginica*). See detailed sequence analysis and positions of the introns in Additional file 15; Dataset S8

To further investigate relationships between the starfish RXFP/LGR3-type and LGR4-type receptors and leucine-rich repeat type C1 GPCRs in other taxa, we also analyzed and compared the structure of the genes encoding these proteins (Fig. 4). This revealed the presence/absence of evolutionarily conserved introns that distinguish genes encoding RXFP/LGR3-type proteins and genes encoding LGR4-type proteins. Thus, in genes encoding RXFP/LGR3-type proteins in humans (HsapRXFP1 and HsapRXFP2), starfish (AsolRXFP/LGR3; ArubRXFP/LGR3) and the annelid O. *fusiformis* (OfusLGR3) there is a conserved phase 1 intron that interrupts the coding sequence for the second transmembrane domain, whilst this intron is not present in LGR4-type genes. Furthermore, in genes encoding RXFP/LGR3-type proteins in humans (HsapRXFP1-2) and starfish (AsolRXFP/LGR3; ArubRXFP/LGR3) there is also a conserved phase 1 intron that interrupts the coding sequence for the fifth transmembrane domain, whilst this intron is not present in LGR4-type genes. Conversely, in genes encoding LGR4-type proteins in starfish (AsolLGR4; ArubLGR4), the annelid O. *fusiformis* (OfusLGR4) and in the mollusk *Crassostrea virginica* (CvirLGR) there is a conserved phase 0 intron located after the exon that encodes the seventh transmembrane domain, whilst this intron is not present in RXFP/LGR3-type genes. These findings provide further evidence that AsolRXFP/LGR3 and ArubRXFP/LGR3 are orthologs of the RXFP/LGR3-type proteins in other taxa and AsolLGR4 and ArubLGR4 are orthologs of LGR4-type proteins in other taxa. Furthermore, these findings, together with our phylogenetic analysis of sequence data, indicate that RXFP/LGR3-type genes and LGR4-type genes are paralogs that evolved via an evolutionarily ancient gene duplication in a common ancestor of the Bilateria*.*

In conclusion, informed by our comparative and phylogenetic analysis of candidate GPCRs for relaxin/insulin-type peptides in starfish, we hypothesized that AsolRXFP/LGR3 and ArubRXFP/LGR3 are receptors for both RGP1 and RGP2 in *A. *cf.* solaris* and *A. rubens*, respectively. Alternative hypotheses that are not supported by our phylogenetic analysis of gene/protein relationships are that (i) one peptide acts as a ligand for the RXFP/LGR3-type receptor and the other peptide acts as a ligand for the LGR4-type receptor, (ii) both peptides act as ligands for the LGR4-type receptor, but not the RXFP/LGR3-type receptor, and (iii) both peptides act as ligands for both the RXFP/LGR3-type receptor and the LGR4-type receptor.

### Comparative analysis of the structural characteristics of RXFP/LGR3-type and LGR4-type proteins in starfish and other taxa

As a prelude to experimental testing of RXFP/LGR3 and LGR4 as candidate receptors for RGP1 and/or RGP2 in starfish, we analyzed and compared the structural properties of these receptors and compared them to related receptors in other taxa. In *A. *cf.* solaris* there are two predicted isoforms of RXFP/LGR3 (isoform X1 = XP_022084824.1; 847 residues; isoform X2 = XP_022084826.1; 833 residues), with an alternative start codon conferring an additional 14 residues at the N-terminus in isoform X1. However, contained within both isoforms is a 775-residue sequence that has a predicted N-terminal signal peptide. In *A. rubens* there are also two predicted isoforms of RXFP/LGR3 (isoform X1 = XP_033631987.1; 836 residues; isoform X2 = XP_033631990.1; 817 residues), but with alternative splicing predicted to give rise to transcripts encoding proteins that differ in their C-terminal regions, but which are otherwise identical. Interestingly, ArubRXFP/LGR3 differs from AsolRXFP/LGR3 in not having a predicted N-terminal signal peptide. However, analysis of the N-terminal region of ArubRXFP/LGR3 revealed the presence of a predicted membrane-spanning domain, and analysis of this region of the protein using SignalP revealed that it has the structural characteristics of a signal peptide. Therefore, unlike AsolRXFP/LGR3, which has a typical N-terminal signal peptide, we infer that the putative membrane spanning region proximal to the N-terminus in ArubRXFP/LGR3 may function as an internal signal peptide (Fig. 4; Additional file 4: Fig. S4). Discovery of these differences in the predicted structures of RXFP/LGR3-type proteins in *A. *cf.* solaris* and *A. rubens* informed the design of experiments to test these proteins as receptors for RGP1 and RGP2, as discussed below.

We also investigated the occurrence of a predicted N-terminal signal peptide in RXFP/LGR3-type receptors in other taxa. In accordance with findings for AsolRXFP/LGR3, human RXFP1 and RXFP2 and *D. melanogaster* LGR3 have a predicted N-terminal signal peptide. In contrast, and in accordance with findings for ArubRXFP/LGR3, an N-terminal signal peptide is not predicted to be present in O. *fusiformis* LGR3. Therefore, we examined the sequence of the N-terminal region of O. *fusiformis* LGR3 in more detail and, importantly, we discovered that consistent with our findings for ArubRXFP/LGR3, there is a predicted internal signal peptide in this receptor (Fig. 4).

Turning now to LGR4-type proteins in starfish, there are two predicted isoforms in *A. *cf.* solaris* (isoform X1 = XP_022100793.1, 938 residues; isoform X2 = XP_022100794.1 888 residues) and three predicted isoforms in *A. rubens* (isoform X1 = XP_033635728.1, 905 residues; isoform X2 = XP_033635729.1, 884 residues; isoform X3 = XP_033635731.1, 864 residues). All of these isoforms have a potential N-terminal signal peptide within their sequences (Additional file 4: Fig. S4), which is a feature that is also seen in the LGR4-type proteins in the insect *D. melanogaster*, the annelid O. *fusiformis* and the mollusk *C. virginica* (Fig. 4). However, alternative splicing is predicted to generate isoforms of LGR4 in both *A. *cf.* solaris* and *A. rubens* that differ in their C-terminal regions. These findings informed the design of experiments to test these proteins as receptors for RGP1 and RGP2, as discussed below.

Lastly, a characteristic feature of leucine-rich repeat-type GPCRs is, as reflected in their name, the presence of multiple leucine-rich sequences in their extracellular N-terminal region (Fig. 4). However, a distinctive feature of the extracellular N-terminal region of type C leucine-rich repeat GPCRs, which is not present in type A and type B receptors, is a low-density lipoprotein receptor class A (LDLa) module. Accordingly, RXFP/LGR3 and LGR4 in the starfish *A. *cf.* solaris* and *A. rubens* have one or two LDLa modules in their extracellular N-terminal region (Fig. 4; Additional file 4: Fig. S4).

### RGP1 and RGP2 act as ligands for starfish RXFP/LGR3-type receptors

Informed by our analysis of the predicted sequences of RXFP/LGR3-type and LGR4-type proteins in *A. *cf.* solaris* and *A. rubens*, we then experimentally tested RGP1 and RGP2 as ligands for these receptors. The *A. *cf.* solaris* receptors were tested in the laboratory of R. Bathgate at the Florey Institute (Australia) and the *A. rubens* receptors were tested in the laboratory of M. Elphick at Queen Mary University of London (UK), as described below.

#### *A. *cf.* solaris*

N-terminally modified forms of AsolRXFP/LGR3 and AsolLGR4 were tested, incorporating the human prolactin signal peptide followed by a FLAG tag at the N-terminus. Furthermore, two isoforms of AsolLGR4 that differ in the sequences of their C-terminal regions were tested, which are referred to here as AsolLGR4(short) and AsolLGR4(long). Interestingly, all three cloned *A. *cf.* solaris* receptors demonstrated markedly higher cell surface expression in HEK-293T cells compared to human RXFP1 (Additional file 5: Fig. S5). Thus, cell surface expression of AsolRXFP/LGR3 was ~ 75-fold higher than human RXFP1 and cell surface expression of AsolLGR4(short) and AsolLGR4(long) was ~ 20-fold higher than human RXFP1. However, the total expression of the *A. *cf.* solaris* receptors in cells was only about twofold higher than human RXFP1. As the *A. *cf.* solaris* receptors were clearly expressed at the cell surface in HEK-293T cells, they were then tested for their ability to be activated by *A. *cf.* solaris* RGP1 and RGP2. Initial assays demonstrated very high cAMP activity for AsolRXFP/LGR3 when expressed in HEK-293T cells in the absence of peptides, whereas the basal cAMP activity was very low in cells transfected with AsolLGR4(short) and AsolLGR4(long), similar to human RXFP1 (data not shown). The AsolRXFP/LGR3 construct was therefore transfected at different plasmid amounts to assess if AsolRXFP/LGR3 exhibits constitutive activity. As shown in Additional file 5; Fig. S5B, the cAMP activity in HEK-293T cells was greater than the 5 µM forskolin response at high plasmid concentrations but decreased as the plasmid amount was reduced. This demonstrated that there was clear constitutive activity of AsolRXFP/LGR3 in HEK-293T cells. Stable cell lines expressing AsolRXFP/LGR3 were then generated with lower cell surface expression to enable consistent testing of *A. *cf.* solaris* RGP1 and RGP2 as ligands. CHO-AsolRXFP/LGR3 cells demonstrated lower constitutive activity than HEK-AsolRXFP/LGR3 cells (data not shown), and therefore CHO-AsolRXFP/LGR3 cells were used for testing *A. *cf.* solaris* RGP1 and RGP2 as ligands.

**Fig. 5:**
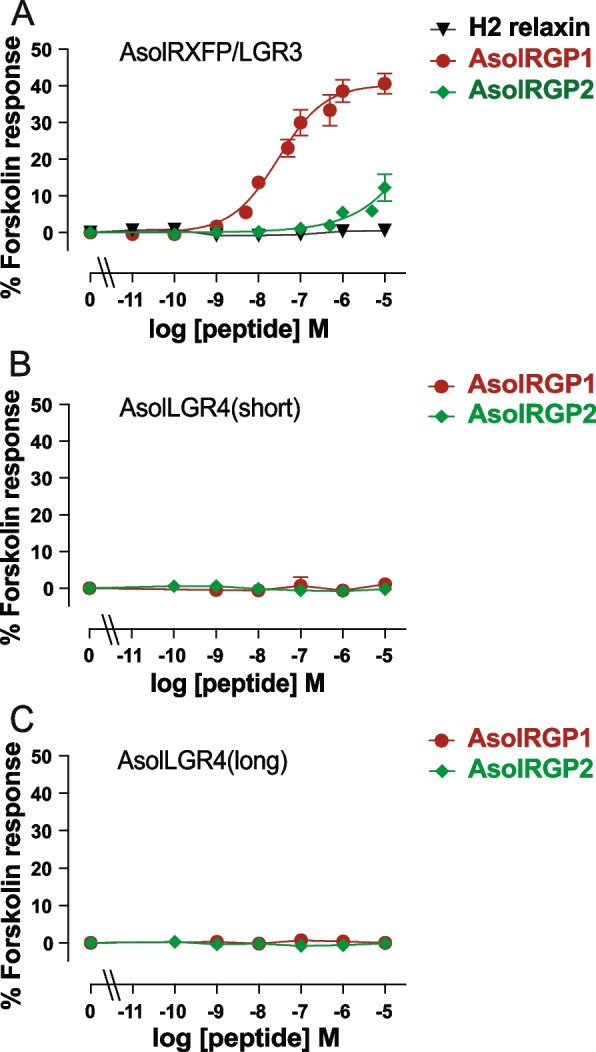
A. cf.* solaris* RGP1 is a ligand for *A. *cf.* solaris* RXFP/LGR3 but not for *A. *cf.* solaris* LGR4. **A** AsolRGP1 induces a dose-dependent increase in CRE reporter gene activation in CHO-AsolRXFP/LGR3 cells. The potency of AsolRGP1 at AsolRXFP/LGR3 was ~ 16 nM (pEC_50_ = 7.80 ± 0.18). AsolRGP2 induced a small but significant response but no clear dose–response, while the negative control H2 relaxin induced no response. **B**,** C** Neither AsolRGP1 nor AsolRGP2 induced CRE reporter gene activation in HEK-293 T cells transfected with AsolLGR4 (long) or AsolLGR4 (short). The graphs show the pooled data from at least three assays performed in triplicate within each assay (*n* ≥ 3) expressed as mean values with error bars (S.E.M.). The raw data from all three experiments are shown in Additional file 17: Dataset S10

HEK-293T cells transfected with AsolLGR4 (long) or AsolLGR4 (short) and a CRE reporter and CHO-AsolRXFP/LGR3 cells transfected with a CRE reporter were tested for their ability to respond to increasing concentrations of *A. *cf.* solaris* RGP1 or RGP2 (AsolRGP1 or AsolRGP2), with human H2 relaxin also tested. Concentration–response curves are shown in Fig. 5, which reveal that AsolRGP1 causes concentration-dependent cAMP activity in cells expressing AsolRXFP/LGR3 (Fig. 5A) but not in cells in expressing AsolLGR4(short) (Fig. 5B) or AsolLGR4(long) (Fig. 5C). AsolRGP2 also triggered cAMP activity in cells expressing AsolRXFP/LGR3 but only at high concentrations (Fig. 5A) and this peptide had no effect on cAMP activity in cells expressing AsolLGR4(short) (Fig. 5B) or AsolLGR4(long) (Fig. 5C). No response to H2 relaxin was observed in cells expressing AsolRXFP/LGR3, demonstrating the specificity of the effects of AsolRGP1 and AsolRGP2 (Fig. 5A). The responses to AsolRGP1 of cells expressing AsolRXFP/LGR3 were fitted to dose–response curves to generate potency (pEC_50_ = 7.74 ± 0.11, *n* = 6) and efficacy data (*E*_max_ = 38.6 ± 6.9% forskolin response). Dose–response curves could not be fitted for the data obtained from experiments testing AsolRGP2 on cells expressing AsolRXFP/LGR3. However, the percentage of forskolin-induced cAMP levels at 10 µM AsolRGP2 (12.2 ± 9.0% forskolin response, *n* = 6), was significantly greater than the background (*p* < 0.01; *t*-test), indicating that AsolRGP2 can act as a ligand for AsolRXFP/LGR3 in vitro, albeit at relatively high concentrations. In contrast, there was no significant increase in cAMP levels in HEK cells expressing AsolLGR4(short) or AsolLGR4(long) in response to any concentration of AsolRGP1 or AsolRGP2 tested.

#### *A. rubens*

**Fig. 6:**
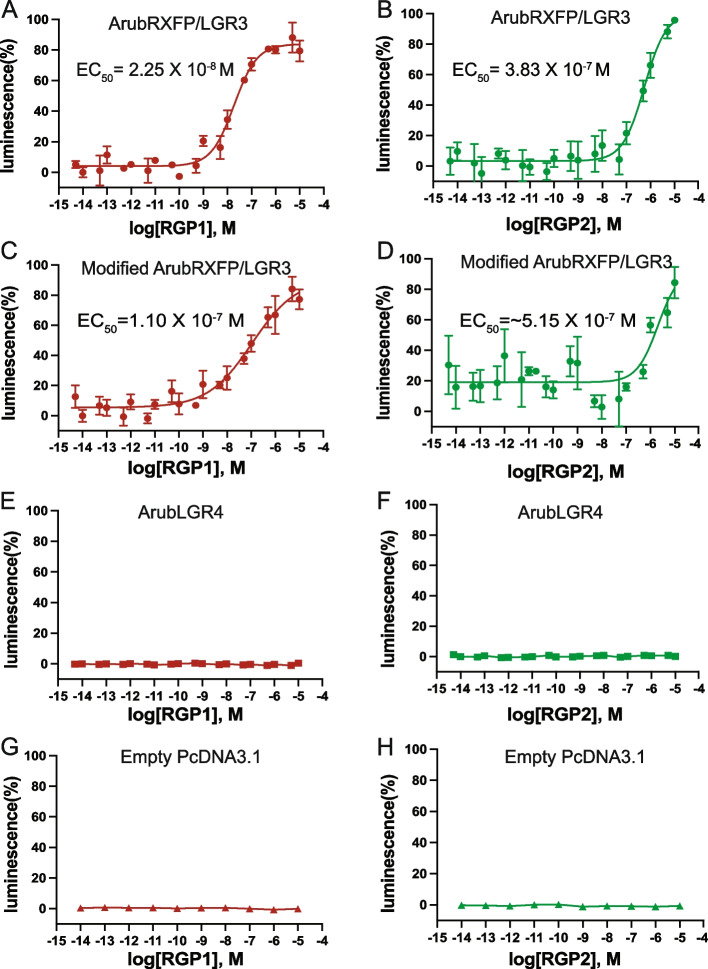
*A. rubens* RGP1 and RGP2 act as a ligand for *A. rubens* RXFP/LGR3, but not for *A. rubens* LGR4. **A**,** B** ArubRGP1 and ArubRGP2 trigger dose-dependent luminescence in CHO-K1 cells transfected with ArubRXFP/LGR3 and the chimeric G-protein GqS5 and stably expressing the calcium-sensitive luminescent GFP-apoaequorin fusion protein G5A. The EC_50_ values for ArubRGP1 and ArubRGP2 are 2.5 × 10^−8^ M and 3.8 × 10^−7^ M, respectively. **C**,** D** ArubRGP1 and ArubRGP2 trigger dose-dependent luminescence in CHO-K1 cells transfected with a modified form of ArubRXFP/LGR3 containing a mammalian N-terminal signal peptide (from the prolactin precursor) and the chimeric G-protein GqS5 and stably expressing the calcium-sensitive luminescent GFP-apoaequorin fusion protein G5A. The EC_50_ values for ArubRGP1 and ArubRGP2 are 1.1 × 10^−7^ M and ~ 5.1 × 10.^−7^ M, respectively. **E**,** F** ArubRGP1 and ArubRGP2 do not trigger luminescence in CHO-K1 cells transfected with ArubLGR4 and the chimeric G-protein GqS5 and stably expressing the calcium-sensitive luminescent GFP-apoaequorin fusion protein G5A. **G**,** H** ArubRGP1 and ArubRGP2 do not trigger luminescence in CHO-K1 cells transfected with an empty pcDNA3.1 plasmid, the chimeric G-protein GqS5 and stably expressing the calcium-sensitive luminescent GFP-apoaequorin fusion protein G5A. The absence of ArubRGP1 and ArubRGP2 induced luminescence in **E**–**H** demonstrates that the ArubRGP1 and ArubRGP2 induced luminescence in **A**–**D** can be specifically attributed to transfection of cells with ArubRFXP/LGR3 or modified ArubRFXP/LGR3. The graphs show results from a single experiment with each point showing mean values (*n* = 3) with error bars (S.E.M.). The data shown are representative of three independent experiments and the raw data from all three experiments are shown in Additional file 19: Dataset S12

ArubRGP1 and ArubRGP2 were tested as ligands for ArubRXFP/LGR3 (isoform X2; XP_033631990.1) and ArubLGR4 (isoform X3; XP_033635731.1) by co-transfection of CHO-K1 cells stably expressing the green fluorescent protein (GFP) – apoaequorin construct G5A [25] with the chimeric G-protein Gqs5, which is a G_q_-type protein modified to incorporate G_s_-type sequences that enable it to interact with GPCRs that preferentially couple to G_s_-type G-proteins [26]. Synthetic ArubRGP1 or ArubRGP2 were initially tested at a concentration of 10^−5^ M, comparing with luminescence measured in transfected cells incubated in basal media without the addition of ArubRGP1 or ArubRGP2. These experiments revealed that both ArubRGP1 and ArubRGP2 at a concentration of 10^−5^ M trigger a luminescence response (defined as 100%) in cells transfected with ArubRXFP/LGR3 that was approximately twice the background luminescence detected with the basal media used to dissolve the peptides. However, in cells transfected with ArubLGR4, luminescence measured in the presence of 10^−5^ M ArubRGP1 or ArubRGP2 was not significantly different to luminescence measured without the addition of these peptides to basal media (Additional file 6: Fig. S6). To examine the concentration-dependence of effects observed with ArubRGP1 and ArubRGP2, both peptides were tested at concentrations ranging from 10^−14^ to 10^−5^ M. These experiments revealed that the EC_50_ values for ArubRGP1- and ArubRGP2-induced luminescence in cells transfected with ArubRXFP/LGR3 were 2.25 × 10^–8^ M and 3.83 × 10^−7^ M, respectively (Fig. 6A, B). Importantly, no response to ArubRGP1 or ArubRGP2 was observed in CHO-K1 cells transfected with ArubLGR4 (Fig. 6E, F) or with an empty pcDNA3.1 + vector (Fig. 6G, H), demonstrating that ArubRGP1- and ArubRGP2-induced luminescence observed in CHO-K1 cells transfected with ArubRXFP/LGR3 can be attributed to activation of the transfected receptors and not activation of receptors endogenously expressed in CHO-K1 cells.

Because analysis of the sequence of ArubRXFP/LGR3 revealed the presence of a predicted internal signal peptide, we also synthesized and tested a modified form of ArubRXFP/LGR3 in which the N-terminal region of ArubRXFP/LGR3, including the predicted internal signal peptide, was replaced with the signal peptide sequence of the human prolactin precursor, which we refer to as modified ArubRXFP/LGR3. Thus, modified ArubRXFP/LGR3 was similar in this respect to the modified AsolRXFP/LGR3 protein (Fig. 5). These experiments revealed that both ArubRGP1 and ArubRGP2 act as ligands for modified ArubRXFP/LGR3 and the EC_50_ values for ArubRGP1- and ArubRGP2-induced luminescence in cells transfected with modified ArubRXFP/LGR3 were 1.1 × 10^−7^ M and ~ 5.1 × 10^−7^ M, respectively (Fig. 6C, D). These EC_50_ values are actually higher than those observed with unmodified ArubRXFP/LGR3 and therefore we concluded that the presence of the predicted internal signal peptide in ArubRXFP/LGR3 does not adversely affect its expression in CHO-K1 cells.

### Gonadotropic activity of AsolRGP1 and AsolRGP2 in *A. *cf.* solaris*

Although both RGP1 and RGP2 act as ligands for RXFP/LGR3 derived from *A. rubens* and *A. *cf.* solaris*, the potency/efficacy of RGP2 as a ligand for AsolRXFP/LGR3 was found to be much lower than that of RGP1 (Fig. 5). Therefore, it was of interest to investigate if RGP1 and RGP2 induce shedding of mature eggs from ovaries of *A. *cf.* solaris* when tested in vitro*.* Both RGP1 and RGP2 exhibited gonadotropic activity; furthermore, based on a semi-quantitative method for determination of potency, RGP2 appears be slightly less potent than RGP1 in inducing shedding of mature eggs from ovaries of *A. *cf.* solaris* when tested in vitro (Additional file 7: Fig. S7; Additional file 8: Dataset S1). We also tested a modified form of AsolRGP2 with the N-terminal glutamine residue of the A-chain converted to pyroglutamate. However, this peptide (Asol-pQ-RGP) was less potent than unmodified AsolRGP2 (Additional file 7: Fig. S7; Additional file 8: Dataset S1).

## Discussion

Fifty years after the discovery of a gonad-stimulating substance (GSS) that triggers spawning in starfish [17, 27], GSS was purified and identified as a relaxin-type neuropeptide and renamed relaxin-like gonad-stimulating peptide (RGP) [16, 18, 19]. Furthermore, a relaxin-type peptide has also been shown to exhibit gonadotropic activity in sea cucumbers [28], indicating that this is an ancient and conserved role of relaxin-type peptides in the phylum Echinodermata. Interestingly, analysis of transcriptome sequence data has revealed the occurrence of a second relaxin-like peptide (RLP2) in starfish [22] and recently it was reported that RLP2 also triggers spawning in the starfish species *P. pectinifera* and *A. rubens*. Therefore, RGP and RLP2 are now referred to as RGP1 and RGP2 (Mita et al., 2023). Accordingly, in this study, we show that both RGP1 and RGP2 trigger the shedding of mature eggs from the ovaries of the starfish *A. *cf.* solaris* when tested in vitro. Informed by these findings, the aims of this study were to investigate relationships of RGP1 and RGP2 with relaxin/insulin-type peptides in other taxa to gain insights into the evolution of these gonadotropic peptides and to identify the receptor or receptors that mediate their effects in starfish.

### Identification of RXFP/LGR3 as a receptor for RGP1 and RGP2 in starfish

Phylogenetic and cluster analysis of sequence data revealed that RGP1 and RGP2 belong to the family of relaxin-type peptides that include vertebrate relaxins, relaxin-like peptides in other echinoderms, and relaxin-like peptides in several protostome taxa, including the *Drosophila* peptide Dilp8. Therefore, it can be inferred that the evolutionary history of relaxin-type peptides can be traced back to an urbilaterian common ancestor of protostomes and deuterostomes. Closely related to the relaxin-type family of peptides is the insulin-like peptide 7 family (*Drosophila* Dilp7-type), which is found in several protostome taxa, echinoderms, and hemichordates, but not in chordates. Hence, it can be inferred that the origin of this family can also be traced back to an urbilaterian common ancestor of protostomes and deuterostomes, but with loss in chordates. Consistent with phylogenetic analysis, relaxin/Dilp8-type precursor genes and Dilp7-type precursor genes can be distinguished based on their exon/intron structure. Thus, whilst genes encoding both precursor types have a conserved phase 1 intron that interrupts the coding sequence of the C-chain domain, genes encoding Dilp7-type precursors also have a conserved phase 2 intron between the signal peptide and B-chain encoding regions of the genes. Furthermore, the occurrence of two or more genes encoding relaxin-type peptides in starfish and other taxa indicates that lineage-specific gene duplication has given rise to these paralogs. However, precise determination of the timing of these gene duplications will require analysis of sequence data from a wider range of taxa than currently available.

Identification of starfish RGP1 and RGP2 as relaxin/Dilp8-type peptides provided a basis for the identification of candidate receptor(s) for these peptides. Relaxin-type peptides in vertebrates exert their effects by binding to two distinctly different families of GPCRs. Firstly, RXFP1/2-type receptors, which are leucine-rich repeat type GPCRs, and secondly RXFP3/4-type receptors, which belong to a different family of GPCRs [5, 29]. Analysis of starfish transcriptome/genome sequence data revealed the presence of genes encoding homologs of RXFP1/2-type receptors, but not RXFP3/4-type receptors. Furthermore, phylogenetic analysis revealed that starfish and other echinoderms have two leucine-rich repeat-type GPCRs that are closely related to vertebrate RXFP1/2-type receptors. Firstly, a GPCR that is an ortholog of vertebrate RXFP1/2-type receptors and the *Drosophila* leucine-rich repeat type GPCR known as LGR3 and therefore we refer to this receptor as RXFP/LGR3 (i.e., AsolRXFP/LGR3 in *A. *cf.* solaris* and ArubRXFP/LGR3 in *A. rubens*). Secondly, a receptor that is an ortholog of the *Drosophila* leucine-rich repeat type GPCR known as LGR4 and which we therefore refer to as LGR4 (i.e., AsolLGR4 in *A. *cf.* solaris* and ArubLGR4 in *A. rubens*).

Importantly, consistent with the orthology of vertebrate RXFP1/2-type receptors and *Drosophila* LGR3, experimental studies have demonstrated that the *Drosophila* insulin-like peptide Dilp8 acts as a ligand for LGR3 [8]. This suggests that relaxin/Dilp8-type peptides have co-evolved with cognate RXFP/LGR3-type receptors. It is noteworthy that our analysis of the phylogenetic distribution of relaxin/insulin/IGF-type precursors revealed that a clade containing precursors of starfish RGP1, starfish RGP2, vertebrate relaxins, and *Drosophila* Dilp8 also contains precursors of relaxin/insulin-like peptides from taxa in which we have not found RXFP/LGR3-type receptors, including nematodes, mollusks, and platyhelminthes. However, some of the precursors in the clade containing precursors of starfish RGP1, starfish RGP2, vertebrate relaxins, and *Drosophila* Dilp8 have long branches leading to them. Therefore, sequence divergence/convergence in these precursor proteins may explain this mismatch. With regard to LGR4-type receptors, indirect evidence that Dilp7 acts as a ligand for LGR4 in *Drosophila* has also been reported [15]. Accordingly, our analysis of sequence data reveals good correspondence in the phylogenetic distribution of Dilp7-type precursors and LGR4-type proteins. Thus, genes encoding Dilp7-type precursors and LGR4-type receptors were found in several taxa, including echinoderms, hemichordates, arthropods, mollusks, annelids, nemerteans, but genes encoding Dilp7-type precursors and LGR4-type receptors were not found in chordates, nematodes, and platyhelminthes.

Informed by experimental identification of relaxin/Dilp8-type peptides as ligands for RXFP/LGR3-type receptors in vertebrates and *Drosophila*, indirect experimental evidence that Dilp7 acts as a ligand for LGR4 in *Drosophila* and our analysis of the phylogenetic distribution of RXFP/LGR3-type receptors and LGR4-type receptors, we hypothesized that RGP1 and RGP2 may both act as ligands for the RXFP/LGR3-type receptor in starfish. It should be noted that alternative hypotheses regarding the receptors that mediate the effects of RGP-type peptides in echinoderms have been proposed previously. For example, it was proposed that RGP-type peptides may exert their effects by binding to a receptor tyrosine kinase-type protein [30]. However, this hypothesis runs contrary to experimental findings that have demonstrated that the effects of RGP1 in starfish are mediated via G-protein signaling [18]. It has also been reported that in the starfish *P. pectinifera*, RGP1 acts as a ligand for a GPCR [31] which, based on our phylogenetic analysis, is an LGR4-type receptor (see Fig. 3). Therefore, to help resolve these conflicting hypotheses/findings, here we performed experiments in which RGP1 and RGP2 were tested as ligands for both the RXFP/LGR3-type and LGR4-type receptors in starfish. Importantly, we discovered that RGP1 and RGP2 act as ligands for RXFP/LGR3 in two starfish species—*A.* cf. *solaris* and *A. rubens*. Furthermore, we also discovered that RGP1 and RGP2 do not act as ligands for LGR4 in these two starfish species. Thus, our findings are inconsistent with the previous study that reported that RGP1 acts as a ligand for *P. pectinifera* LGR4. How can this inconsistency be explained? An explanation may be found in differences in the experimental methods employed. For the experiments reported by Mita et al. (2020), Sfp cells were transfected using a baculovirus-based method where the open reading frame (ORF) of the human promiscuous Gαq16 protein was subcloned into the *Xba*I site of the pFastbacI plasmid and the *P. pectinifera* LGR4-type receptor ORF was cloned into the *Not*I/*Xba*I site of the Gαq16-ligated pFastbacI plasmid [31]. Thus, with this method a chimeric protein comprising the *P. pectinifera* LGR4-type receptor N-terminally and the alpha subunit of Gq16 C-terminally was expressed in Sfp cells. Incorporation of the alpha subunit of Gq16 at the C-terminus of the receptor may alter the structure of the receptor in a way that enables the binding of peptides that do not act as ligands for the receptor physiologically. Furthermore, because the predicted ligands for LGR4-type receptors are the relaxin/insulin-like Dilp7-type peptides, promiscuous binding of the structurally related peptide RGP1 may have been facilitated by expression in cells of the chimeric LGR4-Gαq16 proteins that do not occur naturally. Accordingly, it is noteworthy that activation of LGR4-Gαq16 mediated signaling was only observed with relatively high concentrations of RGP1 and consequently it was not possible to determine an EC_50_ for *P. pectinifera* RGP1 as a candidate ligand for *P. pectinifera* LGR4 [31]. Informed by the findings of our experiments in which starfish RXFP/LGR3 and LGR4 were tested without a C-terminally linked G-protein alpha subunit, we conclude that RXFP/LGR3, and not LGR4, acts as the receptor for RGP1 and RGP2 physiologically in starfish.

It is noteworthy that both RGP1 and RGP2 exhibited lower potency when tested as ligands for RXFP/LGR3 in this study than when tested for gonadotropic activity in *A.* cf. *solaris* (this study) and in *A. rubens* and *P. pectinifera* [24]. This may be a consequence of heterologous expression of starfish receptors in mammalian cells, where receptor coupling with G-proteins may be less effective than with native G-proteins in starfish tissue. Furthermore, when testing RGP1 and RGP2 as ligands for RXFP/LGR3 we also observed that RGP2 was less potent than RGP1, most notably with the *A.* cf. *solaris* receptor. The physiological significance of this finding remains to be determined because RGP1 and RGP2 appear to exhibit similar potency when tested for spawning activity in *A.* cf. *solaris* (this study) and in *A. rubens* and *P. pectinifera* [24]. To address this issue in the future it would be interesting to knock down RXFP/LGR3 expression in starfish ovaries to investigate if this partially or completely abolishes responsiveness to RGP1 and/or RGP2 as gonadotropic peptides.

### The RXFP/LGR3-type receptor in the starfish *A. rubens* and the annelid *O. fusiformis* have an internal signal peptide

Analysis of the sequences of RXFP/LGR3-type receptors in different taxa revealed differences with regard to the presence of an N-terminal signal peptide. Thus, the RXFP/LGR3-type receptors in *A. cf. solaris*, human (RXFP1 and RXFP2), and *D. melanogaster* have a methionine residue at the start of the predicted signal peptide and therefore it can be inferred that this residue is encoded by a start codon and the receptors have an N-terminal signal peptide. However, the predicted signal peptide sequence in *A. rubens* RXFP/LGR3 does not have an N-terminal methionine residue and therefore the receptor must have an internal signal peptide. Furthermore, this feature of RXFP/LGR3 in *A. rubens* is also seen in the annelid *O. fusiformis*.

The occurrence of internal signal peptides has also been reported in other proteins, including receptors. For example, the receptor tyrosine kinase known as Sevenless (Sev), which is required for the specification of R7-type photoreceptor cells in *Drosophila*, has a signal peptide located sixty residues after the N-terminal methionine. Furthermore, evidence that cleavage of the N-terminal sixty residues and the internal signal peptide occurs during biosynthesis of the mature Sev protein was obtained [32]. By way of comparison, the putative internal signal peptide in ArubRXFP/LGR3 is located sixty-five residues after the N-terminal methionine. Furthermore, we infer that cleavage of the N-terminal sixty-five residues and the internal signal peptide occurs during the biosynthesis of ArubRXFP/LGR3. Evidence in support of this hypothesis is provided by our observation that replacement of the N-terminal eighty-seven residues of ArubRXFP/LGR3 (including the putative internal signal peptide) with the signal peptide of the human prolactin precursor yields a protein that is functional as a receptor for RGP1 and RGP2. It remains to be determined why internal signal peptides have evolved in some proteins. Our discovery that RXFP/LGR3-type receptors have an N-terminal signal peptide in some taxa (*A. *cf.* solaris*, human, and *D. melanogaster*) and an internal signal peptide in other taxa (*A. rubens*, *O. fusiformis*) may provide a basis for further investigation of this issue.

### Evolution and comparative physiology of relaxin-type signaling as a regulator of reproductive processes

Ever since the seminal discovery of relaxin as a relaxant of the interpubic ligament in mammals [1], the relaxin family of peptides has been linked with reproductive physiology [5, 29]. Although subsequent studies have also revealed non-reproductive associated functions of relaxins in mammals [5, 29], it is reproductive physiology that relaxins are mostly strongly associated with. This notion has been strengthened by the discovery and functional characterization of relaxin-type peptides in invertebrates. Thus, it was the identification of RGP (RGP1) as a gonadotropic neuropeptide in starfish that provided the first evidence that relaxin-type peptides are evolutionarily ancient regulators of reproductive processes [16, 18]. Evidence that this conserved function also extends beyond deuterostomes to protostomes has been provided by functional characterization of the Dilp8-LGR3 signaling system in adult *Drosophila*. Dilp8 is expressed at high levels in the ovaries of adult female *Drosophila* and activation of LGR3-expressing neurons in the abdominal ganglion of females inhibits receptivity to male courtship and reduces female fecundity. Thus, it is hypothesized that the ovaries release Dilp8 to signal their physiological state via LGR3-expressing neurons, which then regulate female reproductive behavior accordingly [33]. Furthermore, detailed analysis of *Drosophila* ovaries has revealed that Dilp8 is expressed in follicle cells that surround mature eggs and knockdown of Dilp8 expression in follicle cells causes a reduction in egg-laying. Accordingly, it is proposed that the release of Dilp8 by follicle cells acts locally to trigger LGR3-mediated follicle cell rupture, whilst also acting systemically via LGR3-expressing abdominal motor neurons to stimulate oviduct contractions [14].

In the context of what has been discovered about the role of Dilp8-LGR3 signaling in regulating reproductive processes in *Drosophila*, it is of interest to make comparisons with the RGP1&2-RXFP/LGR3 signaling system that we have discovered here in starfish. Immunohistochemical analysis of RGP1 has revealed a widespread pattern of expression [21], which contrasts with the much more restricted expression of Dilp8 in *Drosophila* ovarian follicle cells [14]. However, it has been proposed that it is the gonoducts that are the physiological source of RGP1 that triggers spawning in starfish [21]. Furthermore, RGP1 exerts its gonadotropic effect in starfish by binding to receptors on ovarian follicle cells, which triggers 1-methyladenine-mediated germinal vesicle breakdown in oocytes and spawning [18]. Thus, it is proposed that relaxin-type signaling within the reproductive system of *Drosophila* and starfish stimulates egg-laying and oocyte spawning, respectively, providing evidence of an evolutionarily conserved role in the regulation of reproductive processes. Accordingly, in both male and female mammals the reproductive system is both a source and site of action of relaxin-type peptides [5, 29].

**Fig. 7:**
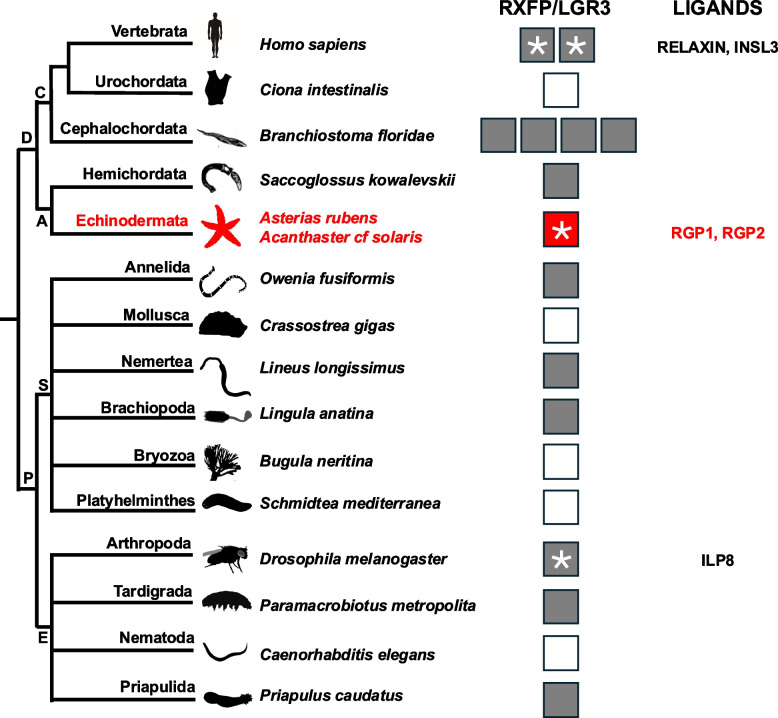
Diagram showing the phylogenetic distribution of RXFP/LGR3-type proteins in the Bilateria, with representative species from phyla/sub-phyla illustrated. Taxa in which genes encoding RXFP/LGR3-type proteins have not been found are shown with an empty square. Taxa in which genes encoding RXFP/LGR3-type proteins have been found but without experimental identification of a cognate ligand(s) are shown with a filled square without an asterisk. Taxa in which genes encoding RXFP/LGR3-type proteins have been found and with experimental identification of a cognate ligand(s) are shown with a filled square and an asterisk together with the name of the ligand(s). The identification of an RXFP/LGR3-type protein as the receptor for RGP1 and RGP2 in starfish (*A. rubens*, *A. cf solaris*), as reported in this study and highlighted here in red, is the first experimental characterization of a relaxin-type signaling system in a deuterostome invertebrate. The findings of this study indicate that the evolutionary origin of relaxin-RXFP1,2/LGR3-type signaling can be traced back to the common ancestor of the Bilateria, but with subsequent loss in some taxa. Abbreviations: D, Deuterostomia; P, Protostomia; C, Chordata; A, Ambulacraria; S, Spiralia; E, Ecdysozoa. Images of representative animals from each phylum/sub-phylum were obtained from http://phylopic.org or were created by the authors and their collaborators

Finally, it is of interest to reflect on the potential physiological significance of the phylogenetic distribution of relaxin/Dilp8 – RXFP/LGR3 type signaling. Previous experimental studies have identified relaxin/Dilp8-type peptides as ligands for RXFP/LGR3-type receptors in vertebrates and *Drosophila.* Our experimental identification of RXFP/LGR3-type proteins as receptors for RGP1 and RGP2 in starfish provides a missing link in the evolution of relaxin/Dilp8–RXFP/LGR3 type signaling, as illustrated in Fig. 7. Furthermore, our findings provide an exemplar for the experimental identification of ligands for RXFP/LGR3-type receptors in other taxa (Fig. 7) and a framework for further investigation of the comparative physiology of this signaling system. Thus, informed by our findings from starfish and previously reported findings from *Drosophila*, we speculate that in invertebrate taxa that have retained relaxin/Dilp8-RXFP/LGR3-type signaling (Fig. 7), this signaling system may participate in physiological mechanisms that regulate reproductive processes (e.g., spawning).

## Conclusions

We have identified a RXFP/LGR3-type G-protein coupled receptor that is activated by relaxin-like gonad-stimulating peptides in starfish. Functional characterization of this receptor provides a new insight into the evolution of relaxin-type signaling as a regulator of reproductive processes in bilaterian animals. Our detailed analysis of the phylogenetic distribution of relaxin-type peptides and RXFP/LGR3-type receptors provides a basis for functional characterization of relaxin-type signaling in other invertebrates, whilst also revealing evidence of loss of relaxin-type signaling in some invertebrate taxa.

## Methods

### Identifying relaxin/insulin-related peptide precursors and relaxin-type GPCRs in bilaterians using BLAST

To investigate the occurrence of relaxin/insulin-related peptide precursors in bilaterian taxa tBLASTn analysis [34] of the GenBank nucleotide sequence data was performed, using human relaxin-type peptide precursors as queries. In addition, locally installed BLAST was used for analysis of the genome sequence of the annelid *Owenia fusiformis* [35]. Because neuropeptide precursor proteins are relatively short (typically 100–200 residues) and often exhibit relatively low levels of sequence similarity between phyla, for these BLAST searches the cut-off for the *e*-value was set at 1000. Informed by results from BLAST analysis, we obtained datasets for two families of precursors that are closely related to human relaxin-type precursors. Firstly, a dataset comprising eighty-one sequences from deuterostomes and several insect species, including vertebrate relaxin-type precursors and the *Drosophila* Dilp8 precursor. Secondly, a dataset comprising the sequences of the *Drosophila* Dilp7 precursor and eighteen Dilp7-like precursors from other insects, ambulacrarians and mollusks. Each of these two datasets was aligned using Muscle 5.1 [36] using the PPP algorithm and the alignments were used to build models using HMMER 3.4 [37–39]. Then each model was used to search the proteome of thirty-one invertebrate species: *Priapulus caudatus* (phylum Priapulida); *Caenorhabditis elegans*,* Haemonchus contortus* (phylum Nematoda); *Hypsibius exemplaris*, *Paramacrobiotus metropolitanus* (phylum Tardigrada); *Limulus polyphemus*,* Centruroides sculpuratus*,* Bombus impatiens*,* Dascillus cervinus*,* Eueides isabella*,* Euschistus heros* (phylum Arthropoda); *Rotaria socialis (phylum Rotifera); Schmidtea mediterranea*,* Clonorchis sinensis* (phylum Platyhelminthes); *Pecten maximus*,* Mytilus edulis*,* Mactromeris polynyma*,* Aplysia californica*,* Gigantopelta aegis*,* Sepioteuthis lessoniana*,* Octopus bimaculoides* (phylum Mollusca); *Owenia fusiformis*,* Lamellibrachia columna*,* Oasisia *sp.,* Erpobdella octoculata*,* Lumbricus terrestris* (phylum Annelida); *Lineus longissimus* (phylum Nemertea), *Bugula neritina*,* Cryptosula pallasiana* (phylum Bryozoa); *Lingula anatina* (phylum Brachiopoda); and *Saccoglossus kowalevskii* (phylum Hemichordata). The proteomes of these species were obtained from the Ensembl Rapid Release (https://rapid.ensembl.org/info/about/species.html) and NCBI (https://www.ncbi.nlm.nih.gov/) archives and these are listed in Additional file 9: Dataset S2. All the sequences identified for each species within the inclusion threshold from the HMMER search were compiled in a single file and merged with the hit sequences from BLAST analysis to produce a single dataset. The sequences in this dataset were then filtered using the clustering algorithm CD-HIT (version 4.8.1) [40] set at 93% identity to avoid the inclusion of more than one protein isoform per gene. Then this dataset (Additional file 10: Dataset S3; Additional file 11: Dataset S4) was used to investigate protein relationships using CLuster ANalysis of Sequences (CLANS) [41] and phylogenetic methods (see below). Identification of homologous sequences was also facilitated by the highly conserved structure of relaxin/insulin-type peptide precursors, which comprise an N-terminal signal peptide followed sequentially by the B-chain, C-chain, and A-chain peptides, with the B- and A-chains followed or preceded, respectively, by cleavage sites. The closely related insulin-like growth factor (IGF) precursors have a similar structure, but with additional C-terminal D and E domains. Hit sequences were analyzed for the presence of a signal peptide using DeepLoc 2.0 and SignalP (both versions 3 and 6 were used) [42, 43]. Furthermore, sequences were analyzed manually for the presence of predicted monobasic/dibasic cleavage sites and conserved cysteine residues, which form interchain or intrachain disulfide bridges in mature relaxin/insulin-type peptides.

To identify relaxin-type receptors as candidate receptors for RGP1 and RGP2 in starfish, transcriptomic/genomic sequence data from two starfish species, *Acanthaster* cf. *solaris* and *Asterias rubens,* were analyzed by BLAST (https://blast.ncbi.nlm.nih.gov) using the sequences of the human relaxin receptors RXFP1–4 and the sequence of the *Drosophila* relaxin-type receptor LGR3 as queries (with default settings) and RXFP1,2/LGR3-related proteins were identified in both species. Then to enable a broader analysis of the relationship of these proteins with related GPCRs in other starfish and in other bilaterian taxa, tBLASTn analysis of GenBank nucleotide sequence data was performed to generate a database of sequences of proteins belonging to the leucine-rich repeat (LRR) containing family of GPCRs (LGRs). In addition, locally installed BLAST was used for analysis of the genome sequence of the annelid *Owenia fusiformis* [35]. Informed by results from the BLAST analysis, we obtained datasets for two families of receptors that are closely related to vertebrate RXFP1/2-type receptors. Firstly, a dataset comprising 22 sequences from deuterostomes, an annelid, and insects, including *Drosophila* LGR3. Secondly, a dataset comprising nine sequences from echinoderms, mollusks, and insects, including *Drosophila* LGR4. Each of these two datasets was aligned using Muscle 5.1 [36] using the PPP algorithm and the alignments were used to build models using HMMER 3.4 [37–39]. Then each model was used to search for these two receptor types in the proteome of thirty-one invertebrate species (Additional file 9: Dataset S2). Receptor sequences (both LGR3-type and LGR4-type) with a threshold < 1e^−50^ from the HMMER search for each species were compiled in a single file and then several steps were performed to filter the sequences. CLANS [41] with the scoring matrix BLOSUM62 and linkage clustering performed with an *e*-value of 1e^−40^ was used to identify coherent clusters, with the objective being to identify which of the sequences identified using HMMER cluster with sequences identified originally using BLAST. The sequences in this dataset were then filtered using the clustering algorithm CD-HIT (version 4.8.1) [40] set at 93% identity to avoid the inclusion of more than one protein isoform per gene. All sequences were analyzed for the presence of transmembrane domains (TMs) using Protter [44], Interpro domain predictions (EMBL-EBI), and TMHMM2.0c [45, 46], and only sequences with six, seven (as expected for GPCRs), or eight TMs were retained for subsequent analysis. Sequences were also analyzed for the presence and number of LRRs using Interpro domain predictions (EMBL-EBI) and regional annotation data available on NCBI GenBank. Furthermore, the presence of a low-density lipoprotein receptor class A (LDLa) domain, which is a structural characteristic of LGR-type relaxin-type receptors, was also investigated using Interpro domain predictions (EMBL-EBI) and regional annotation data available on NCBI GenBank. Lastly, the presence of a signal peptide was investigated using DeepLoc 2.0 and SignalP [42, 43]. Hit sequences that were identified as LGR-type receptors were compiled into a final sequence database (Additional file 12: Dataset S5; Additional file 13: Dataset S6) that was then used to investigate protein relationships using CLuster ANalysis of Sequences (CLANS) [41] and phylogenetic methods (see below).

### Investigation of the relationships of relaxin/insulin-related peptide precursors and relaxin receptor-related proteins in bilaterians using CLANS and phylogenetic methods

CLANS and phylogenetic methods were employed to investigate relationships of (i) relaxin/insulin-related peptide precursors in bilaterians, including starfish RGP1 and RGP2 precursors, and (ii) relaxin receptor-related proteins in starfish and other bilaterians. For CLANS [41], all-against-all BLAST was performed using the scoring matrix BLOSUM62, and linkage clustering was performed with an e-value of 1e^−2^ to identify the coherent clusters for precursors and an *e*-value of 1e^−40^ to identify coherent clusters for receptors. For phylogenetic analysis, multiple sequence alignments were generated using Muscle 5.1 [36] with the PPP algorithm. Maximum-likelihood trees were built using IQ-tree v 2.3.6 using the SH-aLRT and ultrafast bootstrap (UFBoot) methods with nearest neighbor interchange (NNI) correction tests [47–50]. For the precursor tree, the LG + R6 amino acid substitution model was selected, with a branch support bootstrap of 1000 replicates, with a clade comprising IGF/insulin/bombyxin-type precursors selected as an outgroup to root the tree. For the receptor trees, the LG + R10 amino acid substitution model was selected, with a branch support bootstrap of 1000 replicates, using glycoprotein hormone type receptors (clade A) and bursicon-type receptors (clade B) as an outgroup to root the tree. This research utilized Queen Mary’s Apocrita HPC facility, supported by QMUL Research-IT (http://doi.org/10.5281/zenodo.438045. The final figures for the phylogenetic trees were edited using Treeviewer version 2.2.0 [51].

### Alignment of sequences of the A- and B-chains of bilaterian relaxin-type peptides

Having identified relaxin-type precursors in a variety of bilaterian taxa, the predicted sequences of the A-chains and B-chains were aligned to identify specific similarities and differences in their sequences. This was accomplished using MAFFT7 (https://mafft.cbrc.jp/alignment/server/) with the iterative refinement method set to L-INS-I and the scoring matrix for amino acid sequences set to BLOSUM62, ensuring an optimal alignment. BOXSHADE (http://arete.ibb.waw.pl/PL/html/boxshade.html) was used to highlight residues that are identical or are conservative substitutions in at least 70% of the aligned sequences.

### Analysis of gene structure for relaxin/insulin-related peptide precursors and relaxin receptor-related proteins

To determine and compare the exon/intron structure of genes encoding (i) relaxin/insulin-related peptide precursors and (ii) relaxin receptor-related proteins, the sequences of transcripts/genes encoding these proteins were obtained from the NCBI GenBank database. Then the tool Splign (https://ncbi.github.io/magicblast/) was used to determine gene structures and IBS 1.0 was used to generate figures. The sequence data used and intron positions are summarized in Additional file 14: Dataset S7 and Additional file 15: Dataset S8.

### Pharmacological testing of RGP1 and RGP2 as candidate ligands for relaxin receptor-related proteins in the starfish *A.* cf. *solaris*

#### Synthesis of relaxin-type peptides

*A. *cf.* solaris* RGP1 and RGP2 peptides were synthesized as described previously [52] and recombinant human relaxin-2 (H2 relaxin) was obtained from Corthera Inc. (San Francisco, CA, a subsidiary of Novartis AG, Basel, Switzerland).

#### Cloning of cDNAs encoding *A. *cf.* solaris* relaxin receptor-related proteins into mammalian expression plasmids

Two genes encoding relaxin receptor-related proteins were identified in *A. *cf.* solaris* and, informed by phylogenetic analysis, these were named AsolRXFP/LGR3 and AsolLGR4. Furthermore, two isoforms of AsolLGR4 were identified, which we refer to as AsolLGR4(short) and AsolLGR4(long). The coding sequences for AsolRXFP/LGR3, AsolLGR4(short), and AsolLGR4(long) lacking a native N-terminal signal peptide were optimized for mammalian codon usage, but with the addition of a 5′ nucleotide sequence encoding an N-terminal bovine prolactin precursor signal peptide followed by a FLAG tag. The coding sequences for each construct are presented in Additional file 16: Dataset S9. Following the addition of a Kozak sequence (GCCACC) before the start codon of each coding sequence, the receptor cDNAs were synthesized in pUC57 vectors (GenScript, Hong Kong) with BamHI and XhoI restriction sites and Gateway‐compatible attB5 and attB2 sites at both ends to also allow recloning into pcDNA3.1/Zeo ( +) or Gateway (Invitrogen) vectors. pUC57 vectors were first digested with BamHI and XhoI restriction enzymes together with pcDNA3.1/Zeo ( +) followed by agarose gel purification of the digested DNA. Receptor constructs were ligated into the cut pcDNA3.1/Zeo ( +) vector to produce pcDNA-AsolRXFP/LGR3, pcDNA − AsolLGR4(short), and pcDNA − AsolLGR4(long). The sequences were confirmed by using Sanger sequencing on both strands. The AsolRXFP/LGR3 sequence was cloned into a lentiviral vector under the control of the constitutive human Ef1α promoter using Gateway technology (Invitrogen), according to the manufacturer’s instructions. PCR was used to amplify from the attB5 and attB2 sequences as described [53] and the PCR product was gel‐purified and cloned into the pDONR 221 P5–P2 vector containing an IRES-GFP using BP Clonase II. The resulting pENTR L5–L2 entry clone was confirmed to have the correct sequence by sequencing on both strands using Sanger sequencing. A pENTR L1–R5 Ef1α entry clone was then cloned along with the pENTR L5–L2 AsolRXFP/LGR3-IRES-GFP clone into the pLenti X1 Zeo DEST vector [54] using LR Clonase II, to generate the pLenti X1-Ef1α-AsolRXFP/LGR3-IRES-GFP expression clone.

#### Cell culture and transfection

Human embryonic kidney (HEK) 293T cells were grown in complete Dulbecco’s modified Eagle medium (DMEM; Gibco) and Chinese hamster ovary (CHO) cells in complete DMEM/F12 (Gibco). Media was supplemented with 10% fetal bovine serum, 1% penicillin/streptomycin, and 1% L‐glutamine (complete media; All from Gibco). HEK-293T cells were transfected with LipofectAMINE 2000 (Invitrogen) and CHO cells with CHO TransIT and CHO-Mojo (Mirus), both according to the manufacturer’s instructions.

#### Receptor expression assays

The total expression of the *A.* cf. *solaris* receptors in cells and at the cell surface was investigated in parallel with human RXFP1 in HEK-293T cells using an anti-FLAG ELISA method described previously [55]. Cells were transfected with either pcDNA3.1-AsolRXFP/LGR3, pcDNA3.1-AsolLGR4(short), or pcDNA3.1-AsolLGR4(long), pcDNA3.1-human RXFP1, or an empty pcDNA3.1 plasmid in triplicate within 24-well plates to assess cell surface and total receptor expression. After 24 h, cells were fixed using an assay buffer with 3.7% formaldehyde for cell surface expression or 3.7% formaldehyde/0.25% Triton-X for total cell expression. Subsequently, cells were blocked for 45 min in an assay buffer containing 1% BSA. Receptor expression was determined by incubating cells at room temperature for 1 h with 10 µg/mL mouse anti-FLAG M1 monoclonal antibody (Sigma-Aldrich), followed by washing and incubation with 2 µg/mL goat anti-mouse Alexa 488 conjugated antibody (Invitrogen). The cells were then lysed and transferred to black-walled 96-well optiplates for fluorescence measurement at 520 nm, with excitation at 479–491 nm, using a Polarstar Omega platereader (BMG Labtech). The empty pcDNA3.1-transfected cells served as controls to determine non-specific background fluorescence, and *A. *cf.* solaris* receptor expression was expressed as a percentage of human RXFP1 receptor expression. Data from four independent experiments are presented as mean ± SEM, normalized to human RXFP1 expression. Statistical analysis of expression differences was performed using paired t-tests on raw cell surface and total expression data with GraphPad PRISM 10.

#### Preparation of AsolRXFP/LGR3 stably expressing cell lines

CHO and HEK293T cells stably expressing AsolRXFP/LGR3 were generated by lentiviral transduction. HEK293T cells were plated in 10 cm dishes for transfection with pLenti X1-Ef1α-COT-RXFP-like 2-IRES-GFP along with the lentiviral packaging and envelope plasmids pMDL, pRSV‐Rev, and pCMV‐VSV‐G, using Lipofectamine 2000. The following day, media containing lentivirus was harvested and filtered through a 0.45 μm syringe filter. Fresh CHO and HEK293T cells in 10 cm plates were transduced by replacing the cell culture media with the filtered media containing lentivirus, mixed with 4 μg/mL hexadimethrine bromide (Polybrene) to increase transduction efficiency. Three rounds of transduction were performed over 32 h, before cells were allowed to recover for three days in complete media. Transduced cells were sorted from non-transduced cells using the GFP reporter by fluorescence-activated cell sorting (FACS), using a Becton Dickinson FACSAria III as previously described [53]. Single, live cells with low GFP expression were selected to represent low AsolRXFP/LGR3 expression and were subsequently grown to confluency in a T175 flask and cryopreserved.

#### CRE reporter gene assays

The ability of the *A. *cf.* solaris* receptors to respond to *A. *cf.* solaris* RGP1 and RGP2 peptides was tested using a pCRE β-galactosidase reporter gene assay in CHO cells stably expressing AsolRXFP/LGR3 or HEK-293T cells transfected with AsolRXFP/LGR4 (long) or AsolRXFP/LGR4 (short), as described in detail previously [56]. Importantly, the pCRE β-galactosidase reporter gene assay measures both Gs and Gq activation downstream of GPCR activation by monitoring CREB-mediated gene transcription [57]. Cells were seeded in 96 well plates and were left to grow overnight at 37 °C in an incubator with 5% CO_2_. The next day the cells were transfected with different amounts of the pCRE reporter gene DNA [57] and/or pcDNA3.1 receptor constructs, as described above, and were left to grow overnight in an incubator. Triplicate wells in each plate were transfected with the equivalent amount of pCRE reporter gene DNA and pcDNA3.1 without a receptor-encoding insert as the background CRE activity control. The next day cells were exposed in triplicate wells to increasing concentrations of peptides diluted in media for 6 h at 37 °C in an incubator. Triplicate parallel wells contained 5 µM forskolin and blank media as positive and negative controls, respectively. At the end of the incubation period, the media was aspirated, and the plate was stored at − 80 °C until development. Plates were thawed for the development of β-galactosidase activity, as described previously [56], with absorbance being read at 570 nM on an Epoch 2 microplate reader. The data were analyzed as the percentage of the response induced by 5 µM forskolin and pooled followed by fitting to a three-parameter sigmoidal dose–response curve using GraphPad Prism 10. Experiments were performed at least three times with triplicate determinations in each assay (Additional file 17: Dataset S10). The efficacy of AsolRGP2 compared to background cAMP activity in AsolRXFP/LGR3-expressing CHO cells was assessed using unpaired *t*-tests in Prism 10.

### Pharmacological testing of RGP1 and RGP2 as candidate ligands for relaxin receptor-related proteins in *A. rubens*

*A. rubens* RGP1 and RGP2 (ArubRGP1, ArubRGP2) were custom synthesized with a purity of > 95%, as reported previously [20, 24]. Two genes encoding relaxin receptor-related proteins were identified in *A. rubens* and, informed by phylogenetic analysis, these were named ArubRXFP/LGR3 and ArubLGR4. Then cDNAs encoding these proteins were custom synthesized with the inclusion of a 5′ partial Kozak translation initiation sequence (GCCACC) and incorporated into the mammalian expression vector pcDNA 3.1( +) (Genscript, Hong Kong) (Additional file 18: Dataset S11). Likewise, a cDNA encoding an N-terminally modified form of ArubRXFP/LGR3 incorporating an N-terminal signal peptide from the bovine prolactin precursor was also synthesized (with codon optimization for mammalian expression) (Genewiz, Takeley, UK) and this is referred to henceforth as ArubRXFP/LGR3(modified) (Additional file 18: Dataset S11). Chinese hamster ovary cells (CHO-K1) stably expressing the calcium-sensitive aequorin fusion protein G5A were used as an expression system. This cell line was supplied by Prof. Gaspar Jékely (University of Heidelberg, Germany) and tested negative for mycoplasma contamination using a MycoAlert PLUS kit (Lonza, Switzerland). The CHO-K1 cells were cultured in a culture medium comprising DMEM/F12, 10% fetal bovine serum, antibiotic–antimycotic, and 20 µg/mol Geneticin G418 sulfate (all purchased from Thermo Fisher scientific, Oxford, UK; cat no. 11039047, 10,082,147, 15,240,062, and 10,131,035, respectively.) When the cells reached 80% confluency, they were co-transfected with plasmids containing cDNAs encoding ArubRXFP/LGR3, ArubRXFP/LGR3(modified), or ArubLGR4 and a plasmid encoding the chimeric G-protein GqS5 (Addgene, Watertown USA), which enables GPCRs that preferentially couple to Gq-type or Gs-type proteins to signal via the phospholipase-C signaling pathway [26].

AruRGP1 and AruRGP2 were tested as ligands for the *A. rubens* receptors using luminescence-based assays, which have been described previously [58]. Thus, 48 h after transfection, cells were detached by using PBS buffer (pH7.4; Thermofisher Scientific, Oxford, UK; cat no.10010023) supplemented with a final concentration of 5 mM EDTA pH8.0 (Thermo Fisher Scientific, Oxford, UK; cat no.15575020). Cells were then washed with DMEM/F12 buffer and then incubated with 1 mM coelenterazine-H (Thermo Fisher Scientific, Oxford, UK; cat no. C6780) at room temperature with stirring for 3 h. ArubRGP1 or ArubRGP2 were prepared at concentrations ranging from 10^−14^ to 10^−5^ M and then were pipetted into 96-well plates (Costar Assay microplate, REF: 3903 or Falcon 96 well microplate, Code: 353,377). A FLUOstar Omega Plate Reader (BMG LABTECH; FLUOstar Omega Series multi-mode microplate reader) with injectors was used to sequentially add transfected CHO-K1 cells to each test well of the microtiter plate and luminescence was recorded over a 35-s period. Luminescence data were integrated over the 35 s measurement period and the mean of the triplicate measurements was calculated. Responses were normalized to the maximum response obtained in each experiment (100% activation) and to luminescence measured with the vehicle media (0% activation) (Additional file 19: Dataset S12). Dose–response curves were fitted with a four-parameter curve and EC_50_ values were calculated from dose–response curves based on at least three measurements from three independent transfections using Prism 9.0 (GraphPad, La Jolla, USA).

### Testing of AsoRGP1 and AsoRGP2 in in vitro spawning assays

Adult *A. *cf.* solaris* were collected from the Great Barrier Reef by Pacific Marine Group divers associated with the Great Barrier Reef Marine Park Authority (GBRMPA) before being transported to the University of the Sunshine Coast aquarium facility. Animals were briefly maintained in a re-circulating protein-skimmed and aerated saltwater system of approximately 1500 L. The animals used were approximately 25–55 cm in diameter and kept unfed for the duration of the experiment. The gender and ovarian maturation stage was determined by using a minor gonad tissue biopsy technique [59] and, thereafter, animals were individually segregated in a flowthrough aerated tank filled with approximately 55 L of filtered seawater for a minimum of 24 h to recover and acclimatize.

AsoRGP1 and AsoRGP2 were synthesized as described previously [52, 60]. In addition, a modified form of AsoRGP2 was synthesized in which the N-terminal residue of the A-chain (glutamine, Q) was substituted with pyroglutamate (pQ), recognizing the possibility of this potential post-translational modification. Hence, we refer to this peptide as Aso-pQ-RGP2. In vitro spawning assays using ovarian fragments were performed following procedures described previously [20]. In brief, the ovaries were excised and cut using scissors into small fragments containing only a few lobes. The ovarian fragments were then incubated in artificial seawater containing RGP-type peptides at a range of concentrations (2 × 10^−10^–2 × 10^−6^ M) for 1 h. As a control experiment, ovarian fragments were incubated in artificial seawater alone. The samples were examined to determine whether or not in vitro spawning had occurred and were scored [61] as follows: (+ + +) spawning occurred and most oocytes had matured; (+ +) about 50% of oocytes had matured, ( +) a few oocytes had matured and ( −): no spawning occurred. Scores were then converted to numerical values (+ + + = 100; + + = 67; + = 33; − = 0), so that the effective dose for induction of spawning in 50% of ovarian fragments (EC_50_) was determined graphically. Means ± standard error of mean (SEM) were determined from four separate assays using ovaries from four different animals.

## Supplementary Information

Additional file 1. Fig. S1. BLOSUM62 cluster map of relaxin/insulin/IGF-type peptide precursors showing that the *A. rubens* RGP1 and RGP2 (ArubRGP1, ArubRGP2) and the *A.* cf. *solaris* RGP1 and RGP2 (AsolRGP1, AsolRGP2) precursors are positioned in a cluster that contains vertebrate relaxin precursors. Nodes are labelled with phylum-specific colours, shown in the key, and connections represent BLAST relationships with a P value > 1e-1. Precursors are labelled with different symbols in accordance with clades they are positioned in the phylogenetic tree shown in Fig. 1 and taxa colour coded (see key). The *Asterias rubens* and *Acanthaster* cf. *solaris* RGP1 and RGP2 precursors are labelled. Accession numbers for precursor sequences included in this figure are listed in Additional file 10: Dataset S3 and the sequences of the precursor proteins in FASTA format are listed in Additional file 11: Dataset S4.

Additional file 2. Fig. S2. Alignment of the sequences of the B-chains (upper) and A-chains (lower) of relaxin-type peptides in bilaterian taxa. The aligned amino acids are highlighted in black if the residue is present in at least 70% of the sequences or highlighted in grey if conservative amino acid substitutions are present in at least 70% of the sequences. The conserved cysteine motifs are underlined with red stars and the position of putative disulphide bonds are shown in the alignment with red lines. Note that presence of an alanine residue in the B-chain (red) and glycine-isoleucine dipeptide sequence (red) in the A-chain that is conserved between starfish RGP2-type peptides and the *Drosophila* Dilp8-type peptide and/or the *O. fusiformis* (phylum Annelida) relaxin-like peptide. Species and peptide names are highlighted in taxon-specific colours: purple (Vertebrata), green (Cephalochordata), light blue (Echinodermata), orange (Annelida), red (Arthropoda). Species name abbreviations are as follows: Hsap (*H. sapiens*), Pmar (*P. marinus*), Bflo (*B. floridae*), Bbel (*B. belcheri*), Arub (*A. rubens*), Asol (*A.* cf. *solaris*), Mgla (*M. glacialis*), Pmin (*P. miniata*), Ovic (*O. victoriae*), Hsca (*H. scabra*), Amed (*A. mediterranea*), Ofus (*O. fusiformis*), Dmel (*D. melanogaster*). The accession numbers and sequences of the precursors of the relaxin-type peptides included in this figure are listed in Additional file 10: Dataset S3 and Additional file 11: Dataset S4, respectively.

Additional file 3. Fig. S3. BLOSUM62 cluster map showing that the *A. *cf. *solaris *and *A. rubens* G-protein coupled receptors AsolRXFP/LGR3, ArubRXFP/LGR3, AsolLGR4 and ArubLGR4 (boxed) are positioned in a cluster (type C1) that contains vertebrate RXFP1/RXFP2-type receptors and arthropod LGR3-type and LGR4-type receptors. Nodes are labelled with receptor-type specific symbols and phylum-specific colours, as shown in the key. Connections represent BLAST relationships with a P value > 1e-10. Accession numbers for receptor sequences included in this figure are listed in Additional file 12: Dataset S5 and the sequences of the receptor proteins in FASTA format are listed in Additional file 13: Dataset S6.

Additional file 4. Fig. S4. Predicted membrane topology of the *A.* cf. *solaris *G-protein coupled receptors AsolRXFP/LGR3 (A) and AsolLGR4 (B) and the *A. rubens* G-protein coupled receptors ArubRXFP/LGR3 (C) and ArubLGR4 (D). All four receptors have seven predicted transmembrane domains, as expected for G-protein coupled receptors. The predicted extracellular N-terminal region of the receptors contain several predicted glycosylation sites (green), a low-density lipoprotein receptor class A (LDLa, yellow) module and leucine-rich repeats (LRRs, blue), which are a characteristic feature of leucine-rich repeat type G-protein coupled receptors. However, a key difference between the receptors is the position of the predicted signal peptide (red), which in A. solRXFP/LGR3, AsolLGR4 and ArubLGR4 is located at the N-terminus of the protein, whilst in ArubRXFP/LGR3 it is located internally and with a valine residue located at the start of the predicted signal peptide. In silico analysis of the amino acid sequences of the four receptors was performed using Protter (https://wlab.ethz.ch/protter).

Additional file 5. Fig. S5. A. Analysis of the cell surface and total expression of *A.* cf *solaris* receptors AsolRXFP/LGR3, AsolLGR4(long) and AsolLGR4(short) compared to human RXFP1 (hRXFP1). B. Constitutive activity demonstrated by AsolRXFP/LGR3. pCRE reporter gene activity in the absence of ligand with different amounts of transfected receptor is shown. Data are expressed as the % forskolin activity. Assays were performed in triplicate within each assay and were repeated at least three times and are presented as mean values with error bars (S.E.M.).

Additional file 6. Fig. S6. Luminescence responses of CHO-K1 cells expressing ArubRXFP/LGR3 when exposed to BSA (control), ArubRGP1RGP (10–5 M) and ArubRG2 (10–5 M) 30 s. The data were analysed by one-way ANOVA with Bonferroni’s multiple comparisons post hoc test, revealing that luminescence responses in cells exposed to ArubRGP1 and ArubRGP2 are significantly higher (***p < 0.001, *p < 0.1) than in cells exposed to BSA media. The data shown are representative of three independent experiments.

Additional file 7. Fig. S7. AsolRGP1 and AsolRGP2 cause dose-dependent induction of spawning of ovarian fragments from *A*. sf. *solaris*. Asol-pQ-RGP2, a modified form of AsoRGP2 in which the N-terminal residue of the A-chain (glutamine, Q) was substituted with pyroglutamate (pQ), was also tested but was less potent than AsolRGP1 and AsolRGP2. + + + denotes spawning occurred and most of oocytes were matured, + + denotes about 50% oocytes were matured, + denotes a few oocytes were matured, and – denotes no spawning occurred. Symbols and bars represent the mean for four separate assays using ovaries from four different animals and standard error of the mean (SEM), respectively.

Additional file 8. Dataset S1. AsolRGP1 and AsolRGP2 cause dose-dependent induction of spawning of ovarian fragments from *A.* sf. *solaris*. Experimental data for the graphs are shown in Additional file 7: Fig. S7.

Additional file 9. Dataset S2. Details of proteomes analysed to identify precursors and receptors listed in Additional file 10: Dataset S3; Additional file 11: Dataset S4; Additional file 12: Dataset S5; Additional file 13: Dataset S6.

Additional file 10. Dataset S3. Accession numbers or references for precursor protein sequences used for the CLANS and phylogenetic tree shown in Additional file 1: Fig. S1 and Fig. 1, respectively

Additional file 11. Dataset S4. FASTA file containing the sequences of the precursor proteins used for the CLANS and phylogenetic tree shown in Additional file 1: Fig. S1 and Fig. 1, respectively.

Additional file 12. Dataset S5. Accession numbers for receptor sequences used for the CLANS and phylogenetic tree shown in Additional file 3: Fig. S3 and Fig. 3, respectively.

Additional file 13. Dataset S6. FASTA file containing the sequences of the receptors used for the CLANS and phylogenetic tree shown in Additional file 3: Fig. S3 and Fig. 3, respectively.

Additional file 14. Dataset S7. Sequences and accession numbers of the precursor proteins shown in Fig. 2, with the position and phase of introns in the corresponding gene indicated by black highlighting and numbers.

Additional file 15. Dataset S8. Sequences of the receptors shown in Fig. 4, showing the positions of different domains and with the position of introns in the corresponding gene indicated by black highlighting.

Additional file 16. Dataset S9. Sequence data for the *A.* cf *solaris* proteins tested as receptors for AsolRGP1 and AsolRGP2, as shown in Fig. 5.

Additional file 17. Dataset S10. Experimental data for the graphs shown in Fig. 5.

Additional file 18. Dataset S11. Sequence data for the *A. rubens* proteins tested as receptors for ArubRGP1 and ArubRGP2, as shown in Fig. 6.

Additional file 19. Dataset S12. Experimental data for the graphs shown in Fig. 6.

## Data Availability

All datasets generated or analysed during this study are included in this published article and its supplementary additional files.

## Abbreviations

ArubRGP1: *Asterias rubens* Relaxin-like gonad-stimulating peptide 1
ArubRGP2: *Asterias rubens* Relaxin-like gonad-stimulating peptide 2
ArubRXFP/LGR3: *Asterias rubens* Relaxin family peptide receptor/ leucine-rich repeat G protein-coupled receptor 3
ArubLGR4: *Asterias rubens* Leucine-rich repeat G protein-coupled receptor 4
Asol-pQ-RGP2: *Acanthaster *cf.* solaris* Relaxin-like gonad-stimulating peptide 2 modified with pyroglutamate replacing glutamine at the N-terminus of the A-chain
AsolRGP1: *Acanthaster *cf.* solaris* Relaxin-like gonad-stimulating peptide 1
AsolRGP2: *Acanthaster *cf.* solaris* Relaxin-like gonad-stimulating peptide 2
AsolRXFP/LGR3: *Acanthaster *cf.* solaris* Relaxin family peptide receptor/ leucine-rich repeat G protein-coupled receptor 3
AsoLGR4: *Acanthaster *cf.* solaris* Leucine-rich repeat G protein-coupled receptor 4
BLAST: Basic Local Alignment Search Tool
BLOSUM62: BLOcks SUbstitution Matrix built using sequences with less than 62% similarity
cAMP: Cyclic adenosine monophosphate
CD-HIT: Cluster Database at High Identity with Tolerance
cDNA: Complementary DNA
CHO: Chinese hamster ovary
CLANS: CLuster ANalysis of Sequences
CvirLGR4: *Crassostrea virginica* Leucine-rich repeat G protein-coupled receptor 4
Dilp1-6: Drosophila insulin-like peptides 1–6
Dilp7: Drosophila insulin-like peptide 7
Dilp8: Drosophila insulin-like peptide 8
EC_50_: Half maximal effective concentration
GPCR: G-protein coupled receptor
GPCR135: G-protein coupled receptor 135
GPCR142: G-protein coupled receptor 142
GSS: Gonad-stimulating substance
HEK: Human embryonic kidney
HPC: High Performance Compute cluster
HsapRXFP1: *Homo sapiens* Relaxin family peptide receptor 1
HsapRXFP2: *Homo sapiens* Relaxin family peptide receptor 2
IGF: Insulin-like growth factor
INSL3–6: Insulin-like peptides 3–6
LG: Le & Gascuel protein substitution model
LDL: Low-density lipoprotein
LGR3: Leucine-rich repeat G protein-coupled receptor 3
LGR4: Leucine-rich repeat G protein-coupled receptor 4
LRR: Leucine-rich repeat
NCBI: National Center for Biotechnology Information
NNI: Nearest neighbor interchange
OfusRLP: *Owenia fusiformis* Relaxin-like peptide
OfusLGR3: *Owenia fusiformis* Leucine-rich repeat G protein-coupled receptor 3
RGP1: Relaxin-like gonad-stimulating peptide 1
RGP2: Relaxin-like gonad-stimulating peptide 2
RLN1–3: Relaxins1–3
RXFP1–4: Relaxin family peptide receptors 1–4
RXFP/LGR3: Relaxin family peptide receptor/leucine-rich repeat G protein-coupled receptor 3
SH-aLRT: Shimodaira–Hasegawa approximate likelihood-ratio test
TMs: Transmembrane domains
UFBoot: Ultrafast bootstrap
