# Supplementary material for: Receptor deorphanization in starfish reveals the evolution of relaxin signaling as a regulator of reproduction
Source: BMC Biol. 2025 Feb 25;23:59. doi: 10.1186/s12915-025-02158-2 (PMC11863921; doi:10.1186/s12915-025-02158-2)
Supplement: Supplementary file 1 — Additional file 1. Fig. S1. BLOSUM62 cluster map of relaxin/insulin/IGF-type peptide precursors showing that the A. rubens RGP1 and RGP2 (ArubRGP1, ArubRGP2) and the A. cf. solaris RGP1 and RGP2 (AsolRGP1, AsolRGP2) precursors are positioned in a cluster that contains vertebrate relaxin precursors. Nodes are labelled with phylum-specific colours, shown in the key, and connections represent BLAST relationships with a P value > 1e-1. Precursors are labelled with different symbols in accordance with clades they are positioned in the phylogenetic tree shown in Fig. 1 and taxa colour coded (see key). The Asterias rubens and Acanthaster cf. solaris RGP1 and RGP2 precursors are labelled. Accession numbers for precursor sequences included in this figure are listed in Additional file 10: Dataset S3 and the sequences of the precursor proteins in FASTA format are listed in Additional file 11: Dataset S4. [file 12915_2025_2158_MOESM1_ESM.pdf]

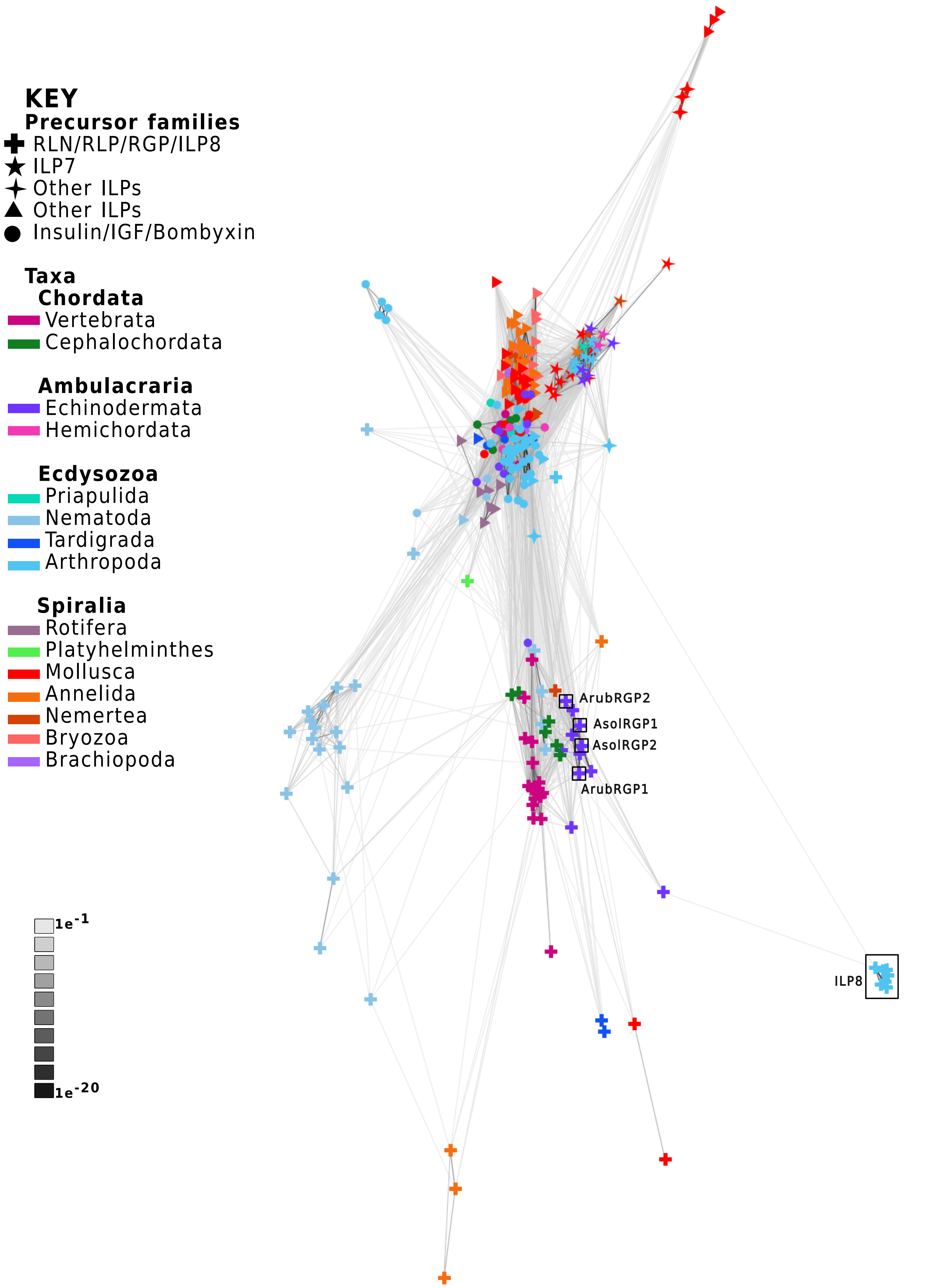
KEY
Precursor families
RLN/RLP/RGP/ILP8
ILP7
Other ILPs
Other ILPs
Insulin/IGF/Bombyxin
Taxa
Chordata
Vertebrata
Cephalochordata
Ambulacraria
Echinodermata
Hemichordata
Ecdysozoa
Priapulida
Nematoda
Tardigrada
Arthropoda
Spiralia
Rotifera
Platyhelminthes
Mollusca
Annelida
Nemertea
Bryozoa
Brachiopoda
1e-1
1e-20
ArubRGP2
AsolRGP1
AsolRGP2
ArubRGP1
ILP8
