# Supplementary material for: Receptor deorphanization in starfish reveals the evolution of relaxin signaling as a regulator of reproduction
Source: BMC Biol. 2025 Feb 25;23:59. doi: 10.1186/s12915-025-02158-2 (PMC11863921; doi:10.1186/s12915-025-02158-2)
Supplement: Supplementary file 2 — Additional file 2. Fig. S2. Alignment of the sequences of the B-chains (upper) and A-chains (lower) of relaxin-type peptides in bilaterian taxa. The aligned amino acids are highlighted in black if the residue is present in at least 70% of the sequences or highlighted in grey if conservative amino acid substitutions are present in at least 70% of the sequences. The conserved cysteine motifs are underlined with red stars and the position of putative disulphide bonds are shown in the alignment with red lines. Note that presence of an alanine residue in the B-chain (red) and glycine-isoleucine dipeptide sequence (red) in the A-chain that is conserved between starfish RGP2-type peptides and the Drosophila Dilp8-type peptide and/or the O. fusiformis (phylum Annelida) relaxin-like peptide. Species and peptide names are highlighted in taxon-specific colours: purple (Vertebrata), green (Cephalochordata), light blue (Echinodermata), orange (Annelida), red (Arthropoda). Species name abbreviations are as follows: Hsap (H. sapiens), Pmar (P. marinus), Bflo (B. floridae), Bbel (B. belcheri), Arub (A. rubens), Asol (A. cf. solaris), Mgla (M. glacialis), Pmin (P. miniata), Ovic (O. victoriae), Hsca (H. scabra), Amed (A. mediterranea), Ofus (O. fusiformis), Dmel (D. melanogaster). The accession numbers and sequences of the precursors of the relaxin-type peptides included in this figure are listed in Additional file 10: Dataset S3 and Additional file 11: Dataset S4, respectively. [file 12915_2025_2158_MOESM2_ESM.pdf]

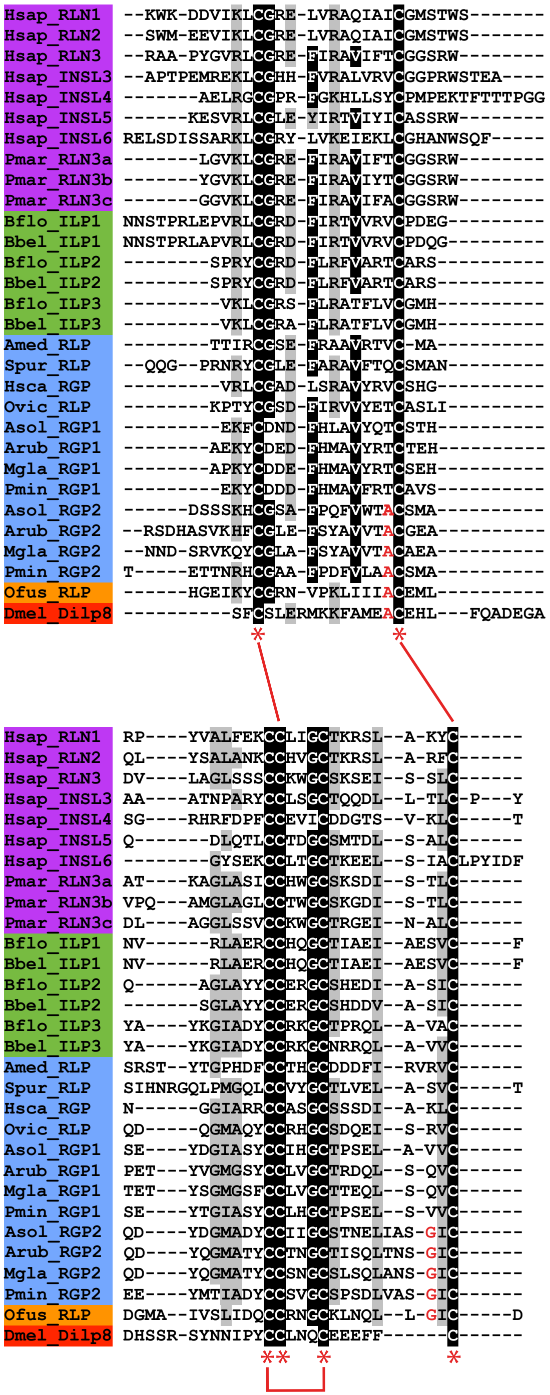

Hsap_RLN1 --KWK-DDVIKLCGRE-LVRAQIAICGMSTWS-------
Hsap_RLN2 --SWM-EEVIKLCGRE-LVRAQIAICGMSTWS-------
Hsap_RLN3 --RAA-PYGVRLCGRE-FIRAVIFTCGGSRW--------
Hsap_INSL3 --APTPEMREKLCGHH-FVRALVRVCGGPRWSTEA----
Hsap_INSL4 -------AELRGCGPR-FGKHLLSYCPMPEKTFTTTPGG
Hsap_INSL5 ------KESVRLCGLE-YIRTVIYICASSRW--------
Hsap_INSL6 RELSDISSARKLCGRY-LVKEIEKLCGHANWSQF-----
Pmar_RLN3a -------LGVKLCGRE-FIRAVIFTCGGSRW--------
Pmar_RLN3b -------YGVKLCGRE-FIRAVIYTCGGSRW--------
Pmar_RLN3c -------GGVKLCGRE-FIRAVIFACGGSRW--------
Bflo_ILP1 NNSTPRLEPVRLCGRD-FIRTVVRVCPDEG---------
Bbel_ILP1 NNSTPRLAPVRLCGRD-FIRTVVRVCPDQG---------
Bflo_ILP2 ---------SPRYCGRD-FLRFVARTCARS----------
Bbel_ILP2 ---------SPRYCGRD-FLRFVARTCARS----------
Bflo_ILP3 ---------VKLCGRS-FLRATFLVCGMH----------
Bbel_ILP3 ---------VKLCGRA-FLRATFLVCGMH----------
Amed_RLP --------TTIRCGSE-FRAAVRTVC-MA----------
Spur_RLP --QQG--PRNRYCGLE-FARAVFTQCSMAN---------
Hsca_RGP ---------VRLCGAD-LSRAVYRVCSHG----------
Ovic_RLP --------KPTYCGSD-FIRVVYETCASLI---------
Asol_RGP1 ---------EKFCDND-FHLAVYQTCSTH----------
Arub_RGP1 --------AEKYCDED-FHMAVYRTCTEH----------
Mgla_RGP1 --------APKYCDDE-FHMAVYRTCSEH----------
Pmin_RGP1 ---------EKYCDDD-FHMAVFRTCAVS----------
Asol_RGP2 ------DSSSKHCGSA-FPQFVWTACSMA----------
Arub_RGP2 --RSDHASVKHFCGLE-FSYAVVTACGEA----------
Mgla_RGP2 --NND-SRVKQYCGLA-FSYAVVTACAEA----------
Pmin_RGP2 T-----ETTNRHCGAA-FPDFVLAACSMA----------
Ofus_RLP ------HGEIKYCGRN-VPKLIIIACEML----------
Dmel_Dilp8 ----------SFCSLERMKKFAMEACEHL---FQADEGA
Hsap_RLN1 RP----YVALFEKCCLIGCTKRSL--A-KYC------
Hsap_RLN2 QL----YSALANKCCHVGCTKRSL--A-RFC------
Hsap_RLN3 DV----LAGLSSSCCKWGCSKSEI--S-SLC------
Hsap_INSL3 AA----ATNPARYCCLSGCTQQDL--L-TLC-P---Y
Hsap_INSL4 SG----RHRFDPFCCEVICDDGTS--V-KLC-----T
Hsap_INSL5 Q-------DLQTLCCTDGCSMTDL--S-ALC------
Hsap_INSL6 --------GYSEKCCLTGCTKEEL--S-IACLPYIDF
Pmar_RLN3a AT----KAGLASICCHWGCSKSDI--S-TLC------
Pmar_RLN3b VPQ---AMGLAGLCCTWGCSKGDI--S-TLC------
Pmar_RLN3c DL----AGGLSSVCCKWGCTRGEI--N-ALC------
Bflo_ILP1 NV------RLAERCCHQGCTIAEI--AESVC-----F
Bbel_ILP1 NV------RLAERCCHQGCTIAEI--AESVC-----F
Bflo_ILP2 Q------AGLAYYCCERGCSHEDI--A-SIC------
Bbel_ILP2 -------SGLAYYCCERGCSHDDV--A-SIC------
Bflo_ILP3 YA----YKGIADYCCRKGCTPRQL--A-VAC------
Bbel_ILP3 YA----YKGIADYCCRKGCNRRQL--A-VVC------
Amed_RLP SRST--YTGPHDFCCTHGCDDDFI--RVRVC------
Spur_RLP SIHNRGQLPMGQLCCVYGCTLVEL--A-SVC-----T
Hsca_RGP N------GGIARRCCASGCSSSDI--A-KLC------
Ovic_RLP QD-----QGMAQYCCRHGCSDQEI--S-RVC------
Asol_RGP1 SE----YDGIASYCCIHGCTPSEL--A-VVC------
Arub_RGP1 PET---YVGMGSYCCLVGCTRDQL--S-QVC------
Mgla_RGP1 TET---YSGMGSFCCLVGCTTEQL--S-QVC------
Pmin_RGP1 SE----YTGIASYCCLHGCTPSEL--S-VVC------
Asol_RGP2 QD----YDGMADYCCIIGCSTNELIAS-GIC------
Arub_RGP2 QD----YQGMATYCCTNGCTISQLTNS-GIC------
Mgla_RGP2 QD----YQGMATYCCSNGCSLSQLANS-GIC------
Pmin_RGP2 EE----YMTIADYCCSVGCSPSDLVAS-GIC------
Ofus_RLP DGMA--IVSLIDQCCRNGCKLNQL--L-GIC-----D
Dmel_Dilp8 DHSSR-SYNNIPYCCLNQCEEEFF------C------
