# Supplementary material for: Receptor deorphanization in starfish reveals the evolution of relaxin signaling as a regulator of reproduction
Source: BMC Biol. 2025 Feb 25;23:59. doi: 10.1186/s12915-025-02158-2 (PMC11863921; doi:10.1186/s12915-025-02158-2)
Supplement: Supplementary file 3 — Additional file 3. Fig. S3. BLOSUM62 cluster map showing that the A. cf. solaris and A. rubens G-protein coupled receptors AsolRXFP/LGR3, ArubRXFP/LGR3, AsolLGR4 and ArubLGR4 (boxed) are positioned in a cluster (type C1) that contains vertebrate RXFP1/RXFP2-type receptors and arthropod LGR3-type and LGR4-type receptors. Nodes are labelled with receptor-type specific symbols and phylum-specific colours, as shown in the key. Connections represent BLAST relationships with a P value > 1e-10. Accession numbers for receptor sequences included in this figure are listed in Additional file 12: Dataset S5 and the sequences of the receptor proteins in FASTA format are listed in Additional file 13: Dataset S6. [file 12915_2025_2158_MOESM3_ESM.pdf]

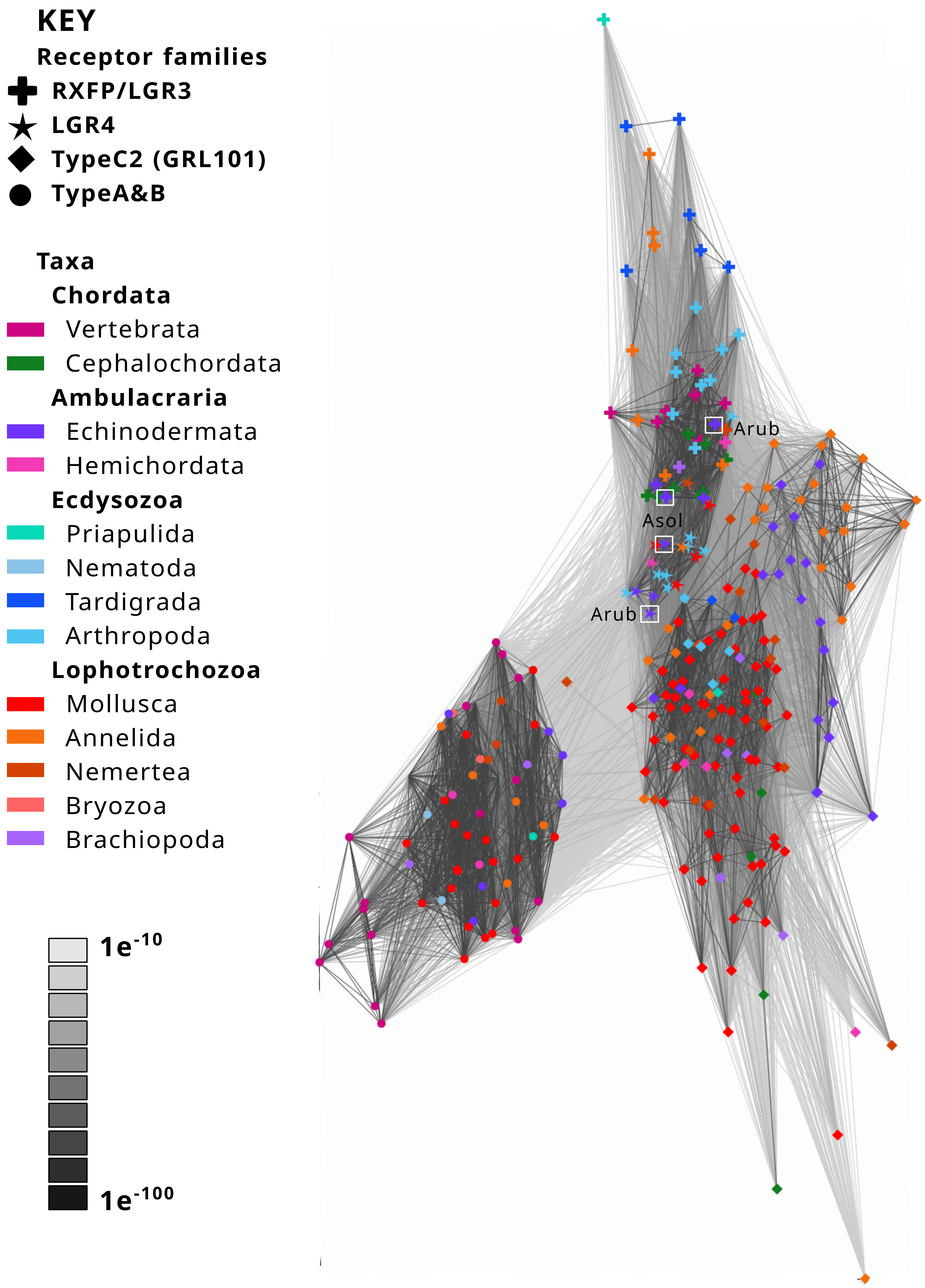
KEY
Receptor families
RXFP/LGR3
LGR4
TypeC2 (GRL101)
TypeA&B
Taxa
Chordata
Vertebrata
Cephalochordata
Ambulacraria
Echinodermata
Hemichordata
Ecdysozoa
Priapulida
Nematoda
Tardigrada
Arthropoda
Lophotrochozoa
Mollusca
Annelida
Nemertea
Bryozoa
Brachiopoda
1e-10
1e-100
Arub
Asol
Arub
