# Supplementary figures and images for: Receptor deorphanization in starfish reveals the evolution of relaxin signaling as a regulator of reproduction
Source: BMC Biol. 2025 Feb 25;23:59. doi: 10.1186/s12915-025-02158-2 (PMC11863921; doi:10.1186/s12915-025-02158-2)

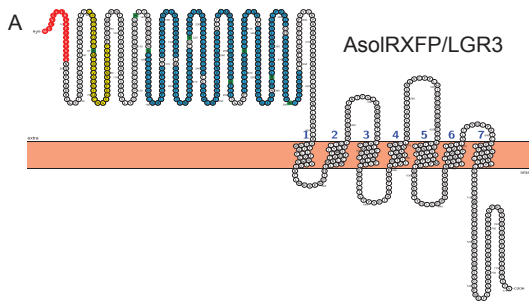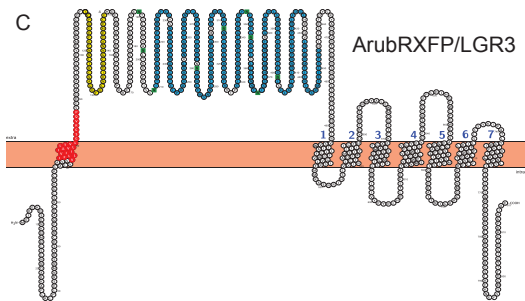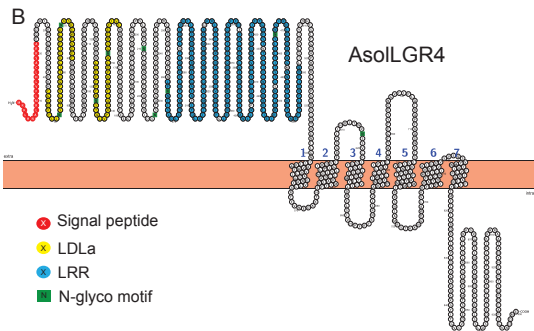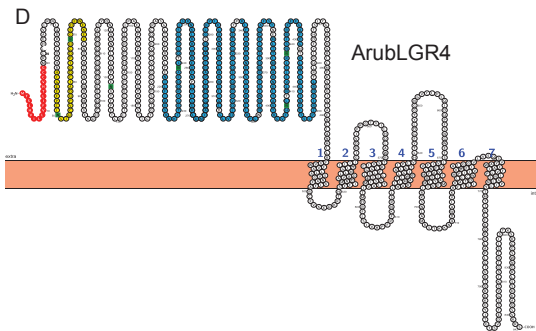

- Signal peptide
- LDLa
- LRR
- N-glyco motif

Supplement: Supplementary file 4 — Additional file 4. Fig. S4. Predicted membrane topology of the A. cf. solaris G-protein coupled receptors AsolRXFP/LGR3 (A) and AsolLGR4 (B) and the A. rubens G-protein coupled receptors ArubRXFP/LGR3 (C) and ArubLGR4 (D). All four receptors have seven predicted transmembrane domains, as expected for G-protein coupled receptors. The predicted extracellular N-terminal region of the receptors contain several predicted glycosylation sites (green), a low-density lipoprotein receptor class A (LDLa, yellow) module and leucine-rich repeats (LRRs, blue), which are a characteristic feature of leucine-rich repeat type G-protein coupled receptors. However, a key difference between the receptors is the position of the predicted signal peptide (red), which in A. solRXFP/LGR3, AsolLGR4 and ArubLGR4 is located at the N-terminus of the protein, whilst in ArubRXFP/LGR3 it is located internally and with a valine residue located at the start of the predicted signal peptide. In silico analysis of the amino acid sequences of the four receptors was performed using Protter (https://wlab.ethz.ch/protter). [file 12915_2025_2158_MOESM4_ESM.pdf]

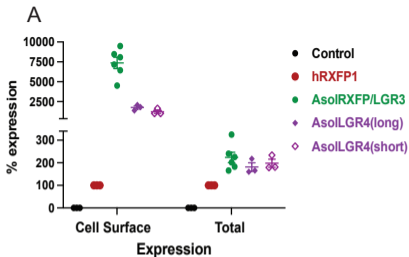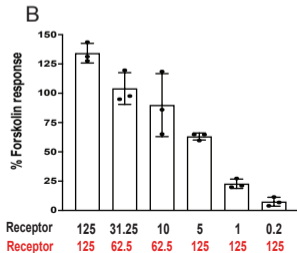

Supplement: Supplementary file 5 — Additional file 5. Fig. S5. A. Analysis of the cell surface and total expression of A. cf solaris receptors AsolRXFP/LGR3, AsolLGR4(long) and AsolLGR4(short) compared to human RXFP1 (hRXFP1). B. Constitutive activity demonstrated by AsolRXFP/LGR3. pCRE reporter gene activity in the absence of ligand with different amounts of transfected receptor is shown. Data are expressed as the % forskolin activity. Assays were performed in triplicate within each assay and were repeated at least three times and are presented as mean values with error bars (S.E.M.). [file 12915_2025_2158_MOESM5_ESM.pdf]

# ArubRXFP/LGR3

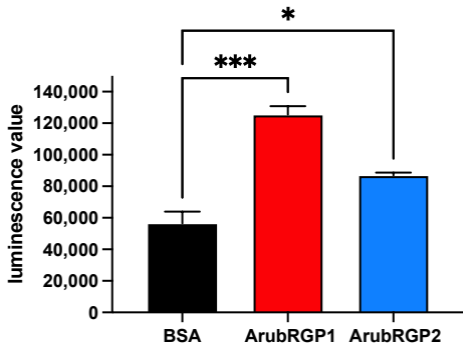

Supplement: Supplementary file 6 — Additional file 6. Fig. S6. Luminescence responses of CHO-K1 cells expressing ArubRXFP/LGR3 when exposed to BSA (control), ArubRGP1RGP (10–5 M) and ArubRG2 (10–5 M) 30 s. The data were analysed by one-way ANOVA with Bonferroni’s multiple comparisons post hoc test, revealing that luminescence responses in cells exposed to ArubRGP1 and ArubRGP2 are significantly higher (***p < 0.001, *p < 0.1) than in cells exposed to BSA media. The data shown are representative of three independent experiments. [file 12915_2025_2158_MOESM6_ESM.pdf]

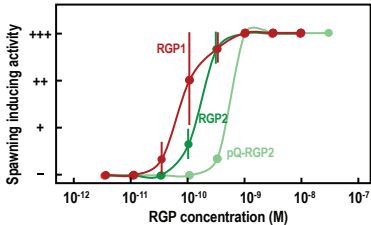

Supplement: Supplementary file 7 — Additional file 7. Fig. S7. AsolRGP1 and AsolRGP2 cause dose-dependent induction of spawning of ovarian fragments from A. sf. solaris. Asol-pQ-RGP2, a modified form of AsoRGP2 in which the N-terminal residue of the A-chain (glutamine, Q) was substituted with pyroglutamate (pQ), was also tested but was less potent than AsolRGP1 and AsolRGP2. + + + denotes spawning occurred and most of oocytes were matured, + + denotes about 50% oocytes were matured, + denotes a few oocytes were matured, and – denotes no spawning occurred. Symbols and bars represent the mean for four separate assays using ovaries from four different animals and standard error of the mean (SEM), respectively. [file 12915_2025_2158_MOESM7_ESM.pdf]
