# Supplementary material for: Receptor deorphanization in starfish reveals the evolution of relaxin signaling as a regulator of reproduction
Source: BMC Biol. 2025 Feb 25;23:59. doi: 10.1186/s12915-025-02158-2 (PMC11863921; doi:10.1186/s12915-025-02158-2)
Supplement: Supplementary file 14 — Additional file 14. Dataset S7. Sequences and accession numbers of the precursor proteins shown in Fig. 2, with the position and phase of introns in the corresponding gene indicated by black highlighting and numbers. [file 12915_2025_2158_MOESM14_ESM.docx]

>Hsap_Relaxin1/NP_008842.1

MPRLFLFHLLEFCLLLNQFSRAVAAKWKDDVIKLCGRELVRAQIAICGMSTWSKRSLSQEDA

1

PQTPRPVAEIVPSFINKDTETIIIMLEFIANLPPELKAALSERQPSLPELQQYVPALKDSNLSFEEFKKLIRNRQSEAADSNPSELKYLGLDTHSQKKRRPYVALFEKCCLIGCTKRSLAKYC

185

>Hsap_Relaxin2/NP_604390.1

MPRLFFFHLLGVCLLLNQFSRAVADSWMEEVIKLCGRELVRAQIAICGMSTWSKRSLSQEDA

1

PQTPRPVAEIVPSFINKDTETINMMSEFVANLPQELKLTLSEMQPALPQLQQHVPVLKDSSLLFEEFKKLIRNRQSEAADSSPSELKYLGLDTHSRKKRQLYSALANKCCHVGCTKRSLARFC

185

>Hsap_Relaxin3/NP_543140.1 1

MARYMLLLLLAVWVLTGELWPGAEARAAPYGVRLCGREFIRAVIFTCGGSRWRRSDILAHEAMGDTFPDADADEDSLAGELDEAMGSSEWLALTKSPQAFYRGRPSWQGTPGVLRGSRDVLAGLSSSCCKWGCSKSEISSLC

142

>Hsap_INSL3/NP_005534.2

MDPRLPAWALVLLGPALVFALGPAPTPEMREKLCGHHFVRALVRVCGGPRWSTEARRPATGG

1

DRELLQWLERRHLLHGLVADSNLTLGPGLQPLPQTSHHHRHHRAAATNPARYCCLSGCTQQDLLTLCPY

131

>Hsap_INSL4/NP_002186.1 1

MASLFRSYLPAIWLLLSQLLRESLAAELRGCGPRFGKHLLSYCPMPEKTFTTTPGGWLLESGRPKEMVSTSNNKDGQALGTTSEFIPNLSPELKKPLSEGQPSLKKIILSRKKRSGRHRFDPFC

1

CEVICDDGTSVKLCT

139

>Hsap_INSL5/AAD29686.1 1

MKGSIFTLFLFSVLFAISEVRSKESVRLCGLEYIRTVIYICASSRWRRHLEGIPQAQQAETGNSFQLPHKREFSEENPAQNLPKVDASGEDRLWGGQMPTEELWKSKKHSVMSRQDLQTLCCTDGCSMTDLSALC

135

>Hsap_INSL6/NP_009110.2

MPRLLRLSLLWLGLLLVRFSRELSDISSARKLCGRYLVKEIEKLCGHANWSQFRFEEETPFSRLIAQASEKVEAYSPYQFESPQTASPARGRGTNPVSTSWEEAVNSWEMQSLPEYKDKKGYSPLGKTREFSSSHNINVYIHENAKFQKK

1

RRNKIKTLSNLFWGHHPQRKRRGYSEKCCLTGCTKEELSIACLPYIDFKRLKEKRSSLVTKIY

213

>Bflo_ILP1/XM_035832785.1

MEFPKPSLILVVLTVAMLVVTEANNSTPRLEPVRLCGRDFIRTVVRVCPDEGRRRRSLEEFEIPSKTSKKEGQTEESTKGLADVLGLRKWREAHRLTKRNVRLAERCCHQGCTIAEIAESVCF

1

123

>Bflo_ILP2/XP_035689227.1 1

MRLLGLVVAVLVLAAAASVPVSKRSPRYCGRDFLRFVARTCARSKRSWQADERRRWEGPYHLPGLTKHYDRTGYHVDRLPFQGRRPEDVHTVTRKQAGLAYYCCERGCSHEDIASIC

117

>Bflo_ILP3/XP_035664330.1 1

MGRLYRTVVVTVLLLSVLSPTRPVFGVKLCGRSFLRATFLVCGMHKRSGHDASRQADFSEPRPPGLKPPSIRTPRSALLRDEGPLHSTLSSLPSYTTTFPTLAPFDFWNVASVIDLARMSADETREEEKRRGRGRGKGRGRGKGRGRPRGRGRYAYKGIADYCCRKGCTPRQLAVAC

177

>Arub_RGP1/ ALJ99970.1 1

MANYRLILEATCLLVLLINTALYAEAAEKYCDEDFHMAVYRTCTEHKRSGRSAFSLNDFFRSNSKRTAGSPRPDDDFFLTMQKRPETYVGMGSYCCLVGCTRDQLSQVC

109

>Asol_RGP1/ XP_022109020.1

MLPLIGTMANNLRRRFQATCLVLLILQATINTGAVGEKFCDNDFHLAVYQTCSTHKRGDGEPVLSLKDVLTGSRLRGNIKRSFGSTLEDEAFFASRLVKRSEYDGIASYCCIHGCTPSELAVVC

1

124

>Arub_RGP2/ XP_033633574.1 1

MTSCSHQMLALLSAVYILIFFLGGLPAVHARSDHASVKHFCGLEFSYAVVTACGEAKRSIRSAPFFDMFPVFKSPERIPADFDDSSMIHVRKRQDYQGMATYCCTNGCTISQLTNSGIC

119

>Asol_RGP2/ XR_002597536.1(mRNA). 1

MTSKYRLILASVPAVVFVIATLSLSMVQADSSSKHCGSAFPQFVWTACSMAKRSNRSPRSLDDLLETFKSARHLDISYRTPIRLSKRQDYDGMADYCCIIGCSTNELIASGIC

113

>Ofus_ILP2(RLP)/ OFUSG19272.1 1

MSSVVRYLRAMVLVATIVANSNVMGHGEIKYCGRNVPKLIIIACEMLKKRSTETQALGDLKNLAKKFYIDGLLRELWHEGKLLDSNGHPLNAKSGSVPTSGDTVSLVRGANNEQKDRSDKKAKLNLLKEMLPGLIEMMQDTPQQEHQREVLAEPTNTQDTYGKSNDELVSLLGLLGLSNSGSQSGKHVTAVSENPAGVVKKDFDPLRNDGRLFHYMQTKRDGMAIVSLIDQCCRNGCKLNQLLGICD

247

>Dmel_Dilp8/ NM_140692.3

0 1

MSSKLHMCRWMLLVIGVCCLMGSSSGSFCSLERMKKFAMEACEHLFQADEGARRDRRSIEFAHHHLNRLG

SGKTHNKHHYISRSSYPMGGYLKVTREHFNRLSELDIFPRYKPIKPHHEKKHRFKRDHSSRSYNNIPYCC

LNQCEEEFFC

150

>Skow_ILP7/ XP_006815151.1 2

MSTSLVGLVFCLTLSFMLCQIQALTVENEETITFSELNTMYGTRTLTDWQGKWTSETIHACGSNLYRISEYVCYVDIHKSPDRKRTDDAFVDSAVAHDFLRGLMEKRTLRRYRRTSATSECCADDGGCVWEELAEYCTHQREVRTMDE

1

148

>Arub_ILP7/ XP_033629466.1 2

MERLISIHIKLLALILLFLLPCLRAAKPHLPVDQWRSRSKADWIRLWNTERHVNVCNEHLQPVFDAACQNDVRKITKRTGEEFVKEWAAKDFLIGSKRKRGLNEECCHEDTGCVWEEVAEYCKKHGREKHKPGSTVAQGKQGR

1

143

>Asol_ILP7/ XP_022079304.1 2

MDRLMGLRIKVSTAILLLLLPCLRAAAPHLPVEQWNSRSKADWVKLWNTERHVNTCNEALLPVWDVACQN

DIRKITKRMGREFLNEWTAKNFLAGSKRRKRGLNEECCHEDLGCVWEEVAEYCVMHGREKHEDGSPVRGK

PGRRR 1

145

>Cvir_ILP7/XP_022286880.1 2

MVLVPLSVWTFSWILTHMKTAALHYQSYNPAFLQRTEAEWRSLWHTDCHRVCHYELDQHVDLACRMDIYRIRKRSAPRQDPESIGSNKHFQKRKDIFIEKPTSMKFLSPTSSVSIRRKRNVLNECCYSKGCSWEEFAEFCQFSIRLPATNINSCTS 1

156

>Ofus_ILP7/CAH1799283.1

2

MVRRALYLTVSSYTPTLLACVAILLVNYCNGAARARDSQVALEKIKELFLARTPHEWKQNWHVDCHRRCRSELIHHIRIACEKDIYRLDKRLADSNNPFMGEDEANSFFKVRSRRDLRLKRRGIMQECCYDKPCSWEEFAESCHTHSRLPASRINHCKK 1

>Dmel_Dilp7/NP_570070.1 2

MTRMIIQNSGSWTLCGAVLLFVLPLIPTPEALQHTEEGLEMLFRERSQSDWENVWHQETHSRCRDKLVRQ

LYWACEKDIYRLTRRNKKRTGNDEAWIKKTTTEPDGSTWLHVNYANMFLRSRRSDGNTPSISNECCTKAG

CTWEEYAEYCPSNKRRNHY 1

159

>AFK93533.1 preproinsulin [Homo sapiens] 1

MALWMRLLPLLALLALWGPDPAAAFVNQHLCGSHLVEALYLVCGERGFFYTPKTRREAEDLQVGQVELGG

GPGAGSLQPLALEGSLQKRGIVEQCCTSICSLYQLENYCN

110

>Hsap_IGF1/NP_001104753.1

0 1

MGKISSLPTQLFKCCFCDFLKVKMHTMSSSHLFYLALCLLTFTSSATAGPETLCGAELVDALQFVCGDRGFYFNKPTGYGSSSRRAPQTGIVDECCFRSCDLRRLEMYCAPLKPAKSARSVRAQRHTDMPKTQKYQPPSTNKNTKSQRRKGSTFEERK 0

158

>Hsap_IGF2/KAI4069443.1 1

MGIPMGKSMLVLLTFLAFASCCIAAYRPSETLCGGELVDTLQFVCGDRGFYFSRPASRVSRRSRGIVEEC

CFRSCDLALLETYCATPAKSERDVSTPPTVLPDNFPRYPVGKFFQYDTWKQSTQRLRRGLPALLRARRGH

0

VLAKELEAFREAKRHRPLIALPTQDPAHGGAPPEMASNRK

180

>Arub_IGF1/ XP_033628588.1

0 1

MFRNTSTMRALLLLDVIFVALVLPITAWPKICGEQLVETVSLVCSTRGFYSHRDSKRDVEVFQNERAAKSFLGSRIGSRQRRRTGRIATECCDRICSFDIVESYCNPWPVAIESRDPPLSPVAPGRVREDKSADVDYMYNPDVVDVEEANSVIQREEDLIDDIETQEQEIEQDEEQNMQTLPEEDAEDTDIREPEDVEESFPVPVPTKKRRKVEGRRSKESKNKGGKSEGKNKKRSGSREGGRSSRRSRGKSSRSKKQRDGRERSKRWEGLDTSHPVKEPTARSVLGRVDTRPFRNFLYN

0

RYTVDEKRDTERESYRAVAPLTGYNSHRGGSQPDNHPTLAALYNLAVKLAKGLQH

355

>Asol_IGF2/ XP_022102680.1

MRAHDECVSGDSGHTLLFSNSSTMRALLLIDVIFVIMAAPISAWQRICGEQLVETVSIVCNTRGFYSHRE

KREAELFQDERTAKSFLGTHIGSRQRRRTGRIATECCEKVCSYDIVESYCNPWPVVEDRDDPMLAPVAPG

RVRQDKSADADLLLRPDIAEISEDKSSLLRQAAEKDEPIDDLDTLENEYADGGNVMLQAREGVKEEGAEL

GKEMEEGGEKMPFPEVVPTKKRRRVEGRRSRENSRDRNGKSEGKSKRKSGSREGGRSFRRRGKNSRRKKG

RDGRERSKRWEALVTSHPPTKDIVDALRSALRAAGRSPSRVAGSFAPDEQRSPLGTPAGLQHRSGLDPSG

KPRPSYRVTQKDNQSTLEALYNLALRLSRPRRG

383

>Arub_IGF2/ XP_033629286.1 1

MNQYQLIVLFEVLAHASMLNYASPVQLCGRELTETLRSICGDRGYYSPGQSFSRRAPTHDGIATRCCQSLCESSILETYCNLPAPPSQTQPSTAAPTTTTKMAPLTEDRRTKDVVVDYSDQLATEGSQMSRVDGVLTHDTVTNRSKTTTESNEGSYDNEEGAPYDKPDDSSPSERGESIQDEDNEVNKPEPNNIRDNSKERGRNRTHKGVSSERRANNSRRRGLSSERRGSSSSRREEKLRRRRQRHRERELREQRKQSNSKRKSKGDKKDHSVAATTPLAVQERPLKNGGRNSTSGEHSSVNGTETDTAGAGSPEVKKDDLITTITAVLSDMIGFQPDNGNR 0

343

>Asol_IGF2/ XP_022102687.1

MDTPNKMNQFRLIVLLEVMAYTVLPNQAAVIQVCGNDLLDALKSVCGDRGFYSPPPGYSRRTPATQTGIA

TRCCISYCETSVLEKYCNPPSTSQSQTAAAPPRITTTPDERRANEIVVDETGQTGNTNSQMLRGGNAMGA

GSRANGTKAPPTEVVDGRSDDDDDAAGEINTSERVGSLTEPDEETGRDVATNRPHKTHSKERSKNRTSKS

ERRRRRTNRRRSSSERRMLSSERKREDATRKLRRKEQRLSRKQPHSNKRKSKLEKKGSESVTTPVAVQTA

DHPFKHGAYNSTTGDLSLVNVTDSDTAPSSDTKKDGFFTTITAVLRDVIGFQHADDGNR

339

>Dmel_Dilp1/ NP_648359.1

MFSQHNGAAVHGLRLQSLLIAAMLTAAMAMVTPTGSGHQLLPPGNHKLCGPALSDAMDVVCPHGFNTLPR

KRESLLGNSDDDEDTEQEVQDDSSMWQTLDGAGYSFSPLLTNLYGSEVLIKMRRHRRHLTGGVYDECCVK

TCSYLELAIYCLPK

154

>Dmel_Dilp2/ NP_524012.1 1

MSKPLSFISMVAVILLASSTVKLAQGTLCSEKLNEVLSMVCEEYNPVIPHKRAMPGADSDLDALNPLQFV

QEFEEEDNSISEPLRSALFPGSYLGGVLNSLAEVRRRTRQRQGIVERCCKKSCDMKALREYCSVVRN

137

>Dmel_Dilp3/ NP_648360.2 1

MGIEMRCQDRRILLPSLLLLILMIGGVQATMKLCGRKLPETLSKLCVYGFNAMTKRTLDPVNFNQIDGFE

DRSLLERLLSDSSVQMLKTRRLRDGVFDECCLKSCTMDEVLRYCAAKPRT

120

>Dmel_Dilp4/ NP_648361.1 1

MSLIRLGLALLLLLATVSQLLQPVQGRRKMCGEALIQALDVICVNGFTRRVRRSSASKDARVRDLIRKLQ

QPDEDIEQETETGRLKQKHTDADTEKGVPPAVGSGRKLRRHRRRIAHECCKEGCTYDDILDYCA

134

>Dmel_Dilp5/ NP_996037.2 1

MMFRSVIPVLLFLIPLLLSAQAANSLRACGPALMDMLRVACPNGFNSMFAKRGTLGLFDYEDHLADLDSS

ESHHMNSLSSIRRDFRGVVDSCCRKSCSFSTLRAYCDS

108

>Dmel_Dilp6/ CCF74153.1

MVLKVPTSKVLLVLATLFAVAAMISSWMPQVAASPLAPTEYEQRRMMCSTGLSDVIQKICVSGTVALGDV

FPNSFGKRRKRDLQNVTDLCCKSGGCTYRELLQYCKG

107

>Bmor_Bombyxin/ XP_004934106.1

MKILLAIALMLSTVMWVSTQQPQRVHTYCGRHLARTLADLCWEAGVDKRSGAQFASYGSAWLMPYSEGRG

KRGIVDECCLRPCSVDVLLSYCF

93
