# Supplementary material for: Receptor deorphanization in starfish reveals the evolution of relaxin signaling as a regulator of reproduction
Source: BMC Biol. 2025 Feb 25;23:59. doi: 10.1186/s12915-025-02158-2 (PMC11863921; doi:10.1186/s12915-025-02158-2)
Supplement: Supplementary file 15 — Additional file 15. Dataset S8. Sequences of the receptors shown in Fig. 4, showing the positions of different domains and with the position of introns in the corresponding gene indicated by black highlighting. [file 12915_2025_2158_MOESM15_ESM.docx]

- **MTSGSVFFYILIFGK**: predicted signal peptide
- QDVKCSLG: LDLa domain
- VPHCHHA: Linker
- PPDCAFRGHNRIEDNHTRLPHIHN (colourful sequences): Leucine rich repeat containing domains.
- GNIFVICMRPYIRSENKLYAM:TM domain
- CS: position of intron
- Definition: LDLa domain sequence: <https://pubs.acs.org/doi/pdf/10.1021/bi500797d>


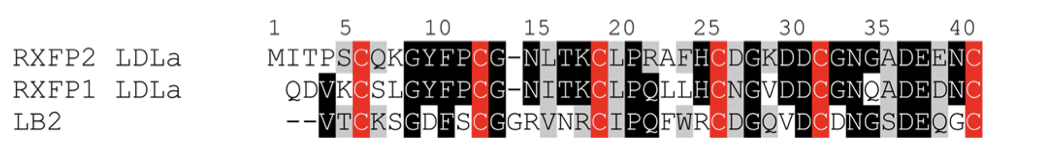


>Hsap_RXFP1

**MTSGSVFFYILIFGKYFSHGGG**QDVKCSLGYFPCGNITKCLPQLLHCNGVDDCGNQADEDNCGDNNGWSLQFDKYFASYYKMTSQYPFEAETPECSFHFFLFITLLFLVPHCHHALPLPLDSVVGSVPVQCLCQGLELDCDETNLRAVPSVSSNVTAMSLQWNLIRKLPPDCFKNYHDLQKLYLQNNKITSISIYAFRGLNSLTKLYLSHNRITFLKPGVFEDLHRLEWLIIEDNHLSRISPPTFYGLNSLILLVLMNNVLTRLPDKPLCQHMPRLHWLDLEGNHIHNLRNLTFISCSNLTVLVMRKNKINHLNENTFAPLQKLDELDLGSNKIENLPPLIFKDLKELSQLNLSYNPIQKIQANQFDYLVKLKSLLEGIEISNIQQRMFRPLMNLSHIYFKKFQYCGYAPHVRSCKPNTDGISSLENLLASIIQRVFVWVVSAVTCFGNIFVICMRPYIRSENKLYAMSIISLCCADCLMGIYLFVIGGFDLKFRGEYNKHAQLWMESTHCQLVGSLAILSTEVSVLLLTFLTLEKYICIVYPFRCVRPGKCRTITVLILIWITGFIVAFIPLSNKEFFKNYYGTNGVCFPLHSEDTESIGAQIYSVAIFLGINLAAFIIIVFSYGSMFYSVHQSAITATEIRNQVKKEMILAKRFFFIVFTDALCWIPIFVVKFLSLLQVEIPGTITSWVVIFILPINSALNPILYTLTTRPFKEMIHRFWYNYRQRKSMDSKGQKTYAPSFIWVEMWPLQEMPPELMKPDLFTYPCEMSLISQSTRLNSYS

1 2

**VFVWVVSAVTCFGNIFVIC**MRPYIRSENKL**YAMSIISLCCADCLMGIYLFVIGGF**DLKFRGE

3 4

YNKHAQLWMESTHCQ**LVGSLAILSTEVSVLLLTFLTL**EKYICIVYPFRCVRPGKCRT**ITVLI**

5

**LIWITGFIVAFIPL**SNKEFFKNYYGTNGVCFPLHSEDTESIGAQ**IYSVAIF**LG**INLAAFIII**

6

**VFSY**GSMFYSVHQSAITATEIRNQVKKEMILAKR**FFFIVFTDALCWIPIFVVKFLSLLQV**EI

7

PGTI**TSWVVIFILPINSALNPILYTLT**

>Hsap_RXFP2

MIVFLVFKHLFSLRLIT**MFFLLHFIVLINVKDFALTQG**SMITPSCQKGYFPCGNLTKCLPRAFHCDGKDDCGNGADEENCGDTSGWATIFGTVHGNANSVALTQECFLKQYPQCCDCKETELECVNGDLKSVPMISNNVTLLSLKKNKIHSLPDKVFIKYTKLKKIFLQHNCIRHISRKAFFGLCNLQILYLNHNCITTLRPGIFKDLHQLTWLILDDNPITRISQRLFTGLNSLFFLSMVNNYLEALPKQMCAQMPQLNWVDLEGNRIKYLTNSTFLSCDSLTVLFLPRNQIGFVPEKTFSSLKNLGELDLSSNTITELSPHLFKDLKLLQKLNLSSNPLMYLHKNQFESLKQLQSLDLERIEIPNINTRMFQPMKNLSHIYFKNFRYCSYAPHVRICMPLTDGISSFEDLLANNILRIFVWVIAFITCFGNLFVIGMRSFIKAENTTHAMSIKILCCADCLMGVYLFFVGIFDIKYRGQYQKYALLWMESVQCRLMGFLAMLSTEVSVLLLTYLTLEKFLVIVFPFSNIRPGKRQTSVILICIWMAGFLIAVIPFWNKDYFGNFYGKNGVCFPLYYDQTEDIGSKGYSLGIFLGVNLLAFLIIVFSYITMFCSIQKTALQTTEVRNCFGREVAVANRFFFIVFSDAICWIPVFVVKILSLFRVEIPDTMTSWIVIFFLPVNSALNPILYTLTTNFFKDKLKQLLHKHQRKSIFKIKKKSLSTSIVWIEDSSSLKLGVLNKITLGDSIMKPVS

1 2

**IFVWVIAFITCFGNLFVI**GMRSFIKAENTTHAMSIK**IL**CC**ADCLMGVYLFFVGIFDI**KYRGQ

3 4

YQKYALLWMESVQC**RLMGFLAMLSTEVSVLLLTYLTL**EKFLVIVFPFSNIRPGKRQ**TSVILI**

5

**CIWMAGFLIAVIPFW**NKDYFGNFYGKNGVCFPLYYDQTEDIGSKGYSLG**IF**LG**VNLLAFLII**

6

**VFSYITMF**CSIQKTALQTTEVRNCFGREVAVANR**FFFIVFSDAICWIPVFVVKIL**SLFRVEI

7

PDT**MTSWIVIFFLPVNSALNPILY**

>Arub_RXFP/LGR3

MEIKEKDSENKQMDLSLNNDAKKAPLRLPLESRNMNSRYSRCNSFQAECRSLGKRCKLALVAVV**VIFLLVIIIAVL|LTVLGSG**RVQPSEDDAYAVTVSAEYEMNGNVGDFTCPQGEFRCNNLTRCVAQQFQCNKIDDCGNNADEMECEHDEGWIKTFDEKIPIRVQPKREPSTECSLSGFPDVCICYENTSLKCVYANLTKVPPGISSNITHLNLRGNQIIFEDGVFEGYSNLQSLNLMDNGIIELPVDVFRGLRNLDKLYLSDNKIKFLIPGTFRYLNNVTWLFMSNNELAALGEGVFQGMDKLYWLMLPNNSISSLARSVAFRDTPSLVWFDISENPLGRLSPYNFSLPGNVSIISLLTINNCNISSIHRDTFRSFRGLSVLHLSHNSIKYFPSGLFRNLSKLADLHIDNNPATSLPEDLFDGLHRIDVLDLQGMTITNINSRMFEELSTLQHIEFSKFDYCRYAPHVRTCKPRSNGISSFEDLLKDGILRVSVWTIALLCFFGNVGVLISRFMMKAENRIHSLVVINLCTADFFMSIYLIIIGFHDVKFRNFFNMYALEWMQGSTCKFAGFLAMFSSEVSVFMLTFISLERFICIVYPYRLHRLTSKEATVVMSVIWFLGALVAWIPLISVGYFVDFYGSNGVCFPLHIHDPWLPGWEYSAFVFLGLNASCFTAIAISYTGMFISIQRTRKATTNIGKRGDMNYAKRFLFVVLTDALCWLPIAILKILSLCSYKIPATLYGWIIVFVLPINSALNPILYTISTTSFSQWFHKHVKLRRRGEGRGSLRFKNEFSSMGDFTYGISDIEHKPGAIAE

1 2

**VSVWTIALLCFFGNVGVLISRF**MMKAENRIHSL**VVINL**CT**ADFFMSIYLIIIGFH**DVKFRNF

3 4

FNMYALEWMQGSTCK**FAGFLAMFSSEVSVFMLTFISL**ERFICIVYPYRLHRLTSKEA**TVVMS**

5

**VIWFLGALVAWIPLISVGYFV**DFYGSNGVCFPLHIHDPWLPGWEYS**AFVFLGLNASCFTAIA**

6

**ISY**TG**MFISI**QRTRKATTNIGKRGDMNYAK**RFLFVVLTDALCWLPIAILKIL**SLCSYKIPAT

7

L**YGWIIVFVLPINSALNPILYTI**

>Asol_RXFP/LGR3

METSEKVQDQKLEMAPGKESAPKPRFLGISRTSRDSKCSQQDRFRDPVWSVPSITRRLQTKQCKAIILCIV**MGLVLLLALVFILVTVTA**NQVPLADHEGSIQAGPAQTEYEELSGDPSEFTCPRGEFQCGNMTRCVAQKFQCNGEDDCGNNADETECEHDEGWIKNFDKQVPVSVQPERRISQECGLYGFPEVCKCYETTNLRCLQGNLTEVPQDVSNNLTHLNLNGNMLKNLEDGAF**G**RYTKLRYLNLMGNGIRELPRDVFRGLMDLDKLFLSSNKISSLKPGTFRFLRNLTWLFLNDNEIEVLDEEVFQGLETVYWLMLQENRIRNLKRGISFRDLPALMWLDISDSPLNHLSPDNFSLSGNPPLSILTMNNCNISTIHGDTLQQFRDLSTLHLSENKIQHFPSGLFRNMINLTDLAIANNLATSLPEDLFDDLVSLDVLNLGGLVIKNISTRMFKGLTNLQHIEFSKFAYCRYAAHVRTCKPKSDGISSFRN

LLKDGILRVSVWTIALLCFVGNVGVLVSRCLMKAENRIHSLVVMNLCTADFCMSIYLFIIGY

HDAKFRNQFNTFALEWMQSSTCKFAGFLAMFSSEVSVFMLTFISLERFICIVYPYRLHRLTS

REAIVIMTIIWFLGALVAWVPLVNVGYFVDFYGSNGVCFPLHIHDPWLQGWEYSAFIFLGLN

ASCFTAIAISYTAMFISIQQTRKATTHIGRRGDMNYAKRFFFVVLTDALCWLPIAILKILSL

CSYQIPATLYGWIVIFVLPINSALNPILYTLSTTSFSQWFHKHIKRRKGKSSNGSVGSKNEF

SCSRGRIGSATDEPTEYSSVPKRSYDSDSSPVAEEKQCSTV

1 2

**VSVWTIALLCFVGNVGVLV**SRCLMKAENRIH**SLVVMNLCTADFCMSIYLFIIGY**

3

HDAKFRNQFNTFALEWMQSSTC**KFAGFLAMFSSEVSVFMLTFISL**ERFICIVYPYRLHRLTS

4

REA**IVIMTIIWFLGALVAWVPLV**NVGYFVDFYGSNGVCFPLHIHDPWLQGWEYS**AFIFLGLN**

5 6

**ASCFTAIAISYTAMF**ISIQQTRKATTHIGRRGDMNYAKR**FFFVVLTDALCWLPIAILKILSL**

7

CSYQIPATL**YGWIVIFVLPINSALNPILYTL**

>Ofus_LGR3

MTLMRIQCRIHSTTTSKMNLREAHTSGRSNYKRT**VRGPYYISHAWFAIGFITFATFFDQGTCSA**DVFVCPLGKFSCGNQSVCLDQNRWCDGKSDCDAGYDEIHENCQFTGGTYDTIVKIFGNDTIRTINQSCHFSNYPSLCECKEAVISCKEKGFTRLPVDLPSNLTRLIFKGNLLKEIRSHAFQRFSRMTVLHLGGNKIERIDNGGFYGLDRLEKLFLQGNNLTTIETGTIGRLHKLEWLWLSENKLQSIAMDEFKYTNLITINLKWNKLTQVETMVGPANGNLAVLFLDNNKIQRLYSTQFRSMQNLQTLTLSYNDISYIAEDAFKNLPSLTDLDLAWNKLTRLHRKVFEPLTELQLLDLSGNPIILQVNGFSGLQKLEVLKLGAISIDNIQIKMFSSLQSLKFIEFKDFHYCSYAPHVRSCDPKGDGISSFNNLLDNPVLRISIWIVACFTCAGNLAVLISRTLIKTDLNLHSIVIRNLCAADLLMGIYLVIVAAQDVRFRETYNSHAYEWMSSHLCQVTGVLAMISCEVSILILVFMSVERYATISFPYRTFRMSSKLAWIILCSLWVTGLLIGIIPLTSENAFGKFYGSNGVCFPLHIHDPYLKGWEYSAFIFIVLNFTSVAVIVFCYVAMLCSIYKTQRLTESTCAQERSFAKRILVIILSDLMCWVPLITIKLMAFGSIRISDSMYAWLAIFILPINSAINPILYTLTIVSFRRKFLQFWRRKYRSGKTKHCSRFQMGRLGSSSLSSATNTFNLPGDISPPTFRTQLNPSISSEDESAENIGKLQNLLAESNDQEHTV

1 2

**ISIWIVACFTCAGNLAVLISR**TLIKTDLNLHS**IVIRNLCAADLLMGIYLVIVAA**QDVRFRET

3 4

YNSHAYEWMSSH**LCQVTGVLAMISCEVSILILVFM**SVERYATISFPYRTFRMSSK**LAWIILC**

5

**SLWVTGLLIGIIPL**TSENAFGKFYGSNGVCFPLHIHDPYLKGWEYS**AFIFIVLNFTSVAVIV**

6

**FCYVAMLCS**IYKTQRLTESTCAQERSFAKR**ILVIILSDLMCWVPLITIKLMAFGSI**RISDS**M**

7

**YAWLAIFILPINSAINPILYTLTIVSF**

>Dmel_LGR3

MVYGRSIAVGFCL**MTVVLLLAAVIFYLSLGPCPA**ASFACDNGTLCVPRRQMCDSRNDCADSSDENPVECGLLYGSKEIADKIVRNAIEKKQQRLISAVSNASGADSTTSMVPRNQSLTLNMTCDIVTYPKACQCGQGTILYCGRYAKLRRFPRLSSEVTNLIIIRNNLTLRDNIFANFTRLQKLTLKYNNISRVPLGSFSGLFHLERLELSHNNVSHLPHGVFLGLHSLQWLFLVNNHLHHLPVEQLRFFRRLEWLVLSRNRLTLRNVQLPKIPTLYEVYLDFNRIEYIGEETFSQLDNLHLLDLQHNLITHIHGRAFANLTNMRDIRLVGNPIKELSGETFLHNTRLEALSLALMPIHISSSLMEPLNISFLNLTGIRYDHIDFEAINSMRNLTYIIYDRFFYCSMTPRVRMCKPSTDGVSSFQDLLSKPVLRYSAWVMATLTIAGNVLVLWGRFIYRDENVAVTMVIRNLALADMLMGFYLVTIGVQDYRYRNEYYKVVLDWITSWQCTLIGTLAVSSSEVSMLILAFMSLERFLLIADPFRGHRSIGNRVMWLALICIWITGVGLAVAPVLLWRTSTLPYYGSYSGTCFPLHIHEAFPMGWLYSAFVFLGVNLLLLVMIAMLYTALLISIWRTRSATPLTLLDCEFAVRFFFIVLTDFLCWVPIIVMKIWVFFNYNISDDIYAWLVVFVLPLNSAVNPLLYTFTTPKYRNQIFLRGWKKITSRKRAEAGNGNVATTTTGTATGSSQHPDDFTIFAKAAMRCH

1 2

**YSAWVMATLTIAGNVLVLWGRF**IYRDENVAVTM**VIRNLALADMLMGFYLVTI**GVQDYRYRNE

3 4

YYKVVLDWI**TSWQCTLIGTLAVSSSEVSMLILAFMSL**ERFLLIADPFRGHRSIGNR**VMWLAL**

5

**ICIWITGVGLAVAPVLLW**RTSTLPYYGSYSGTCFPLHIHEAFPMG**WLYSAFVFLGVNLLLLV**

6

**MIAMLYTALLIS**IWRTRSATPLTLLDCEFAVR**FFFIVLTDFLCWVPIIVMKIWVFFNY**NISD

7

D**IYAWLVVFVLPLNSAVNPLLYTF**

>Arub_LGR4

**MTSLHVILLVFILTHVCCG**MRLKHEAVLRNLQRRSIDVHRHCGEEFPCLNSTQCVPQDAICDGTPDCDNGSDEWEKEECNDFHTNQLWDAMFGEKDDCDDSEEDDDDDCDDDDSASYQNEAGNFLIEAPCENGTFPETCECIVELEKPSAAHRPVRFSTLPAESPTGPDDTSETPSYRSTSTTGTGTQSPVVEGTVIGVKIDCRASGLTSFPRNLPENTLFIDVSDNKITELARDDFANLTQLRILSLSRNKLRHIDDEVFNPLTELERLDMIACGLDEIPARFFASQSGLKYLKLAHNNLKTLARMSLIGLDSLVDLDVRGNQISDLEVGVFEHTPRLFTIHFSENRLSSIPASLLRPLWNLNWISFQRNDISNIEKGAFSTNEMLTTLMLADNKLTAVTRGVFHNLTNLVLLTLRNNSIRRFEEGAFDGMTKLQTLKLTTNPFTSLPLRIFDRLTRLQKIYFDHFSLCGYAPHVRLCMPKSDGISTAENLLANHLLRFGVWFVALLASVGNAFVLFARCFVKEDKKTHSFFIMNLAVADLLMGLYLLIIGTHDVIFRGTYILHDLAWRTSVICKLGGFLSLLSSEVSIMTLAVITMDRFLSIVHPFRFKNRSLLHARLLMAFLWLLGIALGTLPLVHLTYFGDLYYGGNGVCLPLQIDQPFSNGWEFSLVIFVVFNLVAFMFISYAYLMMFVTIRKSNLAMRSTKKNQDWALVKRFTLIVATDLLCWMPIIVVKFIALGGVLVSQSVYAWFAIFVLPINSALNPILYTMTTVLFKQKVLAPLGIVRTQRKKGYITGTSVDETSSGVSKTSGTRLSIISTKSRGGSWNGRLSSHKAQSVKRS*

1 2

**FGVWFVALLASVGNAFVLFA**RCFVKEDKKTH**SFFIMNLAVADLLMGLYLLII**GTHDVIFRGT

3 4

YILHDLAWR**TSVICKLGGFLSLLSSEVSIMTLAVI**TMDRFLSIVHPFRFKNRSL**LHARLLMA**

5

**FLWLLGIALGTLPLV**HLTYFGDLYYGGNGVCLPLQIDQPFSNGWEFS**LVIFVVFNLVAFMFI**

6

**SYAYLMMFVTI**RKSNLAMRSTKKNQDWALVK**RFTLIVATDLLCWMPIIVVKFIALGG**VLVSQ

7

S**VYAWFAIFVLPINSALNPILY**

>Asol_LGR4

**MRKLHNALRMLFVLHLCASRRA**EQDGAIIRHIQRRAVDAHKHCGKDFPCLNSTQCVPQDSICDGTPDCDNGSDEWEVEECRDWNLAKMWDNFFGTKNDDSGEEEEDDLDQGLRPQQTVFDAHGHCGEEFPCMNSTQCVPREAICNGKPDCDNGSDEEEIVCRNKKMRDNIEDIRQEIPCEEGTFPESCDCFVEIEQTRTAELPSLASPYNTTGDASTYRDAYDIDSNSTDGVRYGSEVRGVVIGIRLDCRAKQLTNVPRNLPNNTISLDLSDNKITHLTKKDLTNLPQLRQLLLSRNKLKRMEEGAFKPLTDLETLRMIACDLEDVQQRMFAAQKRLVTLDLRYNKLTRLVKNSLFGLRNVKYFDIRGNQLSEIETGVFEDTPKLYFMLVSQNKLSSIPANLLRPLRELRTLDVHRNDISVIETGAFSTNTKLIELNLRDNKLTEIRRGIFHSLTSTITLSLSNNSIRHLEQDAFAGMNNLQTLKLTKNPFTSLPVGIFDQLISLKAIYFDHFSLCGYAPHVRLCMPKSDGISTAENLLGNILLRFAVWFVALLASLGNAFVLLARCFVNEDKKTHSFFIMNLAVADLLMGLYLLIIGIHDVIFRGSYILHDLTWRNSSVCKLSGFLSLLSSEVSIMTLTVITLDRFLSIVHPFRFKNRSLVHARLLMVFLWLLGIALATIPLLHTAYFGEFYYGGNGVCLPLQIDQPFADGWEFSLVIFVVFNLVAFTFISYAYLMMFMTIRRSNLAMRSTKKNQDWALVKRFTLIVATDFVCWMPIIIVKFVALGGVSVSQSVYAWFAIFVLPINSALNPILYTMTTVLFRQKILAPLGIVKAKRKKGYITGVSVDETSTMSKNSGTRLSIISNKSRGGSLNGRFNSQKKLKNLSSLDSTDESVTCSAAQTTSLKIKKHRAATADYHELPTSDPDCAPSGVNDDME

1 2

**FAVWFVALLASLGNAFVLLA**RCFVNEDKKTHS**FFIMNLAVADLLMGLYLLIIGIH**DVIFRGS

3 4

YILHDLTWRNSSVCK**LSGFLSLLSSEVSIMTLTVITLD**RFLSIVHPFRFKNRSLVHAR**LLMV**

5

**FLWLLGIALATIPLLHTAY**FGEFYYGGNGVCLPLQIDQPFADGWEFS**LVIFVVFNLVAFTFI**

6

**SYAYLMMF**MTIRRSNLAMRSTKKNQDW**ALVKRFTLIVATDFVCWMPIIIV**KFVALGGVSVSQ

7

SV**YAWFAIFVLPINSALNPILY**

>Ofus_LGR4/CAH1799243.1

**MRWCSAIYPAALVTFTVWISCKG**DYTQCSMDEWPCNGTNVCIEKRKLCNDVRDCEMGDDEMDCSDHDADEYWNQLFPKRPDEDWHREVSVNGQQNKRCDSVNVPTECICTQSMKIFCKNKELRDVPSNIPATTTVLDISGNRIDLLRQEHLSNLTNLKELLIMQSDVVSIAHDAFRDLAELTKLHLQSNLLTKIPNALFENTSLREISLSYNSLGELRKGYFKGLHNLKILRLDNCGIKLIEEGTFSELPALQQLYLGTNRIGTIVRSTFDNLSNLRQLYLNENSISMIEAKSFLHLTKLRHLVMHGNFIKVIKKETFLNLKSLLFLDLNTNKIASIEAGSMSSLRSLTSLDLSQNKLTELEVPFGIFRQMKNLSFIYFDDFTMCVYALHVRNCYPKGDGISSMENLLENGILRISVWVVASLACIGNFIVLLGRILIKEDNQIHSFFIKNLALSDLFMGIYLFIIASKDIMYRGEYLLKLKTEWRGSWQCNLAGVLSTVSSEVSVLTLTVITFDRYISIMYPLHLKKRSIATAYTIMLVIWLLCCVLTTMPLFPSDYFGDTFYSSNGVCLPLHVHNPYENGWEYSLFLFVAINFAAFLFISYAYALMFATIRRSQMSLRSTQENQETSLMKRFFFIVMTDFICWIPIIIIKIAALSGAKINKGFYGWVAIFILPVNSALNPILYTLTTKLFKKHFMSRVYRVFWRSNRPADAIETRASGGSVLVRRISSRSSQVVEFDSIPRRDSSLMSRSSRGSSNKSGNKYTNGIQYCARCKSMHSVNSHRPVYRYQDRSENRNSDQSPGLNRELSWKGINPCCASSTMKSPSSSTYTASLTCIVSPERNLEQDQVKMSLMKHHDDC

1 2

**ISVWVVASLACIGNFIVLLGRILI**KEDNQIHS**FFIKNLALSDLFMGIYLFIIA**SKDIMYRGE

3 4

YLLKLKTEWR**GSWQCNLAGVLSTVSSEVSVLTLTVI**TFDRYISIMYPLHLKKRSI**ATAYTIM**

5

**LVIWLLCCVLTTMPLF**PSDYFGDTFYSSNGVCLPLHVHNPYENGWEYS**LFLFVAINFAAFLF**

6

**ISYAYALMFATI**RRSQMSLRSTQENQETSLMK**RFFFIVMTDFICWIPIIIIKIAALSGA**KIN

7

KG**FYGWVAIFILPVNSALNPILYTL**

>Cvir_LGR4

**MNWKVKIKLGFLWIFLSSAS**SRKSLCSTGTFECQRILECIDEDRKCDGVPDCRDMTDEKYCDRAQDHYWNNKYLKRDEADYDLLNRSCVFKDIPSKCTCKGVTMVFCGGGQLRQIPKGIPGKSTVLDLSNNEIEDFSEDDLDLYFVTKINLQHNKLTTIRNNTFRNCPNLQKLNLNSNNIIRLEENAFPVQNKIEDLKIENNLLTVLNAEMFSNLKNLKRLYLGFNRIRYIVVDTFKELQQLKVMSLISNTFTVLENHFFQHLSKLDILYIDENNIFRIEDRTFNPLSSLHTLGLSRNKIIAVSPNLFRGLGNLTHLNLMINHIVSISPTAFTDLTNLRSLDLRQNVFRSLYKDTLRSLPSLHYVYFDEFYMCAYVASEVVCQPFGDGISSRYNLLENGFLRLMVWIVAICACFGNLLVLLGRSVLREDNQVHSFYIKNLSFADMLMGLYLIIIGYHDQIFRGNYLVEDEGWRSSPTCDICGILSTLSNEASVFTLTLITLDRYISITFPLFRRRKSFKFAMMNVCIIWTISLLLSLFPVTFSGYFGNYFYKDNAVCVPFHLHRPWAKGWEYSTFLFLGLNLAAFSFICFAYIHMFIAIKRSTKTVRSHENKERTLVKRFFFIILTDFLCWMPIIIIKFIALSGHHINQDTYAWLIIFVMPINSAINPLLYTLTTKLFKEKVMPKLCCGFKLVRQEPMLKESSSSSSSSSTGRRIRSSVRSSLEKDLCEVGATTLRVSTRKPKGNFDSVTGSGDSCRNGNYLSFKTRSERGNFYHSNSSRICAKESVYIPQRLSTAI

1 2

**LMVWIVAICACFGNLLVLLGR**SVLREDNQVHS**FYIKNLSFADMLMGLYLIIIGY**HDQIFRGN

3 4

YLVEDEGWRSSPTCDICGILSTLSNEAS**VFTLTLITLDRYISITFPLF**RRRKSFKFAMM**NVC**

5

**IIWTISLLLSLFPVTFSGYF**GNYFYKDNAVCVPFHLHRPWAKGWEYST**FLFLGLNLAAFSFI**

6

**CFAYIHMFIAI**KRSTKTVRSHFENKERTLVK**RFFFIILTDFLCWMPIIIIKFI**ALSGHHINQ

7

DTYA**WLIIFVMPINSAINPLLYTL**

>Dmel_LGR4

**MSIAIMPHLPITFTLAILLAIASNEGAQG**VESATRTAIEAIRTGIGTKPETEIADATEAEAPVREVISLLGIIDGAESDILVPDADDKCPGGYFHCNTTAQCVPQRANCDGSVDCDDASDEVNCVNEVDAKYWDHLYRKQPFGRHDNLRIGECLWPNENFSCPCRGDEILCRFQQLTDIPERLPQHDLATLDLTGNNFETIHETFFSELPDVDSLVLKFCSIREIASHAFDRLADNPLRTLYMDDNKLPHLPEHFFPEGNQLSILILARNHLHHLKRSDFLNLQKLQELDLRGNRIGNFEAEVFARLPNLEVLYLNENHLKRLDPDRFPRTLLNLHTLSLAYNQIEDIAANTFPFPRLRYLFLAGNRLSHIRDETFCNLSNLQGLHLNENRIEGFDLEAFACLKNLSSLLLTGNRFQTLDSRVLKNLTSLDYIYFSWFHLCSAAMNVRVCDPHGDGISSKLHLLDNQILRGSVWVMASIAVVGNLLVLLGRYFYKSRSNVEHSLYLRHLAASDFLMGIYLTLIACADISFRGEYIKYEETWRHSGVCAFAGFLSTFSCQSSTLLLTLVTWDRLMSVTRPLKPRDTEKVRIVLRLLLLWGISFGLAAAPLLPNPYFGSHFYGNNGVCLSLHIHDPYAKGWEYSALLFILVNTLSLIFILFSYIRMLQAIRDSGGGMRSTHSGRENVVATRFAIIVTTDCACWLPIIVVKLAALSGCEISPDLYAWLAVLVLPVNSALNPVLYTLTTAAFKQQLRRYCHTLPSCSLVNNETRSQTQTAYESGLSVSLAHLGGGVGGGSGRKRMSHRQMSYL

1. 2

S**VWVMASIAVVGNLLVLLGRYFY**KSRSNVEHSLY**LRHLAASDFLMGIYLTLIACADIS**FRGE

3 4

YIKYEETWRHSGVCA**FAGFLSTFSCQSSTLLLTLVTW**DRLMSVTRPLKPRDTEKVR**IVLRLL**

5

**LLWGISFGLAAAPLL**PNPYFGSHFYGNNGVCLSLHIHDPYAKGWEYS**ALLFILVNTLSLIFI**

6

**LFSYIRML**QAIRDSGGGMRSTHSGRENV**VATRFAIIVTTDCACWLPIIVVKL**AALSGCEISP

7

D**LYAWLAVLVLPVNSALNPVLYTLT**
