# Supplementary material for: Receptor deorphanization in starfish reveals the evolution of relaxin signaling as a regulator of reproduction
Source: BMC Biol. 2025 Feb 25;23:59. doi: 10.1186/s12915-025-02158-2 (PMC11863921; doi:10.1186/s12915-025-02158-2)
Supplement: Supplementary file 16 — Additional file 16. Dataset S9. Sequence data for the A. cf solaris proteins tested as receptors for AsolRGP1 and AsolRGP2, as shown in Fig. 5. [file 12915_2025_2158_MOESM16_ESM.docx]

***A. solaris* cf RXFP/LGR3**

**Signal peptide**

MGLVLLLALVFILVTVTANQVPLADHEGSIQAGPAQTEYEELSGDPSEFT **50**

▼ **LDLa Module**

**C**PRGEFQ**C**G**N**MTR**C**VAQKFQ**C**NGEDD**C**GNNADETE**C**EHDEGWIKNFDKQV **100**

**LRR-NT** ▼ ▼

PVSVQPERRISQECGLYGF**PEVCKCYETTNLRCLQGNLTEVPQDVSNN**LT **150**

**LRR1 LRR2**

HLNLNGNMLKNLEDGAF**E**R**YTKLRYLNLMGNGIRELPRDVFRG**LMDLDKL **200**

**LRR3** ▼ **LRR4**

FLSSNKISSLKPGTFRF**LRNLTWLFLNDNEIEVLDEEVFQG**LETVYWLML **250**

**LRR5 LRR6** ▼

QENRIRNLKRGISFRD**LPALMWLDISDSPLNHLSPDNFSL**SGNPPLSILT **300**

▼  **LRR7 LRR8** ▼

MNNC**N**ISTIHGDTLQQ**FRDLSTLHLSENKIQHFPSGLFRN**MI**N**LTDLAIA **350**

**LRR9 LRR10** ▼

**NNLATSLPEDLFDD**LVSLDVLNLGGLVIK**N**ISTRMFKGLTNLQHIEFSKF **400**

**TM1**

AYCRYAAHVRTCKPKSDGISSFRNLLKDGILRVSVWTIALLCFVGNVGVL **450**

**TM2**

VSRCLMKAENRIHSLVVMNLCTADFCMSIYLFIIGYHDAKFRNQFNTFAL **500**

**TM3**

EWMQSSTCKFAGFLAMFSSEVSVFMLTFISLERFICIVYPYRLHRLTSRE **550**

**TM4**

AIVIMTIIWFLGALVAWVPLVNVGYFVDFYGSNGVCFPLHIHDPWLQGWE **600**

**TM5**

YSAFIFLGLNASCFTAIAISYTAMFISIQQTRKATTHIGRRGDMNYAKRF **650**

**TM6 TM7**

FFVVLTDALCWLPIAILKILSLCSYQIPATLYGWIVIFVLPINSALNPIL **700**

YTLSTTSFSQWFHKHIKRRKGKSSNGSVGSKNEFSCSRGRIGSATDEPTE **750**

YSSVPKRSYDSDSSPVAEEKQCSTV **775**

**LRR – Leucine rich repeat**

**LRR-NT – Leucine rich repeat N-terminal domain**

**TM – Transmembrane domain**

▼ **– Potential N-linked glycosylation site**

NCBI; **E** – G (168), also 2017 annotation has this difference (old files)

**Interpro domain predictions**

LRRs; 149-169; 170-193; 194-217; 218-241; 242-265; 267-270; 317-340; 341-364; 365-388

TM Domains; 429-451; 464-486; 510-532; 552-571; 603-625; 650-672; 682-704

**NCBI conserved domain search also identifies LRR 7; 296-316**

***A. solaris* cf LGR4(long)**

MRKLHNALRMLFVLHLCASRRAEQDGAIIRHIQRRAVDAHKH**C**GKDFP**C**L **50**

▼ **LDLa Module 1** ▼

**N**STQ**C**VPQDSI**C**DGTPD**C**D**N**GSDEWEVEE**C**RDWNLAKMWDNFFGTKNDDS **100**

▼ **LDLa Module 2**

GEEEEDDLDQGLRPQQTVFDAHGH**C**GEEFP**C**M**N**STQ**C**VPREAI**C**NGKPD**C** **150**

▼

D**N**GSDEEEIV**C**RNKKMRDNIEDIRQEIPCEEGTFPESCDCFVEIEQTRTA **200**

▼ ▼

ELPSLASPY**N**TTGDASTYRDAYDIDS**N**STDGVRYGSEVRGVVIGIRLDCR **250**

▼ **LRR1 LRR2**

AKQLTNVPRNLP**N**NTISLDLSDNKITHLTKKDLTN**LPQLRQLLLSRNKLK** **300**

**LRR3 LRR4**

**RMEEGAFKP**LTDLETLRMIACDLEDVQQRMFAAQ**KRLVTLDLRYNKLTRL** **350**

**LRR5 LRR6**

**VKNSLFG**LRNVKYFDIRGNQLSEIETGVFED**TPKLYFMLVSQNKLSSIPA**  **400**

**LRR7 LRR8**

**NLLRP**LRELRTLDVHRNDISVIETGAFSTN**TKLIELNLRDNKLTEIRRGI** **450**

▼ **LRR9 LRR10**

**FHS**LTSTITLSLS**N**NSIRHLEQDAFAG**MNNLQTLKLTKNPFTSLPVGIFD** **500**

**Q**LISLKAIYFDHFSLCGYAPHVRLCMPKSDGISTAENLLGNILLRFAVWF **550**

**TM1 TM2**

VALLASLGNAFVLLARCFVNEDKKTHSFFIMNLAVADLLMGLYLLIIGIH **600**

▼ **TM3**

DVIFRGSYILHDLTWR**N**SSVCKLSGFLSLLSSEVSIMTLTVITLDRFLSI **650**

**TM4**

VHPFRFKNRSLVHARLLMVFLWLLGIALATIPLLHTAYFGEFYYGGNGVC **700**

**TM5**

LPLQIDQPFADGWEFSLVIFVVFNLVAFTFISYAYLMMFMTIRRSNLAMR **750**

**TM6**

STKKNQDWALVKRFTLIVATDFVCWMPIIIVKFVALGGVSVSQSVYAWFA **800**

**TM7**

IFVLPINSALNPILYTMTTVLFRQKILAPLGIVKAKRKKGYITGVSVDET **850**

STMSKNSGTRLSIISNKSRGGSLNGRFNSQK**KLKNLSSLDSTDESVTCSA 900 (881)**

**AQTTSLKIKKHRAATADYHELPTSDPDCAPSGVNDDME 938**

**LRR – Leucine rich repeat**

**LRR-NT – Leucine rich repeat N-terminal domain**

**TM – Transmembrane domain**

▼ **– Potential N-linked glycosylation site**

**Long transcript version**

**Interpro domain predictions**

LRRs; 261-285; 286-309; 310-333; 335-357; 358-381; 382-405; 406-429; 431-453; 454-477; 478-501

TM Domains; 546-565; 578-600; 623-645; 666-688; 717-739; 759-781; 796-815

**N-terminally modified and codon optimized AsolRXFP/LGR3 sequence with the methionine start codon underlined, the bovine prolactin signal sequence in red and the FLAG sequence in blue.**

**atggacagcaagggcagcagccagaagggcagcagactgctgctgctgctggtggtgagc**

**M  D  S  K  G  S  S  Q  K  G  S  R  L  L  L  L  L  V  V  S**

**aacctgctgctgtgccagggcgtggtgagcgactacaaggacgacgacgacgtg**aaccag

**N  L  L  L  C  Q  G  V  V  S  D  Y  K  D  D  D  D  V**  N  Q

gtgcccctggccgaccacgagggcagcatccaggccggccccgcccagaccgagtacgag

 V  P  L  A  D  H  E  G  S  I  Q  A  G  P  A  Q  T  E  Y  E

gagctgagcggcgaccccagcgagttcacctgccccagaggcgagttccagtgcggcaac

 E  L  S  G  D  P  S  E  F  T  **C** P  R  G  E  F  Q  **C**  G  N

atgaccagatgcgtggcccagaagttccagtgcaacggcgaggacgactgcggcaacaac

 M  T  R  **C**  V  A  Q  K  F  Q  **C**  N  G  E  D  D  **C**  G  N  N

gccgacgagaccgagtgcgagcacgacgagggctggatcaagaacttcgacaagcaggtg

 A  D  E  T  E  **C**  E  H  D  E  G  W  I  K  N  F  D  K  Q  V

cccgtgagcgtgcagcccgagagaagaatcagccaggagtgcggcctgtacggcttcccc

 P  V  S  V  Q  P  E  R  R  I  S  Q  E  C  G  L  Y  G  F  P

gaggtgtgcaagtgctacgagaccaccaacctgagatgcctgcagggcaacctgaccgag

 E  V  C  K  C  Y  E  T  T  N  L  R  C  L  Q  G  N  L  T  E

gtgccccaggacgtgagcaacaacctgacccacctgaacctgaacggcaacatgctgaag

 V  P  Q  D  V  S  N  N  L  T  H  L  N  L  N  G  N  M  L  K

aacctggaggacggcgccttcgagagatacaccaagctgagatacctgaacctgatgggc

 N  L  E  D  G  A  F  E  R  Y  T  K  L  R  Y  L  N  L  M  G

aacggcatcagagagctgcccagagacgtgttcagaggcctgatggacctggacaagctg

 N  G  I  R  E  L  P  R  D  V  F  R  G  L  M  D  L  D  K  L

ttcctgagcagcaacaagatcagcagcctgaagcccggcaccttcagattcctgagaaac

 F  L  S  S  N  K  I  S  S  L  K  P  G  T  F  R  F  L  R  N

ctgacctggctgttcctgaacgacaacgagatcgaggtgctggacgaggaggtgttccag

 L  T  W  L  F  L  N  D  N  E  I  E  V  L  D  E  E  V  F  Q

ggcctggagaccgtgtactggctgatgctgcaggagaacagaatcagaaacctgaagaga

 G  L  E  T  V  Y  W  L  M  L  Q  E  N  R  I  R  N  L  K  R

ggcatcagcttcagagacctgcccgccctgatgtggctggacatcagcgacagccccctg

 G  I  S  F  R  D  L  P  A  L  M  W  L  D  I  S  D  S  P  L

aaccacctgagccccgacaacttcagcctgagcggcaacccccccctgagcatcctgacc

 N  H  L  S  P  D  N  F  S  L  S  G  N  P  P  L  S  I  L  T

atgaacaactgcaacatcagcaccatccacggcgacaccctgcagcagttcagagacctg

 M  N  N  C  N  I  S  T  I  H  G  D  T  L  Q  Q  F  R  D  L

agcaccctgcacctgagcgagaacaagatccagcacttccccagcggcctgttcagaaac

 S  T  L  H  L  S  E  N  K  I  Q  H  F  P  S  G  L  F  R  N

atgatcaacctgaccgacctggccatcgccaacaacctggccaccagcctgcccgaggac

 M  I  N  L  T  D  L  A  I  A  N  N  L  A  T  S  L  P  E  D

ctgttcgacgacctggtgagcctggacgtgctgaacctgggcggcctggtgatcaagaac

 L  F  D  D  L  V  S  L  D  V  L  N  L  G  G  L  V  I  K  N

atcagcaccagaatgttcaagggcctgaccaacctgcagcacatcgagttcagcaagttc

 I  S  T  R  M  F  K  G  L  T  N  L  Q  H  I  E  F  S  K  F

gcctactgcagatacgccgcccacgtgagaacctgcaagcccaagagcgacggcatcagc

 A  Y  C  R  Y  A  A  H  V  R  T  C  K  P  K  S  D  G  I  S

agcttcagaaacctgctgaaggacggcatcctgagagtgagcgtgtggaccatcgccctg

 S  F  R  N  L  L  K  D  G  I  L  R  V  S  V  W  T  I  A  L

ctgtgcttcgtgggcaacgtgggcgtgctggtgagcagatgcctgatgaaggccgagaac

 L  C  F  V  G  N  V  G  V  L  V  S  R  C  L  M  K  A  E  N

agaatccacagcctggtggtgatgaacctgtgcaccgccgacttctgcatgagcatctac

 R  I  H  S  L  V  V  M  N  L  C  T  A  D  F  C  M  S  I  Y

ctgttcatcatcggctaccacgacgccaagttcagaaaccagttcaacaccttcgccctg

 L  F  I  I  G  Y  H  D  A  K  F  R  N  Q  F  N  T  F  A  L

gagtggatgcagagcagcacctgcaagttcgccggcttcctggccatgttcagcagcgag

 E  W  M  Q  S  S  T  C  K  F  A  G  F  L  A  M  F  S  S  E

gtgagcgtgttcatgctgaccttcatcagcctggagagattcatctgcatcgtgtacccc

 V  S  V  F  M  L  T  F  I  S  L  E  R  F  I  C  I  V  Y  P

tacagactgcacagactgaccagcagagaggccatcgtgatcatgaccatcatctggttc

 Y  R  L  H  R  L  T  S  R  E  A  I  V  I  M  T  I  I  W  F

ctgggcgccctggtggcctgggtgcccctggtgaacgtgggctacttcgtggacttctac

 L  G  A  L  V  A  W  V  P  L  V  N  V  G  Y  F  V  D  F  Y

ggcagcaacggcgtgtgcttccccctgcacatccacgacccctggctgcagggctgggag

 G  S  N  G  V  C  F  P  L  H  I  H  D  P  W  L  Q  G  W  E

tacagcgccttcatcttcctgggcctgaacgccagctgcttcaccgccatcgccatcagc

 Y  S  A  F  I  F  L  G  L  N  A  S  C  F  T  A  I  A  I  S

tacaccgccatgttcatcagcatccagcagaccagaaaggccaccacccacatcggcaga

 Y  T  A  M  F  I  S  I  Q  Q  T  R  K  A  T  T  H  I  G  R

agaggcgacatgaactacgccaagagattcttcttcgtggtgctgaccgacgccctgtgc

 R  G  D  M  N  Y  A  K  R  F  F  F  V  V  L  T  D  A  L  C

tggctgcccatcgccatcctgaagatcctgagcctgtgcagctaccagatccccgccacc

 W  L  P  I  A  I  L  K  I  L  S  L  C  S  Y  Q  I  P  A  T

ctgtacggctggatcgtgatcttcgtgctgcccatcaacagcgccctgaaccccatcctg

 L  Y  G  W  I  V  I  F  V  L  P  I  N  S  A  L  N  P  I  L

tacaccctgagcaccaccagcttcagccagtggttccacaagcacatcaagagaagaaag

 Y  T  L  S  T  T  S  F  S  Q  W  F  H  K  H  I  K  R  R  K

ggcaagagcagcaacggcagcgtgggcagcaagaacgagttcagctgcagcagaggcaga

 G  K  S  S  N  G  S  V  G  S  K  N  E  F  S  C  S  R  G  R

atcggcagcgccaccgacgagcccaccgagtacagcagcgtgcccaagagaagctacgac

 I  G  S  A  T  D  E  P  T  E  Y  S  S  V  P  K  R  S  Y  D

agcgacagcagccccgtggccgaggagaagcagtgcagcaccgtgtga

 S  D  S  S  P  V  A  E  E  K  Q  C  S  T  V  -

**N-terminally modified and codon optimized AsolLGR4(short) sequence with the methionine start codon underlined, the bovine prolactin signal sequence in red and the FLAG sequence in blue.**

**atggacagcaagggcagcagccagaagggcagcagactgctgctgctgctggtggtgagc**

**M D S K G S S Q K G S R L L L L L V V S**

**aacctgctgctgtgccagggcgtggtgagcgactacaaggacgacgacgacgtg**gagcag

**N L L L C Q G V V S** **D Y K D D D D V** E Q

gacggcgccatcatcagacacatccagagaagagccgtggacgcccacaagcactgcggc

D G A I I R H I Q R R A V D A H K H C G

aaggacttcccctgcctgaacagcacccagtgcgtgccccaggacagcatctgcgacggc

K D F P C L N S T Q C V P Q D S I C D G

acccccgactgcgacaacggcagcgacgagtgggaggtggaggagtgcagagactggaac

T P D C D N G S D E W E V E E C R D W N

ctggccaagatgtgggacaacttcttcggcaccaagaacgacgacagcggcgaggaggag

L A K M W D N F F G T K N D D S G E E E

gaggacgacctggaccagggcctgagaccccagcagaccgtgttcgacgcccacggccac

E D D L D Q G L R P Q Q T V F D A H G H

tgcggcgaggagttcccctgcatgaacagcacccagtgcgtgcccagagaggccatctgc

C G E E F P C M N S T Q C V P R E A I C

aacggcaagcccgactgcgacaacggcagcgacgaggaggagatcgtgtgcagaaacaag

N G K P D C D N G S D E E E I V C R N K

aagatgagagacaacatcgaggacatcagacaggagatcccctgcgaggagggcaccttc

K M R D N I E D I R Q E I P C E E G T F

cccgagagctgcgactgcttcgtggagatcgagcagaccagaaccgccgagctgcccagc

P E S C D C F V E I E Q T R T A E L P S

ctggccagcccctacaacaccaccggcgacgccagcacctacagagacgcctacgacatc

L A S P Y N T T G D A S T Y R D A Y D I

gacagcaacagcaccgacggcgtgagatacggcagcgaggtgagaggcgtggtgatcggc

D S N S T D G V R Y G S E V R G V V I G

atcagactggactgcagagccaagcagctgaccaacgtgcccagaaacctgcccaacaac

I R L D C R A K Q L T N V P R N L P N N

accatcagcctggacctgagcgacaacaagatcacccacctgaccaagaaggacctgacc

T I S L D L S D N K I T H L T K K D L T

aacctgccccagctgagacagctgctgctgagcagaaacaagctgaagagaatggaggag

N L P Q L R Q L L L S R N K L K R M E E

ggcgccttcaagcccctgaccgacctggagaccctgagaatgatcgcctgcgacctggag

G A F K P L T D L E T L R M I A C D L E

gacgtgcagcagagaatgttcgccgcccagaagagactggtgaccctggacctgagatac

D V Q Q R M F A A Q K R L V T L D L R Y

aacaagctgaccagactggtgaagaacagcctgttcggcctgagaaacgtgaagtacttc

N K L T R L V K N S L F G L R N V K Y F

gacatcagaggcaaccagctgagcgagatcgagaccggcgtgttcgaggacacccccaag

D I R G N Q L S E I E T G V F E D T P K

ctgtacttcatgctggtgagccagaacaagctgagcagcatccccgccaacctgctgaga

L Y F M L V S Q N K L S S I P A N L L R

cccctgagagagctgagaaccctggacgtgcacagaaacgacatcagcgtgatcgagacc

P L R E L R T L D V H R N D I S V I E T

ggcgccttcagcaccaacaccaagctgatcgagctgaacctgagagacaacaagctgacc

G A F S T N T K L I E L N L R D N K L T

gagatcagaagaggcatcttccacagcctgaccagcaccatcaccctgagcctgagcaac

E I R R G I F H S L T S T I T L S L S N

aacagcatcagacacctggagcaggacgccttcgccggcatgaacaacctgcagaccctg

N S I R H L E Q D A F A G M N N L Q T L

aagctgaccaagaaccccttcaccagcctgcccgtgggcatcttcgaccagctgatcagc

K L T K N P F T S L P V G I F D Q L I S

ctgaaggccatctacttcgaccacttcagcctgtgcggctacgccccccacgtgagactg

L K A I Y F D H F S L C G Y A P H V R L

tgcatgcccaagagcgacggcatcagcaccgccgagaacctgctgggcaacatcctgctg

C M P K S D G I S T A E N L L G N I L L

agattcgccgtgtggttcgtggccctgctggccagcctgggcaacgccttcgtgctgctg

R F A V W F V A L L A S L G N A F V L L

gccagatgcttcgtgaacgaggacaagaagacccacagcttcttcatcatgaacctggcc

A R C F V N E D K K T H S F F I M N L A

gtggccgacctgctgatgggcctgtacctgctgatcatcggcatccacgacgtgatcttc

V A D L L M G L Y L L I I G I H D V I F

agaggcagctacatcctgcacgacctgacctggagaaacagcagcgtgtgcaagctgagc

R G S Y I L H D L T W R N S S V C K L S

ggcttcctgagcctgctgagcagcgaggtgagcatcatgaccctgaccgtgatcaccctg

G F L S L L S S E V S I M T L T V I T L

gacagattcctgagcatcgtgcaccccttcagattcaagaacagaagcctggtgcacgcc

D R F L S I V H P F R F K N R S L V H A

agactgctgatggtgttcctgtggctgctgggcatcgccctggccaccatccccctgctg

R L L M V F L W L L G I A L A T I P L L

cacaccgcctacttcggcgagttctactacggcggcaacggcgtgtgcctgcccctgcag

H T A Y F G E F Y Y G G N G V C L P L Q

atcgaccagcccttcgccgacggctgggagttcagcctggtgatcttcgtggtgttcaac

I D Q P F A D G W E F S L V I F V V F N

ctggtggccttcaccttcatcagctacgcctacctgatgatgttcatgaccatcagaaga

L V A F T F I S Y A Y L M M F M T I R R

agcaacctggccatgagaagcaccaagaagaaccaggactgggccctggtgaagagattc

S N L A M R S T K K N Q D W A L V K R F

accctgatcgtggccaccgacttcgtgtgctggatgcccatcatcatcgtgaagttcgtg

T L I V A T D F V C W M P I I I V K F V

gccctgggcggcgtgagcgtgagccagagcgtgtacgcctggttcgccatcttcgtgctg

A L G G V S V S Q S V Y A W F A I F V L

cccatcaacagcgccctgaaccccatcctgtacaccatgaccaccgtgctgttcagacag

P I N S A L N P I L Y T M T T V L F R Q

aagatcctggcccccctgggcatcgtgaaggccaagagaaagaagggctacatcaccggc

K I L A P L G I V K A K R K K G Y I T G

gtgagcgtggacgagaccagcaccatgagcaagaacagcggcaccagactgagcatcatc

V S V D E T S T M S K N S G T R L S I I

agcaacaagagcagaggcggcagcctgaacggcagattcaacagccagaagtga

S N K S R G G S L N G R F N S Q K -

**N-terminally modified and codon optimized AsolLGR4(long) sequence with the methionine start codon underlined, the bovine prolactin signal sequence in red and the FLAG sequence in blue.**

**atggacagcaagggcagcagccagaagggcagcagactgctgctgctgctggtggtgagc**

**M D S K G S S Q K G S R L L L L L V V S**

**aacctgctgctgtgccagggcgtggtgagcgactacaaggacgacgacgacgtg**gagcag

**N L L L C Q G V V S** **D Y K D D D D V** E Q

gacggcgccatcatcagacacatccagagaagagccgtggacgcccacaagcactgcggc

D G A I I R H I Q R R A V D A H K H C G

aaggacttcccctgcctgaacagcacccagtgcgtgccccaggacagcatctgcgacggc

K D F P C L N S T Q C V P Q D S I C D G

acccccgactgcgacaacggcagcgacgagtgggaggtggaggagtgcagagactggaac

T P D C D N G S D E W E V E E C R D W N

ctggccaagatgtgggacaacttcttcggcaccaagaacgacgacagcggcgaggaggag

L A K M W D N F F G T K N D D S G E E E

gaggacgacctggaccagggcctgagaccccagcagaccgtgttcgacgcccacggccac

E D D L D Q G L R P Q Q T V F D A H G H

tgcggcgaggagttcccctgcatgaacagcacccagtgcgtgcccagagaggccatctgc

C G E E F P C M N S T Q C V P R E A I C

aacggcaagcccgactgcgacaacggcagcgacgaggaggagatcgtgtgcagaaacaag

N G K P D C D N G S D E E E I V C R N K

aagatgagagacaacatcgaggacatcagacaggagatcccctgcgaggagggcaccttc

K M R D N I E D I R Q E I P C E E G T F

cccgagagctgcgactgcttcgtggagatcgagcagaccagaaccgccgagctgcccagc

P E S C D C F V E I E Q T R T A E L P S

ctggccagcccctacaacaccaccggcgacgccagcacctacagagacgcctacgacatc

L A S P Y N T T G D A S T Y R D A Y D I

gacagcaacagcaccgacggcgtgagatacggcagcgaggtgagaggcgtggtgatcggc

D S N S T D G V R Y G S E V R G V V I G

atcagactggactgcagagccaagcagctgaccaacgtgcccagaaacctgcccaacaac

I R L D C R A K Q L T N V P R N L P N N

accatcagcctggacctgagcgacaacaagatcacccacctgaccaagaaggacctgacc

T I S L D L S D N K I T H L T K K D L T

aacctgccccagctgagacagctgctgctgagcagaaacaagctgaagagaatggaggag

N L P Q L R Q L L L S R N K L K R M E E

ggcgccttcaagcccctgaccgacctggagaccctgagaatgatcgcctgcgacctggag

G A F K P L T D L E T L R M I A C D L E

gacgtgcagcagagaatgttcgccgcccagaagagactggtgaccctggacctgagatac

D V Q Q R M F A A Q K R L V T L D L R Y

aacaagctgaccagactggtgaagaacagcctgttcggcctgagaaacgtgaagtacttc

N K L T R L V K N S L F G L R N V K Y F

gacatcagaggcaaccagctgagcgagatcgagaccggcgtgttcgaggacacccccaag

D I R G N Q L S E I E T G V F E D T P K

ctgtacttcatgctggtgagccagaacaagctgagcagcatccccgccaacctgctgaga

L Y F M L V S Q N K L S S I P A N L L R

cccctgagagagctgagaaccctggacgtgcacagaaacgacatcagcgtgatcgagacc

P L R E L R T L D V H R N D I S V I E T

ggcgccttcagcaccaacaccaagctgatcgagctgaacctgagagacaacaagctgacc

G A F S T N T K L I E L N L R D N K L T

gagatcagaagaggcatcttccacagcctgaccagcaccatcaccctgagcctgagcaac

E I R R G I F H S L T S T I T L S L S N

aacagcatcagacacctggagcaggacgccttcgccggcatgaacaacctgcagaccctg

N S I R H L E Q D A F A G M N N L Q T L

aagctgaccaagaaccccttcaccagcctgcccgtgggcatcttcgaccagctgatcagc

K L T K N P F T S L P V G I F D Q L I S

ctgaaggccatctacttcgaccacttcagcctgtgcggctacgccccccacgtgagactg

L K A I Y F D H F S L C G Y A P H V R L

tgcatgcccaagagcgacggcatcagcaccgccgagaacctgctgggcaacatcctgctg

C M P K S D G I S T A E N L L G N I L L

agattcgccgtgtggttcgtggccctgctggccagcctgggcaacgccttcgtgctgctg

R F A V W F V A L L A S L G N A F V L L

gccagatgcttcgtgaacgaggacaagaagacccacagcttcttcatcatgaacctggcc

A R C F V N E D K K T H S F F I M N L A

gtggccgacctgctgatgggcctgtacctgctgatcatcggcatccacgacgtgatcttc

V A D L L M G L Y L L I I G I H D V I F

agaggcagctacatcctgcacgacctgacctggagaaacagcagcgtgtgcaagctgagc

R G S Y I L H D L T W R N S S V C K L S

ggcttcctgagcctgctgagcagcgaggtgagcatcatgaccctgaccgtgatcaccctg

G F L S L L S S E V S I M T L T V I T L

gacagattcctgagcatcgtgcaccccttcagattcaagaacagaagcctggtgcacgcc

D R F L S I V H P F R F K N R S L V H A

agactgctgatggtgttcctgtggctgctgggcatcgccctggccaccatccccctgctg

R L L M V F L W L L G I A L A T I P L L

cacaccgcctacttcggcgagttctactacggcggcaacggcgtgtgcctgcccctgcag

H T A Y F G E F Y Y G G N G V C L P L Q

atcgaccagcccttcgccgacggctgggagttcagcctggtgatcttcgtggtgttcaac

I D Q P F A D G W E F S L V I F V V F N

ctggtggccttcaccttcatcagctacgcctacctgatgatgttcatgaccatcagaaga

L V A F T F I S Y A Y L M M F M T I R R

agcaacctggccatgagaagcaccaagaagaaccaggactgggccctggtgaagagattc

S N L A M R S T K K N Q D W A L V K R F

accctgatcgtggccaccgacttcgtgtgctggatgcccatcatcatcgtgaagttcgtg

T L I V A T D F V C W M P I I I V K F V

gccctgggcggcgtgagcgtgagccagagcgtgtacgcctggttcgccatcttcgtgctg

A L G G V S V S Q S V Y A W F A I F V L

cccatcaacagcgccctgaaccccatcctgtacaccatgaccaccgtgctgttcagacag

P I N S A L N P I L Y T M T T V L F R Q

aagatcctggcccccctgggcatcgtgaaggccaagagaaagaagggctacatcaccggc

K I L A P L G I V K A K R K K G Y I T G

gtgagcgtggacgagaccagcaccatgagcaagaacagcggcaccagactgagcatcatc

V S V D E T S T M S K N S G T R L S I I

agcaacaagagcagaggcggcagcctgaacggcagattcaacagccagaagaagctgaag

S N K S R G G S L N G R F N S Q K K L K

aacctgagcagcctggacagcaccgacgagagcgtgacctgcagcgccgcccagaccacc

N L S S L D S T D E S V T C S A A Q T T

agcctgaagatcaagaagcacagagccgccaccgccgactaccacgagctgcccaccagc

S L K I K K H R A A T A D Y H E L P T S

gaccccgactgcgcccccagcggcgtgaacgacgacatggagtga

D P D C A P S G V N D D M E -
