# Supplementary material for: Receptor deorphanization in starfish reveals the evolution of relaxin signaling as a regulator of reproduction
Source: BMC Biol. 2025 Feb 25;23:59. doi: 10.1186/s12915-025-02158-2 (PMC11863921; doi:10.1186/s12915-025-02158-2)
Supplement: Supplementary file 18 — Additional file 18. Dataset S11. Sequence data for the A. rubens proteins tested as receptors for ArubRGP1 and ArubRGP2, as shown in Fig. 6. [file 12915_2025_2158_MOESM18_ESM.docx]

ctcaga…: genome sequence

ctcaga…: transcriptome sequence

ga: position of intron

VIF: signal peptide

TCP: LDLa domain

N: N-glyco motif

VS: Transmembrane domain

D   Y   K   D   D   D   D   K: FLAG epitope

**Original *A.rubens* RXFP/LGR3: Scaffold31462**

ct

ct

cagattgaaattaatagccgaagcatttatccattctctttttggatagggctgttcaca

cagattgaaattaatagccgaagcatttatccattctctttttggatagggctgttcaca

ctcagacaaaacgtcagatcaataactagaaggtaacagttgtagacgtaatgcttgtgc

ctcagacaaaacgtcagatcaataactagaaggtaacagttgtagacgtaatgcttgtgt

ggacggcagtttggtaagatttacgtgatcagctgaggtgaacgtcgagtggagtcttca

ggacggcagtttggtaagatttacgtgatcagctgaggtgaacgtcgagtggagtcttca

acggggaagattcgtcttctcccaggggagcagggagcgagtaaagcgcttcaatcctcg

acggggaagattcgtcttctcccaggggagcagggagcgagtaaagcgcttcaatcctcg

gggatatttcgttcgtgtgaaagagagaaatttatctacaactgagtgtcagaatgtgtg

gggatatttcgttcgtgtgaaagagagaaatttatctacaactgagtgtcagaatgtgtg

actgcagtcatcgtagacaattggccgtcgatagtactttatggggaatgtgatgtgggc

actgcagtcatcgtagacaattggccgtcgatagtactttatggggaatgtgatgtgggc

tggtgttagggttgttcataccacacgacgggcactgcagactgttggcttgtgagacta

tggtgttagggttgttcataccacacgacgggcactgcagactgttggcttgtgagacta

cgtacgctccgcttgtcgagaaattgctggtagaaactgtgatcgagcagtcagagtaaa

cgtacgctccgcttgtcgagaaattgctgntagaaactgtgatcgagcagtcagagtaaa

ttgatgggcttcattttccacttttacgtcttgaactccgactctgctgattcttcactt

ttgatgggcttcattttccacttttacgtcttgaactccgactctgctgattcttcactt

aatggaaaggacacctcagacacctgcaactaatggaaaggaaatttcaacgagaccatt

aatggaaaggacacctcagacacctgcaactaatggaaaggaaatttcaacgagaccatt

ggatgcagacttcaactaattgaaaactttgacagatttcagtctcgataccccacttcg

ggatgcagacttcaactaattgaaaactttgacagatttcagtctcgataccccacttcg

gcaggttcaatcctcgcaacagtttggttctcttccgatagtgtcgaaaacggtgttgat

gcaggttcaatcctcgcaacagtttggttctcttccgatagtgtcgaaaacggtgttgat

tgttgtcaatcgtgcaactgccgtggagtttttgttgttgtaatggagattaaagaaaag

tgttgtcaatcgtgcaactgccgtggagtttttgttgttgtaatggagattaaagaaaag

M  E  I  K  E  K  6

gactccgaaaacaaacagatggacctgtctttgaataatgatgcaaagaaggcaccgctc

gactccgaaaacaaacagatggacctgtctttgaataatgatgcaaagaaggcaccgctc

D  S  E  N  K  Q  M  D  L  S  L  N  N  D  A  K  K  A  P  L  26

cggctacccttagagtctcgtaacatgaacagcagatattcccgatgtaattcattccaa

cggctacccttagagtctcgtaacatgaacagcagatattcccgatgtaattcattccaa

R  L  P  L  E  S  R  N  M  N  S  R  Y  S  R  C  N  S  F  Q  46

gccgagtgtcgttctctgggtaaacgatgtaagctggcgttggtggcagttgttgttata

gccgagtgtcgttctctgggtaaacgatgtaagctggcgttggtggcagttgttgttata

 A  E  C  R  S  L  G  K  R  C  K  L  A  L  V  A  V  V  V  I  66

ttcctcttggtcatcataattgctgtccttctaacagttttaggtagcgggagggtccag

ttcctcttggtcatcataattgctgtccttctaacagttttaggtagcgggagggtccag

F  L  L  V  I  I  I  A  V  L  L  T  V  L  G  S  G  R  V  Q  86

ccatcagaggacgatgcttatgctgtgacagtgtcagctgaatatgaaatgaatgggaac

ccatcagaggacgatgcttatgctgtgacagtgtcagctgaatatgaaatgaatgggaac

P  S  E  D  D  A  Y  A  V  T  V  S  A  E  Y  E  M  N  G  N  106

gttggagacttcacctgcccgcaaggagaatttcgatgcaacaatcttactcgctgtgtt

gttggagacttcacctgcccgcaaggagaatttcgatgcaacaatcttactcgctgtgtt

V  G  D  F  T  C  P  Q  G  E  F  R  C  N  N  L  T  R  C  V  126

gcccaacaattccagtgcaacaaaatcgatgattgtggaaacaatgctgacgaaatggaa

gcccaacaattccagtgcaacaaaatcgatgattgtggaaacaatgctgacgaaatggaa

A  Q  Q  F  Q  C  N  K  I  D  D  C  G  N  N  A  D  E  M  E  146

tgtgaacacgacgaaggctggataaaaacttttgacgagaagattccgatacgggtgcag

tgtgaacacgacgaaggctggataaaaacttttgacgagaagattccgatacgggtgcag

C  E  H  D  E  G  W  I  K  T  F  D  E  K  I  P  I  R  V  Q  166

ccgaaacgggagccaagcacagaatgcagcctgtcggggttcccggatgtatgtatttgc

ccgaaacgggagccaagcacagaatgcagcctgtcggggttcccggatgtatgtatttgc

 P  K  R  E  P  S  T  E  C  S  L  S  G  F  P  D  V  C  I  C  186

tacgagaacaccagcttaaagtgtgtatacgcaaacctgacaaaggtacctccgggtata

tacgagaacaccagcttaaagtgtgtatacgcaaacctgacaaaagtacctccgggtata

Y  E  N  T  S  L  K  C  V  Y  A  N  L  T  K  V  P  P  G  I  206

tctagcaacatcacccatctcaatctcagaggcaatcagattatttttgaggatggagtc

tctagcaacatcacccatctcaatctcagaggcaatcagattatttttgaggatggagtc

S  S  N  I  T  H  L  N  L  R  G  N  Q  I  I  F  E  D  G  V  226

tttgagggttactcgaatctacaatcgttaaacctgatggacaatggcataatcgaactc

tttgagggttactcgaatctacaatcgttaaacctgatggacaatggcataatcgaactc

F  E  G  Y  S  N  L  Q  S  L  N  L  M  D  N  G  I  I  E  L  246

cccgtcgatgtattccgaggattgcgcaatctggataaactatatctttcggacaacaaa

cccgtcgatgtattccgaggattgcgcaatctggataaactatatctttcggacaacaaa

P  V  D  V  F  R  G  L  R  N  L  D  K  L  Y  L  S  D  N  K  266

atcaagttcttaattccaggaacgttcagatatttgaacaacgtcacgtggctgttcatg

atcaagttcttaattccaggaacgttcagatatttgaacaacgtcacgtggctgttcatg

I  K  F  L  I  P  G  T  F  R  Y  L  N  N  V  T  W  L  F  M  286

agtaataacgaactagcagcattgggtgaaggggtcttccaagggatggataaactctac

agtaataacgaactagcagcattgggtgaaggggtcttccaagggatggataaactctac

S  N  N  E  L  A  A  L  G  E  G  V  F  Q  G  M  D  K  L  Y  306

tggctgatgttgccaaataatagcataagcagtttagctcgttccgtagcattccgtgat

tggctgatgttgccaaataatagcataagcagtttagctcgttccgtagcattccgtgat

  W  L  M  L  P  N  N  S  I  S  S  L  A  R  S  V  A  F  R  D  326

actccaagtttagtctggttcgatatatccgaaaatccactaggaaggctatctccgtat

actccaagtttagtctggttcgatatatccgaaaatccactaggaaggctatctccgtat

T  P  S  L  V  W  F  D  I  S  E  N  P  L  G  R  L  S  P  Y  346

aacttctctttacctggtaacgtctccataattagtctcttaacaatcaacaactgtaac

aacttctctttacctggtaacgtctccataattagtctcttaacaatcaacaactgtaac

N  F  S  L  P  G  N  V  S  I  I  S  L  L  T  I  N  N  C  N  366

ataagcagcattcatcgagacacatttcgatcattccgtggcctctcagtcctgcattta

ataagcagcattcatcgagacacatttcgatcattccgtggcctctcagtcctgcattta

I  S  S  I  H  R  D  T  F  R  S  F  R  G  L  S  V  L  H  L  386

tcgcacaattctataaagtatttcccgtcaggcttgtttaggaatctaagtaaacttgct

tcgcacaattctataaagtatttcccgtcaggcttgtttaggaatctaagtaaacttgct

 S  H  N  S  I  K  Y  F  P  S  G  L  F  R  N  L  S  K  L  A  406

gaccttcatatcgacaacaacccagcaacatctcttcctgaggatctcttcgatggactc

gaccttcannnnnnnnnnnnnnnnnnnnnnnnnnnnnnnnnngatctcttcgatggactc

D  L  H  I  D  N  N  P  A  T  S  L  P  E  D  L  F  D  G  L  426

caccgaatagacgttttggatttacaaggaatgacaattactaatataaattctcggatg

caccgaatagacgttttggatttacaaggaatgacaattactaatataaattctcggatg

H  R  I  D  V  L  D  L  Q  G  M  T  I  T  N  I  N  S  R  M  446

ttcgaggaactatcgacccttcagcatattgaattttccaagtttgactattgtcgttac

ttcgaggaactatcgacccttcagcatattgaattttccaagtttgactattgtcgttac

F  E  E  L  S  T  L  Q  H  I  E  F  S  K  F  D  Y  C  R  Y  466

gctccacacgttcgaacatgcaaaccaaggtctaatggtatctcgtcatttgaagaccta

gctccacacgttcgaacatgcaaaccaaggtctaatggtatctcgtcatttgaagaccta

A  P  H  V  R  T  C  K  P  R  S  N  G  I  S  S  F  E  D  L  486

ttgaaggatggtatactacgtgtatctgtatggactattgctctactttgtttctttggt

ttgaaggatggtatactacgtgtatctgtatggactattgctctactttgtttctttggt

 L  K  D  G  I  L  R  V  S  V  W  T  I  A  L  L  C  F  F  G  506

aatgttggtgttctcatcagtcgattcatgatgaaagccgagaatcgtattcactcacta

aatgttggtgttctcatcagtcgattcatgatgaaagccgagaatcgtattcactcacta

N  V  G  V  L  I  S  R  F  M  M  K  A  E  N  R  I  H  S  L  526

gtagtcatcaatctctgcactgctgatttcttcatgtccatttacctcatcattattggc

gtagtcatcaatctctgcactgctgatttcttcatgtccatttacctcatcattatcggc

V  V  I  N  L  C  T  A  D  F  F  M  S  I  Y  L  I  I  I  G  546

ttccatgatgtcaagtttcgtaatttcttcaacatgtatgctcttgagtggatgcaggga

ttccatgatgtcaagtttcgtaatttcttcaacatgtatgctcttgagtggatgcaggga

 F  H  D  V  K  F  R  N  F  F  N  M  Y  A  L  E  W  M  Q  G  566

tctacttgtaagttcgctggtttcctagccatgttctccagtgaggtatcagtattcatg

tctacttgtaagttcgctggtttcctagccatgttctccagtgaggtatcagtattcatg

 S  T  C  K  F  A  G  F  L  A  M  F  S  S  E  V  S  V  F  M  586

ctgacgttcatatccctggagcgcttcatttgtatcgtatacccttacaggcttcacaga

ctgactttcatatccctggagcgcttcatttgtatcgtatacccttacaggcttcacaga

L  T  F  I  S  L  E  R  F  I  C  I  V  Y  P  Y  R  L  H  R  606

ttgacaagcaaagaggccactgtagtgatgtcggttatctggttcttgggcgccctggta

ttgacaagcaaagaggccactgtagtgatgtcggttatctggttcttgggcgccctggta

L  T  S  K  E  A  T  V  V  M  S  V  I  W  F  L  G  A  L  V  626

gcatggattccgctcatcagtgtgggttattttgtcgacttctacggcagcaatggggtg

gcatggattccgctcatcagtgtgggttattttgtcgacttctacggcagcaatggggtg

A  W  I  P  L  I  S  V  G  Y  F  V  D  F  Y  G  S  N  G  V  646

tgtttcccgctccatattcacgacccgtggctaccaggatgggagtattcagcctttgta

tgtttcccgctccatattcacgacccgtggctaccaggatgggagtattcagcctttgta

C  F  P  L  H  I  H  D  P  W  L  P  G  W  E  Y  S  A  F  V  666

ttccttggactgaatgcgagctgcttcactgcaatcgccatttcttacactggaatgttc

ttccttggactgaatgcgagctgcttcactgcaatcgccatttcttacactggaatgttc

F  L  G  L  N  A  S  C  F  T  A  I  A  I  S  Y  T  G  M  F  686

atcagcatacagaggaccagaaaagctacgacgaatatcggcaaacgcggtgacatgaac

atcagcatacagaggaccagaaaagctacgacgaatatcggcaaacgcggtgacatgaac

 I  S  I  Q  R  T  R  K  A  T  T  N  I  G  K  R  G  D  M  N  706

tacgccaagcgtttccttttcgttgtgctgacagacgccctctgctggctgcccatcgcc

tacgccaagcgtttccttttcgttgtgctgacagacgccctctgctggctgcccatcgcc

 Y  A  K  R  F  L  F  V  V  L  T  D  A  L  C  W  L  P  I  A  726

attctgaaaattctctcactatgcagctataagattcctgcaaccttgtatggctggatc

attctgaaaattctctcactatgcagctataagattcctgcaaccttgtatggctggatc

 I  L  K  I  L  S  L  C  S  Y  K  I  P  A  T  L  Y  G  W  I  746

atcgtgtttgttttacccatcaatagcgccctcaacccaatcctatacacaatcagcaca

atcgtgtttgttttacccatcaatagcgccctcaacccaatcctatacacaatcagcaca

 I  V  F  V  L  P  I  N  S  A  L  N  P  I  L  Y  T  I  S  T  766

acctcgttctcacagtggttccacaaacacgttaagttgcgaaggcgaggcgagggtcgt

acctcgttctcacagtggttccacaaacacgttaagttgcgaaggcgaggcgagggtcgt

 T  S  F  S  Q  W  F  H  K  H  V  K  L  R  R  R  G  E  G  R  786

ggtagtcttagattcaagaacgaattctcgtccatgggagattttacatatggtatctca

ggtagtcttagattcaagaacgaattctcgtccatgggagattttacatatggtatctca

G  S  L  R  F  K  N  E  F  S  S  M  G  D  F  T  Y  G  I  S  806

gacattgaacataaaccaggagctatcgcggagtagcatctcagctttatgaaatgggtg

gacattgaacataaaccaggagctatcgcggagtagcatctcagctttatgaaatgggtg

D  I  E  H  K  P  G  A  I  A  E  **★** 817

tgcctaaagggcggttgaactccaataccgatcatccgactgagtatagtactgtaccga

tgcctaaagggcggttgaactccaataccgatcatccgactgagtatagtactgtaccga

agagatcatacgacagtaactcgtccccagtcgccgaagaacctcagtgcagctccgtct

agagatcatacgacagtaactcgtccccagtcgccgaagaacctcagtgcagctccgtct

gacagacgcaacgcgagacacaatgcgtcatcacggacgccaataactcgttggaaacat

gacagacgcaacacgagacacaatgcgtcatcacggacgccaataactcgttggaaacat

tggtatggtgtcccatgctgagtgagcagccaacagtcatcatatcctgaaatagtttgg

tggtatggtgtcccatgctgagtgagcagccaacagtcatcatatcctgagatagtttgg

gtatttcgactgatttacctattgggagacctgtgcaattgtacattcaacattcgtaat

gtattttgactgatttacctattgggagacctgtgcaattgtacattcaacattcgtaat

gtcacgcctaagttttcggctgtctgccaaaaaggcttttgccgcacaactataacaaga

gtcacgcctaagttttgggctgtctgccaaaaaggcttttgccgcacaactataacaaga

tagttgcttcagaaagatcggctgtaaaatcacagtgaaaaactcaatttaagattcagt

tagttgcttcagaaagatcggctgtaaaatcacagtgaaaaactcaatttaagattcagt

attcatgtttgattgatgttcatttgtaagacgtgttacatcgccaaatacttgagacca

attcatttttgattgatgttcatttgtaagacatgttacatcgccaaatacttgagagca

tggggaaattataaaaagaaatcagtagtattgagtgaccctaacgtgtgaacgcttgtt

tggggaaattataaaaagaaatcagtagtattgagtgaccctaacgtgtgaacgcttgtt

caaaatcaaaaggtttaataataaacagtggtggtagtacttatgcataccttacaagac

caaaatcaaaaggtttaataataaacagtggtggtagtacttatgcataccttacaagac

gatactatagcagcgttaccagtacgtacataaccgtccaatgagcagtgtactataagg

gatactatagcagcgttaccagtacgtacataaccgtccaatgagcagtgtactataagg

ctcatagtttttatactacgtcataaccatatattcataagagtttacaaactggagtta

ctnnnnnnnnnnnnnnnnnnnnnnnnnnntatattcataagagtttacaaactggagtta

ccatgataccatgatgcaaaccttctcgttgatacaaagctatatagtatgatttacatc

ccatgataccatgatgcaaaccttctcgttgatacaaagctatatagtatgatttacatc

agcattatttcaataactagatttccctaatgtaacctgttgttacaattgcaaagaaca

agcattatttcaataactagatttccctaatgtaacctgttgttacaattgcaaagaaca

gtcgccgaagatgcagaattgacccgaagtgattttgttttatcagagcatgttattttt

gtcgccgaagatgcagaattgacccgaagtgattttgttttatcagagcatgttattttt

agaatgggagcaactatgactgttcatggttagtgtctattttgttcatagcaaagcgta

agaatgggagcaactatgactgttcatggttagtgtctattttgttcatagcaaagcgta

aggatcgtttaaagtctccaccataatcacccttttaagcacacgctgcttttccttgtc

aggatcgtttaaagtctccaccataatcacccttttaagcacacgctgcttttccttgtc

gcttaacctgaaggttcccatgtt

gcttaacctgaaggttcccatgtt

**Original *A.rubens* LGR4: GAUU01052396.1**

ca

ca

aatattgaaataagagacgatcagagtctgctaactgttctgtttcccaagaaaatggag

aatattgaaataagagacgatcagagtctgctaactgttctgtttcccaagaaaatggag


cagtaaaaaagatcctcagattctcatcgatctagtggaaggacgaaggaggcattactg

cagtaaaaaagatcctcagattctcatcgatctagtggaaggacgaaggaggcattactg

 
gaatctcaagttgcaaaattatgactcaagatcgtggccgtcatcatgacgtcactacac

gaatctcaagttgcaaaattatgactcaagatcgtggccgtcatcatgacgtcactacac
 M  T  S  L  H  5
gtaattctgttggtgttcatccttacgcatgtctgctgtggaatgagattgaaacatgaa

gtaattctgttggtgttcatccttacgcatgtctgctgtggaatgagattgaaacatgaa
 V  I  L  L  V  F  I  L  T  H  V  C  C  G  M  R  L  K  H  E 25
gccgtgctgaggaacctacaacgacgttccatagatgttcacaggcattgcggtgaagag

gccgtgctgaggaacctacaacgacgttccatagatgttcacaggcattgcggtgaagag
 A  V  L  R  N  L  Q  R  R  S  I  D  V  H  R  H  C  G  E  E  45
tttccgtgtttgaattcgacgcagtgcgtgcctcaggacgctatctgtgacgggacgccg

tttccgtgtttgaattcgacgcagtgcgtgcctcaggacgctatctgtgacgggacgccg
 F  P  C  L  N  S  T  Q  C  V  P  Q  D  A  I  C  D  G  T  P  65
gactgcgacaacgggtctgatgaatgggaaaaggaggaatgtaatgatttccacacgaat

gactgcgacaacgggtctgatgaatgggaaaaggaggaatgtaatgatttccacacgaat
 D  C  D  N  G  S  D  E  W  E  K  E  E  C  N  D  F  H  T  N  85
cagttgtgggacgcaatgtttggtgagaaagatgattgcgatgattcagaagaagatgat

cagttgtgggacgcaatgtttggtgagaaagatgattgcgatgattcagaagaagatgat
 Q  L  W  D  A  M  F  G  E  K  D  D  C  D  D  S  E  E  D  D 105
gacgatgattgcgatgatgatgactcagccagttatcaaaacgaggctggcaactttcta

gacgatgattgcgatgatgatgactcagccagttatcaaaacgaggctggcaactttcta
 D  D  D  C  D  D  D  D  S  A  S  Y  Q  N  E  A  G  N  F  L  125
atcgaggctccatgcgagaacggaacattccccgagacttgcgagtgcatcgtcgaacta

atcgaggctccatgcgagaacggaacattccccgagacttgcgagtgcatcgtcgaacta
 I  E  A  P  C  E  N  G  T  F  P  E  T  C  E  C  I  V  E  L  145
gagaagccctcagcggctcaccgcccggtgagattctcaactctgcccgctgaatctcca

gagaagccctcagcggctcaccgcccggtgagattctcaactctgcccgctgaatctcca
 E  K  P  S  A  A  H  R  P  V  R  F  S  T  L  P  A  E  S  P  165
acaggtccggacgacacatcggaaacaccttcataccgttcaacctctactacaggaaca

acaggtccggacgacacatcggaaacaccttcataccgttcaacctctactacaggaaca
 T  G  P  D  D  T  S  E  T  P  S  Y  R  S  T  S  T  T  G  T  185
ggcacgcagtcaccggtcgttgaagggacagtcattggtgttaagattgactgcagagcc

ggcacgcagtcaccggtcgttgaagggacagtcattggtgttaagattgactgcagagcc
 G  T  Q  S  P  V  V  E  G  T  V  I  G  V  K  I  D  C  R  A  205
agtggactcacgtcgttcccaagaaacctcccagagaacacactatttatagacgtttcc

agtggactcacgtcgttcccaagaaacctcccagagaacacactatttatagacgtttcc

 S  G  L  T  S  F  P  R  N  L  P  E  N  T  L  F  I  D  V  S  225
gacaacaagataaccgagctggcaagagacgactttgcaaatctaacccagttgcgaata

gacaacaagataaccgagctggcaagagacgactttgcaaatctaacccagttgcgaata
 D  N  K  I  T  E  L  A  R  D  D  F  A  N  L  T  Q  L  R  I  245
ttatcgttgtcaaggaacaaactgcgtcacatcgacgatgaagtctttaatccacttaca

ttatcgttgtcaaggaacaaactgcgtcacatcgacgatgaagtctttaatccacttaca
 L  S  L  S  R  N  K  L  R  H  I  D  D  E  V  F  N  P  L  T  265
gaattggaaagattagatatgattgcgtgtggtttggacgaaatacctgcaagattcttc

gaattggaaagattagatatgattgcgtgtggtttggacgaaatacctgcaagattcttc
 E  L  E  R  L  D  M  I  A  C  G  L  D  E  I  P  A  R  F  F  285
gcctcacaaagtagactgaaatacctaaaattagctcacaacaatctgaaaaccctcgct

gcctcacaaagtggactgaaatacctaaaattagctcacaacaatctgaaaaccctcgct
 A  S  Q  S  G  L  K  Y  L  K  L  A  H  N  N  L  K  T  L  A  305
cggatgagcctaattgggctggattctctggttgaccttgatgttcgcggtaaccagatt

cggatgagcctaattgggctggattctctggttgaccttgatgttcgcggtaaccagatt
 R  M  S  L  I  G  L  D  S  L  V  D  L  D  V  R  G  N  Q  I  325
tctgatttggaggtcggagtgtttgaacatactcctcgactcttcaccatacatttttct

tctgatttggaggtcggagtgtttgaacatactcctcgactcttcaccatacatttttct
 S  D  L  E  V  G  V  F  E  H  T  P  R  L  F  T  I  H  F  S  345
gaaaatagactgtcttcgattccagcatccctcctgaggccactgtggaatctcaactgg

gaaaatagactgtcttcgattccagcatccctcctgaggccactgtggaatctcaactgg
 E  N  R  L  S  S  I  P  A  S  L  L  R  P  L  W  N  L  N  W  365
atatctttccaaagaaacgatatcagcaatatagaaaaaggcgccttctctacaaatgag

atatctttccaaagaaacgatatcagcaatatagaaaaaggcgccttctctacaaatgag
 I  S  F  Q  R  N  D  I  S  N  I  E  K  G  A  F  S  T  N  E  385
atgctaactactttaatgttggccgataacaagctgactgctgtcacacgaggtgtgttt

atgctaactactttaatgttggccgataacaagctgactgctgtcacacgaggtgtgttt
 M  L  T  T  L  M  L  A  D  N  K  L  T  A  V  T  R  G  V  F  405
cacaacttgaccaacctggtgttgctgaccttacggaacaacagtatacgacgttttgaa

cacaacttgaccaacctggtgttgctgaccttacggaacaacagtatacgacgttttgaa
 H  N  L  T  N  L  V  L  L  T  L  R  N  N  S  I  R  R  F  E  425
gaaggagcattcgacggaatgacaaagcttcaaacactcaaactgactacgaatccgttc

gaaggagcattcgacggaatgacaaagcttcaaacactcaaactgactacgaatccgttc
 E  G  A  F  D  G  M  T  K  L  Q  T  L  K  L  T  T  N  P  F  445
acatcgcttccacttcgcatatttgacaggttgacgagattacagaaaatctacttcgat

acatcgcttccacttcgcatatttgacaggttgacgagattacagaaaatctacttcgat
 T  S  L  P  L  R  I  F  D  R  L  T  R  L  Q  K  I  Y  F  D  465
catttctcgctgtgtggatacgcccctcacgtccggctctgtatgccgaagagtgacggc

catttctcgctgtgtggatacgcccctcacgtccggctctgtatgccgaagagtgacggc
 H  F  S  L  C  G  Y  A  P  H  V  R  L  C  M  P  K  S  D  G  485
atctcaacagccgagaatctcctggccaatcaccttctccgcttcggggtctggtttgtg

atctcaacagccgagaatctcctggccaatcaccttctccgcttcggggtctggtttgtg
 I  S  T  A  E  N  L  L  A  N  H  L  L  R  F  G  V  W  F  V  505
gctctccttgcctccgtgggcaacgccttcgtcctcttcgcccgctgcttcgtcaaagag

gctctccttgcctccgtgggcaacgccttcgtcctcttcgctcgctgcttcgtcaaagag
 A  L  L  A  S  V  G  N  A  F  V  L  F  A  R  C  F  V  K  E  525
gacaagaagacgcactcattcttcatcatgaacctggctgtggctgacctcttgatgggg

gacaagaagacgcactcattcttcatcatgaacctggccgtggctgacctcttgatgggg
 D  K  K  T  H  S  F  F  I  M  N  L  A  V  A  D  L  L  M  G  545
ctatatctcctcattatcggcacccatgacgtcatcttcagagggacgtacatccttcac

ctatatctcctcattatcggcacccatgacgtcatcttcagagggacgtacatccttcac
 L  Y  L  L  I  I  G  T  H  D  V  I  F  R  G  T  Y  I  L  H  565
gaccttgcctggaggaccagtgtgatctgcaaacttggcgggttccttagtctcctctca

gaccttgcctggaggaccagtgtgatctgcaaacttggcgggttccttagtctcctctca
 D  L  A  W  R  T  S  V  I  C  K  L  G  G  F  L  S  L  L  S  585
agcgaggtgtcaatcatgacgctagcggtcatcacaatggatcgattcctgagtatcgtc

agcgaggtgtcaatcatgacgctagcggtcatcacaatggatcgattcctgagtatcgtc
 S  E  V  S  I  M  T  L  A  V  I  T  M  D  R  F  L  S  I  V  605
cacccttttcggtttaagaatcgaagtctgcttcatgcgcgtctccttatggcgttctta

cacccttttcggtttaagaatcgaagtctgcttcatgcgcgtctccttatggcgttctta
 H  P  F  R  F  K  N  R  S  L  L  H  A  R  L  L  M  A  F  L  625
tggctgcttggtatcgctctgggaacgctacctcttgtgcacttgacatacttcggggac

tggctgcttggtatcgctctgggaacgctacctcttgtgcacttgacatacttcggggac
 W  L  L  G  I  A  L  G  T  L  P  L  V  H  L  T  Y  F  G  D  645
ttatactatggaggcaatggtgtctgcttacccttacagatcgaccagccgttctcgaac

ttatactatggaggcaatggtgtctgcttacccttacagatcgaccagccgttctcgaac
 L  Y  Y  G  G  N  G  V  C  L  P  L  Q  I  D  Q  P  F  S  N  665
ggttgggagttctcactcgtcatcttcgttgtgttcaacctcgtagccttcatgttcatc

ggttgggagttctcactcgtcatcttcgttgtgttcaacctcgtagccttcatgttcatc
 G  W  E  F  S  L  V  I  F  V  V  F  N  L  V  A  F  M  F  I  685
tcatacgcgtacctcatgatgttcgtgaccattcgaaagtccaatctggccatgagatcg

tcatacgcgtacctcatgatgttcgtgaccattcgaaagtccaatctggccatgagatcg
 S  Y  A  Y  L  M  M  F  V  T  I  R  K  S  N  L  A  M  R  S  705
accaagaagaaccaagactgggccctggtgaagaggtttactctcattgtggcaacagac

accaagaagaaccaagactgggccctggtgaagaggtttactctcattgtggcaacagac
 T  K  K  N  Q  D  W  A  L  V  K  R  F  T  L  I  V  A  T  D  725
ttgctgtgctggatgccgatcatagtcgtcaaattcattgccctcggtggtgtgcttgta

ttgctgtgctggatgccgatcatagtcgtcaaattcattgccctcggtggtgtgcttgta
 L  L  C  W  M  P  I  I  V  V  K  F  I  A  L  G  G  V  L  V  745
tcacagagtgtatacgcgtggttcgccatctttgtactcccaatcaactccgccctaaac

tcacagagtgtatacgcgtggttcgccatctttgtactcccaatcaactccgccctaaac
 S  Q  S  V  Y  A  W  F  A  I  F  V  L  P  I  N  S  A  L  N  765
cccattctctataccatgacgacggtcctcttcaaacagaaagtcctggcacctctgggt

cccattctctataccatgacgacggtcctcttcaaacagaaagtcctggcacctctgggt
 P  I  L  Y  T  M  T  T  V  L  F  K  Q  K  V  L  A  P  L  G  785
attgtcagaacgcaacggaagaagggatacatcaccggcacgtccgtcgatgaaacatct

attgtcagaacgcaacggaagaagggatacatcaccggcacgtccgtcgatgaaacatct
 I  V  R  T  Q  R  K  K  G  Y  I  T  G  T  S  V  D  E  T  S  805
tccggtgtgtctaagacctcggggacgaggttgtcgatcatctcgaccaaatctcgtggt

tccggtgtgtctaagacctcggggacgaggttgtcgatcatctcgaccaaatctcgtggt
 S  G  V  S  K  T  S  G  T  R  L  S  I  I  S  T  K  S  R  G  825
gggtcctggaacgggaggctgagctcacacaaggcacaatcggtaaagcgaagttgagga

gggtcctggaacgggaggctgagctcacacaaggcacaatcggtaaagcgaagttgagga
 G  S  W  N  G  R  L  S  S  H  K  A  Q  S  V  K  R  S  **★**   844
actccgaggactctttcaatggttcggtgggccaatcgtactcaaagaaaaacaactacc

actccgaggactctttcaatggttcggtgggccaatcgtactcaaagaaaaacaactacc
 
aagaactacccacttctgatccgatgctcccaaagtcgccttgcagatgtgtaactgatg

aagaactacccacttctgatccgatgctcccaaagtcgccttgcagatgtgtaactgatg
 
acgacaatgctgactaaaaagctggagttggtggggagggggaactgtgactattttggg

acgacaatgctgactaaaaagctggagttggtggggagggggaactgtgactattttggg

tccggtttccctgcatgctgctacgattatgaatattcaggagtcatagaccatgtgact

tccggtttccctgcatgctgctacgattatgaatattcaggagtcatagaccatgtgact

tgtgacatcacaatgtttgcagaagagccacagagc

tgtgacatcacaatgtttgcagaagagccacagagc

**N-terminally modified and codon optimized *A. rubens* RXFP/LGR3**

Kozak Bovine prolactin signal sequence

gccacc atg gac agt aag ggc tca agt cag aaa gga tct agg ctg ctc ctc ctc ctg gta gta tca 
  M   D   S   K   G   S   S   Q   K   G   S   R   L   L   L   L   L   V   V   S  
 aac ctc ctg ctt tgc cag ggc gtg gtg agc gat tat aaa gat gat gac gat aaa 
  N   L   L   L   C   Q   G   V   V   S   D   Y   K   D   D   D   D   K  
 tcc gaa gat gac gca tat gca gtt aca gtt agc gca gaa tat gag 
  S   E   D   D   A   Y   A   V   T   V   S   A   E   Y   E  
 atg aat ggg aac gtt ggc gat ttt acc tgc cct caa ggt gag ttc agg tgt aat aat ctg 
  M   N   G   N   V   G   D   F   T   C   P   Q   G   E   F   R   C   N   N   L  
 act agg tgc gta gca caa cag ttc cag tgt aac aag att gat gat tgt ggt aac aac gca 
  T   R   C   V   A   Q   Q   F   Q   C   N   K   I   D   D   C   G   N   N   A  
 gat gaa atg gaa tgc gag cat gat gaa gga tgg atc aag aca ttc gat gaa aaa att ccc 
  D   E   M   E   C   E   H   D   E   G   W   I   K   T   F   D   E   K   I   P  
 atc agg gtg caa cca aag cgg gag cct tca aca gaa tgt tct ctc tct ggt ttc cct gat 
  I   R   V   Q   P   K   R   E   P   S   T   E   C   S   L   S   G   F   P   D  
 gtt tgc atc tgc tac gag aac act agt ctt aaa tgc gta tac gct aac ttg acc aag gtg 
  V   C   I   C   Y   E   N   T   S   L   K   C   V   Y   A   N   L   T   K   V  
 ccc cct ggc atc tca agt aac atc acc cat ctg aat ctg cgt gga aat cag atc att ttc 
  P   P   G   I   S   S   N   I   T   H   L   N   L   R   G   N   Q   I   I   F  
 gaa gac ggg gtt ttc gag ggt tac tcc aac ttg caa tct ttg aat ctg atg gac aac gga 
  E   D   G   V   F   E   G   Y   S   N   L   Q   S   L   N   L   M   D   N   G  
 ata atc gaa ctt cca gta gac gta ttt cgt gga ttg cgc aat ctg gac aaa ctc tat ttg 
  I   I   E   L   P   V   D   V   F   R   G   L   R   N   L   D   K   L   Y   L  
 tca gat aat aag atc aag ttt ttg att ccc gga act ttt aga tat ctt aac aat gta acc 
  S   D   N   K   I   K   F   L   I   P   G   T   F   R   Y   L   N   N   V   T  
 tgg ctt ttc atg agt aat aac gaa ctc gct gct ctt gga gaa gga gta ttc caa ggg atg 
  W   L   F   M   S   N   N   E   L   A   A   L   G   E   G   V   F   Q   G   M  
 gac aaa ttg tat tgg ctc atg ctc ccc aac aat tct att agt agt ctt gct aga tcc gtc 
  D   K   L   Y   W   L   M   L   P   N   N   S   I   S   S   L   A   R   S   V  
 gcc ttt cgg gat act cct agt ctg gta tgg ttc gac atc agc gaa aac cct ttg ggt agg 
  A   F   R   D   T   P   S   L   V   W   F   D   I   S   E   N   P   L   G   R  
 ctg tca cct tac aac ttc tcc ttg cca ggg aac gtg agt atc atc tcc ttg ttg acc atc 
  L   S   P   Y   N   F   S   L   P   G   N   V   S   I   I   S   L   L   T   I  
 aat aat tgt aac atc agt agt atc cat cgc gat act ttt cga tct ttc agg ggt ctc tcc 
  N   N   C   N   I   S   S   I   H   R   D   T   F   R   S   F   R   G   L   S  
 gtc ctg cac ctc tct cac aat tct att aaa tat ttt cct agt ggt ctc ttc cgg aac ctt 
  V   L   H   L   S   H   N   S   I   K   Y   F   P   S   G   L   F   R   N   L  
 agc aag ctt gct gat ttg cat ata gac aat aac cct gcc act agt ttg cct gag gac ctt 
  S   K   L   A   D   L   H   I   D   N   N   P   A   T   S   L   P   E   D   L  
 ttc gat ggg ctg cat cgt att gat gta ctt gac ctg caa ggg atg acc atc act aac att 
  F   D   G   L   H   R   I   D   V   L   D   L   Q   G   M   T   I   T   N   I  
 aac agc cgg atg ttc gag gag ctc tca aca ttg cag cat atc gaa ttt agt aaa ttt gac 
  N   S   R   M   F   E   E   L   S   T   L   Q   H   I   E   F   S   K   F   D  
 tat tgt cgg tac gca cca cat gtt cgg act tgc aaa cca cga agc aat ggg atc agt agt 
  Y   C   R   Y   A   P   H   V   R   T   C   K   P   R   S   N   G   I   S   S  
 ttc gag gat ctc ctg aaa gat gga atc ctt agg gtt tcc gtc tgg aca att gca ctt ttg 
  F   E   D   L   L   K   D   G   I   L   R   V   S   V   W   T   I   A   L   L  
 tgc ttt ttc ggc aat gta gga gta ctt ata tcc cgc ttt atg atg aaa gca gaa aac cga 
  C   F   F   G   N   V   G   V   L   I   S   R   F   M   M   K   A   E   N   R  
 atc cac agc ctg gtc gta att aac ctg tgt acc gct gac ttc ttt atg agt ata tac ctg 
  I   H   S   L   V   V   I   N   L   C   T   A   D   F   F   M   S   I   Y   L  
 att att att gga ttt cat gat gtt aaa ttt cgc aat ttt ttc aac atg tac gcc ctt gag 
  I   I   I   G   F   H   D   V   K   F   R   N   F   F   N   M   Y   A   L   E  
 tgg atg caa ggc agc act tgc aaa ttc gca gga ttc ctg gca atg ttt agt tct gaa gtc 
  W   M   Q   G   S   T   C   K   F   A   G   F   L   A   M   F   S   S   E   V  
 tcc gta ttt atg ctg aca ttt atc agc ctt gag agg ttt atc tgt ata gta tat cca tat 
  S   V   F   M   L   T   F   I   S   L   E   R   F   I   C   I   V   Y   P   Y  
 cgc ttg cac cgt ctc aca agc aag gag gca act gtc gtt atg tca gta att tgg ttt ctt 
  R   L   H   R   L   T   S   K   E   A   T   V   V   M   S   V   I   W   F   L  
 ggt gcc ctc gtc gca tgg att cca ctc atc tca gtg gga tat ttt gtt gac ttt tac ggt 
  G   A   L   V   A   W   I   P   L   I   S   V   G   Y   F   V   D   F   Y   G  
 tct aat ggt gta tgt ttt cca ctc cac ata cac gat cca tgg ctt ccc ggc tgg gag tac 
  S   N   G   V   C   F   P   L   H   I   H   D   P   W   L   P   G   W   E   Y  
 tca gca ttt gta ttt ctt ggc ctt aat gcc tcc tgt ttt act gca atc gct att tcc tat 
  S   A   F   V   F   L   G   L   N   A   S   C   F   T   A   I   A   I   S   Y  
 acc ggc atg ttt ata tct atc cag cga aca agg aag gct act act aac atc ggc aag cgg 
  T   G   M   F   I   S   I   Q   R   T   R   K   A   T   T   N   I   G   K   R  
 ggt gat atg aac tac gca aaa cga ttc ctt ttt gtt gta ctc acc gat gct ctg tgt tgg 
  G   D   M   N   Y   A   K   R   F   L   F   V   V   L   T   D   A   L   C   W  
 ttg cca atc gca ata ctg aag ata ctg agt ctg tgc agc tac aag atc ccc gcc acc ctg 
  L   P   I   A   I   L   K   I   L   S   L   C   S   Y   K   I   P   A   T   L  
 tac gga tgg ata ata gta ttc gtg ttg ccc ata aat tcc gcc ttg aac cca ata ttg tac 
  Y   G   W   I   I   V   F   V   L   P   I   N   S   A   L   N   P   I   L   Y  
 aca atc tca aca act agt ttc agt cag tgg ttc cac aaa cat gtt aag ttg cgg aga cgt 
  T   I   S   T   T   S   F   S   Q   W   F   H   K   H   V   K   L   R   R   R  
 ggg gag gga cga ggt tct ctt aga ttt aag aac gag ttt tca agt atg ggg gat ttc acc 
  G   E   G   R   G   S   L   R   F   K   N   E   F   S   S   M   G   D   F   T  
 tat ggt ata agt gat att gag cat aag cct gga gca ata gct gaa tag 
  Y   G   I   S   D   I   E   H   K   P   G   A   I   A   E   *
